# International Guideline on the Diagnosis, Treatment, and Monitoring of Long‐Chain Fatty Acid Oxidation Disorders (LC‐FAOD)

**DOI:** 10.1002/jimd.70251

**Published:** 2026-09-24

**Authors:** Sarah C. Grünert, Kaustuv Bhattacharya, Daniela Karall, Mirjam Langeveld, Fran Rohr, Gepke Visser, Jerry Vockley, Aileen Kenneson, Rani H. Singh, Ute Spiekerkoetter, International LC‐FAOD Guideline Workgroup, Georgianne L. Arnold, Kiera Batten, Terry G. J. Derks, Patrick Forny, Melanie B. Gillingham, Emma Glamuzina, Anita Inwood, Monika Jörg‐Streller, Erle Kristensen, Svetlana Lajic, Alexander Laemmle, Risto Lapatto, Elena Martín‐Hernández, Diego Martinelli, Mel McSweeney, Sonia Moreira, Margot F. Mulder, Anna Nordenström, Bryony Ryder, Marit Schwantje, Ida Vanessa D. Schwartz, Arthavan Selvanathan, Serap Sivri, Maren Thiel, Pilar Quijada‐Fraile, Johan L. K. Van Hove, Yilmaz Yildiz

**Affiliations:** ^1^ Department of General Pediatrics, Adolescent Medicine and Neonatology, Medical Center, Faculty of Medicine University of Freiburg Freiburg Germany; ^2^ Genetic Metabolic Disorders Service Sydney Children's Hospital Network, The Children's Hospital at Westmead Sydney New South Wales Australia; ^3^ School of Medicine University of New South Wales Sydney Australia; ^4^ Trinity School of Medicine Trinity College Dublin Dublin Ireland; ^5^ Department of Pediatrics, Division of Inherited Metabolic Disorders Medical University of Innsbruck Innsbruck Austria; ^6^ Department of Endocrinology and Metabolism, Amsterdam UMC University of Amsterdam, Amsterdam Gastroenterology Endocrinology Metabolism (AGEM) Research Institute Amsterdam the Netherlands; ^7^ Met Ed Consultants Boulder Colorado USA; ^8^ Emma Children's Hospital, Department of Pediatrics, Division of Metabolic Diseases, Amsterdam Gastroenterology Endocrinology and Metabolism, Amsterdam UMC Location University of Amsterdam Amsterdam the Netherlands; ^9^ University of Pittsburgh Schools of Medicine and Public Health Pittsburgh Pennsylvania USA; ^10^ Department of Human Genetics Emory University School of Medicine Atlanta Georgia USA

**Keywords:** consensus, diagnosis, dietary management, guideline, long‐chain fatty acid oxidation disorders, newborn screening, outcome

## Abstract

Long‐chain fatty acid oxidation disorders (LC‐FAOD) are rare inherited defects of mitochondrial β‐oxidation that impair energy generation during fasting or metabolic stress. Clinical manifestations range from neonatal hypoketotic hypoglycemia and cardiomyopathy to hepatopathy, recurrent rhabdomyolysis, and chronic myopathy. Although newborn screening (NBS) enables early detection, management remains inconsistent due to limited evidence, broadening phenotypic spectrum, and regional practice variation. An international multidisciplinary workgroup of 34 members, including clinicians, nurses, dietitians, and patient representatives, systematically reviewed the literature (1990–2022) using PubMed, Embase, and Web of Science. Evidence was graded using an adapted Grading of Recommendations Assessment, Development, and Evaluation (GRADE) approach, and consensus was achieved through a modified Delphi–Nominal Group–Delphi methodology, with agreement defined as ≥ 75%. Especially when evidence was low, expert consensus was the basis for this guideline. Recommendations address diagnosis, dietary management, fasting tolerance, use of medium‐chain fat sources, exercise and illness protocols, pregnancy care, and long‐term monitoring. Evidence quality ranged from very low to moderate. Strong consensus was reached for critical clinical interventions. Standardized approaches for diagnostic work‐up, management during metabolic stress, and follow‐up monitoring were developed to promote uniform care across age groups, disease groups, and phenotypic severity. This international guideline integrates available evidence and expert consensus to provide standardized recommendations for diagnosis, treatment and lifelong management of LC‐FAOD. They aim to harmonize clinical practice, improve patient outcomes, and support global implementation of evidence‐based metabolic care.

**Abbreviations**
AFLP: acute fatty liver of pregnancy
BHB: beta‐hydroxybutyrate
CACT: carnitine‐acylcarnitine‐translocase
CK: creatine kinase
CPT: carnitine palmitoyltransferase
DHA: docosahexaenoic acid
DRI: dietary reference intakes
ECMO: extracorporeal membrane oxygenation
EF: ejection fraction
EFA: essential fatty acid
ERG: electroretinogram
FAF: fundus autofluorescence
GRADE: Grading of Recommendations Assessment, Development, and Evaluation
HELLP: hemolysis, elevated liver enzymes, and low platelet count
IMD: inherited metabolic disorders
IV: intravenous
LCF: long‐chain fat
LC‐FAOD: long‐chain fatty acid oxidation disorders
LCHAD: long‐chain 3‐hydroxyacyl‐CoA dehydrogenase
LV: left ventricular
NBS: newborn screening
NG: nasogastric
OCT: optical coherence tomography
PEG: percutaneous endoscopic gastrostomy
QOL: quality of life
TCA: tricarboxylic acid
TFP: trifunctional protein
VEP: visual evoked potential
VLCAD: very long‐chain acyl‐CoA dehydrogenase

## 1 Introduction

Long‐chain fatty acid oxidation disorders (LC‐FAOD) are a group of inherited metabolic disorders (IMD) characterized by impaired mitochondrial β‐oxidation of long‐chain fatty acids, essential for energy production, particularly during periods of fasting or increased energy demand. These defects result from pathogenic variants in genes encoding enzymes and transport proteins involved in the carnitine cycle and β‐oxidation pathway. LC‐FAOD addressed in this guideline are: carnitine palmitoyltransferase 1a (CPT1a) deficiency (OMIM #255120; ORPHA:156), carnitine‐acylcarnitine‐translocase (CACT) deficiency (OMIM #212138; ORPHA:159), carnitine palmitoyltransferase 2 (CPT2) deficiency (OMIM #255110, #600649, #608836; ORPHA:157), very long‐chain acyl‐CoA dehydrogenase (VLCAD) deficiency (OMIM #201475; ORPHA:26793), long‐chain 3‐hydroxyacyl‐CoA dehydrogenase (LCHAD) deficiency (OMIM #609016; ORPHA:5), and mitochondrial trifunctional protein (TFP/MTP) deficiency (OMIM #609015; ORPHA:746).

Clinically, LC‐FAOD manifest with a broad phenotypic spectrum ranging from prenatal malformations to acute metabolic crises in the first days of life—including hypoketotic hypoglycemia, cardiomyopathy, cardiac arrhythmias, rhabdomyolysis, and hyperammonemia—to chronic, progressive muscle weakness, hepatopathy, cardiac arrhythmias, myopathy, and neuropathy or exertional rhabdomyolysis in later life. Individuals with very mild enzyme deficiencies and newborns identified through population‐based newborn screening (NBS) can remain asymptomatic throughout life or develop symptoms only during periods of high metabolic stress (e.g., illness, fasting, increased activity, environmental temperature extremes, and psychological/emotional stress).

The variability in clinical presentation poses challenges for early diagnosis and management. Advances in NBS have improved early detection, enabling prompt intervention that can mitigate some severe outcomes. It has also led to a broadening of the phenotypic spectrum, particularly for some conditions such as VLCAD deficiency, further complicating the diagnostic and management paradigm. Here, challenges remain in differentiating late‐onset disease from carrier status, with a risk of medicalizing and unnecessarily treating newborns with biochemical abnormalities but not clinical symptoms as patients. Current therapeutic strategies focus on fasting duration and dietary modifications, with supplementation of medium‐chain fat sources to bypass defective pathways. Sick day management plans and monitoring for end‐ organ disease are critical. However, practice variation persists due to limited high‐quality evidence and differences in access to diagnostic and therapeutic resources. Even more challenging are treatment strategies in the increasing number of asymptomatic or mildly affected individuals identified by NBS.

Given the rarity, heterogeneity, and complexity of LC‐FAOD, there is a pressing need for standardized clinical practice guidelines to optimize care, reduce morbidity and mortality, and improve quality of life (QOL) for affected individuals. This guideline, developed with evidence‐ and consensus‐based methodology, is intended to be used by metabolic specialists, pediatricians, neonatologists, intensive care specialists, adult physicians, neurologists, dietitians, gynecologists, nurses, psychologists, laboratory specialists, and pharmacists involved in the diagnosis and care of LC‐FAOD patients. It synthesizes available evidence and expert consensus to provide standardized recommendations for diagnosis, treatment, and long‐term monitoring of patients with LC‐FAOD across the lifespan, supporting consistent, high‐quality care worldwide.

## 2 Methodology and Objectives

The workgroup for this project was comprised of 34 members, including physicians, nurses, dietitians, and a patient/family representative. The workgroup members were divided into three groups (Diagnosis & Monitoring, Treatment, and Long‐term Complications). Two co‐chairs were assigned to each group. The workgroup co‐chairs defined the research questions and identified search terms.

### 2.1 Literature Search

**FIGURE 1:**
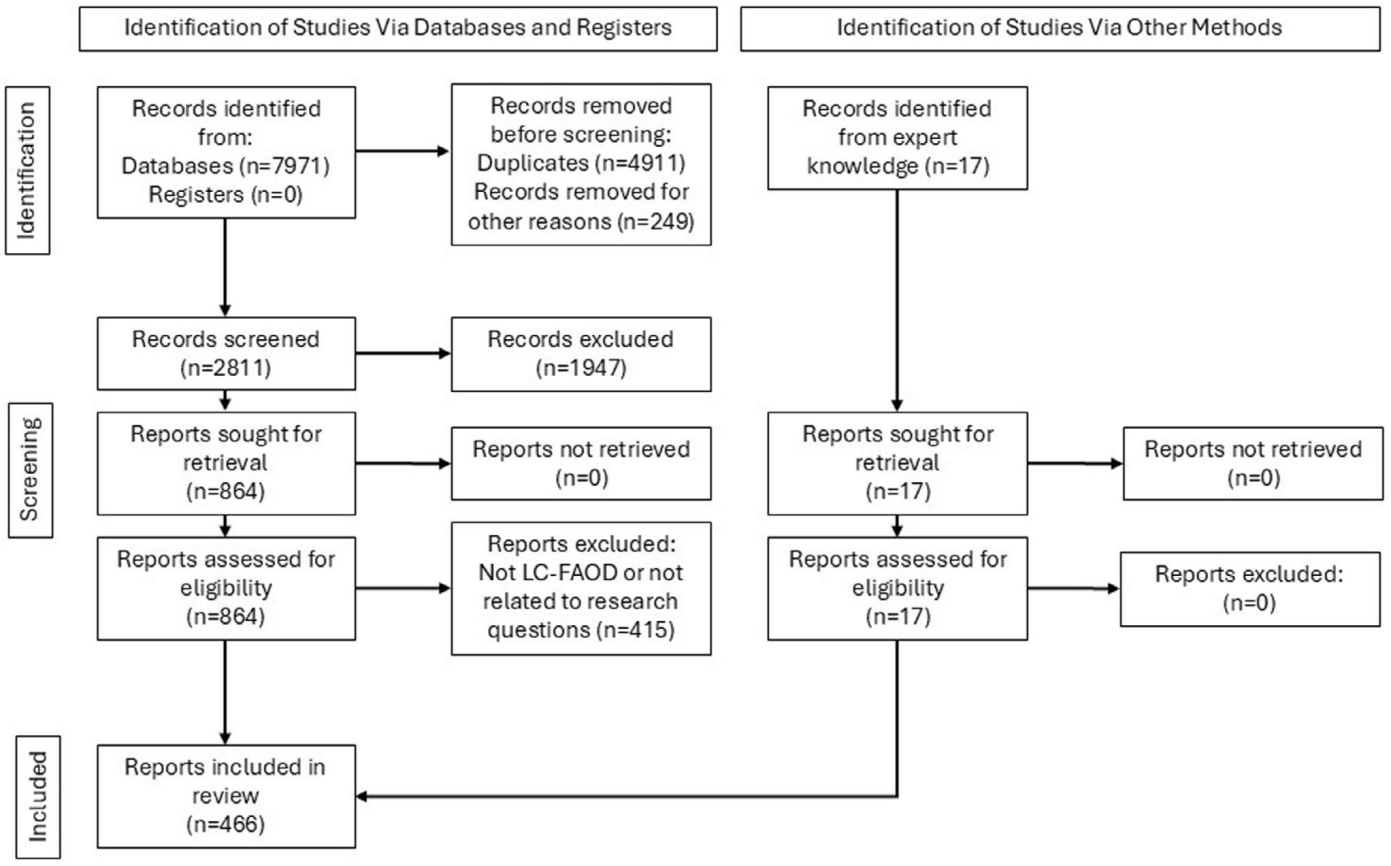
PRISMA flow diagram chart for the literature review conducted as part of the development of the LC‐FAOD guideline.

Searches were performed in three databases: PubMed, Embase, and Web of Science. The initial searches were performed in July 2020 and covered the date range from January 1, 1990, through July 1, 2020. An additional search update was performed in July of 2022 that had a date range of July 1, 2020, through July 1, 2022. Details of the search strategy, including search terms, are available in File S1. In addition, 15 articles and 2 book chapters were identified through expert knowledge that was not captured by the initial search strategy. A list of included literature is available in File S2. The Preferred Reporting Items for Systematic Reviews and Meta‐Analyses (PRISMA) flowchart is presented in Figure 1.

### 2.2 Literature Synthesis and Development of Preliminary Recommendations

Workgroup members assessed the quality of the articles using the modified GRADE methodology [1, 2, 3]. Evidence summaries were drafted for each research question, and preliminary recommendations were drafted. The chairs assigned a strength to each recommendation based on the available evidence, using the following definitions:

- A “Weak” recommendation means that the quality of evidence that exists is suspect (very low or low) OR that well‐done studies (moderate or high) show little clear advantage to one approach versus another.
- A “Fair” recommendation means that the workgroup believes that the benefits exceed the harms (or that the harms clearly exceed the benefits in the case of a negative recommendation), but the quality of evidence is not as strong (moderate). In some clearly identified circumstances, recommendations can be made based on lower‐quality evidence when high‐quality evidence is not available, and the anticipated benefits outweigh the harms.
- A “Strong” recommendation means that the workgroup believes that the benefits of the recommended approach clearly exceed the harms (or that the harms clearly exceed the benefits in the case of a strong negative recommendation), and that the quality of the supporting evidence is excellent/good (high). In some clearly identified circumstances, strong recommendations can be made based on lesser evidence when high‐quality evidence is impossible to obtain, and the anticipated benefits strongly outweigh the harms.

The strength of each recommendation was assigned using a modified GRADE framework based on the balance of benefits and harms, quality of evidence, and certainty in effect estimates [4, 5].

### 2.3 Development of Final Recommendations

The recommendations were reviewed by the workgroup members and finalized using a Delphi‐Nominal Group‐Delphi methodology [6]. Delphi surveys and Nominal Group voting were conducted using a five‐point Likert scale (strongly disagree, disagree, neutral, agree, or strongly agree). Consensus was defined as 75% or more of participants indicating agreement or strong agreement.

The Delphi survey data were collected under a protocol approved by the Emory University Institutional Review Board (IRB) (protocol #00009134).

## 3 Guideline

### 3.1 Diagnosis

#### 3.1.1 What Clinical and Laboratory Work‐Up Should be Performed (in Symptomatic Cases, at Time of Positive NBS in Asymptomatic Cases and After Confirmation of the Disease in Asymptomatic Cases)?

There is a lack of robust evidence for the optimal initial clinical and laboratory work‐up in patients with symptoms suggestive of LC‐FAOD or in asymptomatic individuals with suspected LC‐FAOD who were identified through positive NBS, through family screening or incidentally (e.g., through selective metabolic screening or genetic panel investigations). While larger‐scale studies are absent, the plethora of published case reports and series on LC‐FAOD patients illustrates the wide spectrum of clinical symptoms and laboratory abnormalities patients are at risk of developing.

##### 3.1.1.1 Diagnostic Tests in Symptomatic Cases

LC‐FAOD patients can present with symptoms and biochemical abnormalities prior to the return of NBS results [7, 8]. In LC‐FAOD patients, hypoglycemia, lactic acidosis, and/or relevant elevations of CK have been observed as early as 24 h after birth. Symptomatic TFP deficiency patients with cardiomyopathy, respiratory distress, and hyperammonemia have been observed on the first day of life. In the most severe presentation of CPT2 deficiency, cerebral and renal malformations and cardiomyopathy can be evident prenatally [9]. Later onset cases can also present with rhabdomyolysis or neuropathy (MTPD/LCHADD). In such cases, baseline evaluation for symptomatic cases is indicated in Table 1.

**TABLE 1:** Recommended clinical and laboratory assessment for individuals with LC‐FAOD.

	Diagnostic testing		Monitoring
	Asymptomatic cases (positive NBS + other)	Symptomatic cases	Asymptomatic and symptomatic cases
Laboratory tests^a^			
B‐acylcarnitine profile^b^	X	X	(X)
U‐organic acids	(X)	(X)	—
Molecular genetic testing	X	X	—
Enzyme/functional testing^c^	X	X	—
Fat‐soluble vitamins	—	—	(X)
Essential fatty acids	—	—	(X)
General clinical examination/metabolic evaluation			
Weight, length	X	X	X
Head circumference	X	X	X
Body temperature	X	X	—
Full blood count	X	X	X
Electrolytes	X	X	X
Glucose	X	X	X
Ketones (B/U)	(X)	X	—
Blood gas	(X)	X	—
Lactate	(X)	X	(X)
Ammonia	(X)	X	—
Total and free carnitine	X	X	X
Cardiovascular evaluation			
Cardiac physical examination	X	X	X
Echocardiography	X	X	X
Electrocardiogram	X	X	X
Pro‐BNP	X	X	X
Troponin	X	X	(X)
Liver evaluation			
Hepatomegaly	X	X	X
Hepatic ultrasound	—	(X)	(X)
Transaminases	X	X	X
INR, albumin	—	(X)	(X)
Myopathy evaluation			
Creatine kinase	X	X	X
Myoglobin	(X)	(X)	—
Neurological evaluation			
Muscular tone, strength	X	X	X
Deep tendon reflexes^d^	X	X	X
Peripheral sensation^d^	X	X	X
Electrophysiological studies^d^	(X)	(X)	(X)
Ophthalmologic studies^d^	X	X	X
CNS Imaging^e^	—	X	(X)
Renal evaluation			
Renal function^f^	—	X	X
Renal ultrasound^e^	X	X	(X)

*Note:* X = recommended. (X) = optional. — = not recommended.

^a^ See also Sections 3.1.4 and 3.1.5.

^b^ Many institutions indicated use of acylcarnitine profile to assess free carnitine.

^c^ If molecular results are unclear, or if necessary for severity assessment.

^d^ If LCHADD/TFPD is suspected (do it in other than in neonate).

^e^ If CACTD or neonatal form of CPT2 deficiency is suspected, otherwise at clinical indication.

^f^ If renal dysfunction is suspected because of rhabdomyolysis or in severe CPT2 or CACT deficiency, consider: blood urea nitrogen, creatinine and/or cystatin C along with markers of tubular function.

Although cardiac symptoms are common in most cases of LC‐FAOD, cardiac involvement is not a clinical feature of CPT1 deficiency or the late‐onset myopathic form of CPT2 deficiency, especially caused by the c.338C>T variant. Therefore, individuals with CPT1 and myopathic CPT2 deficiencies do not require cardiac investigations.

##### 3.1.1.2 Diagnostic Tests in Cases Identified by Newborn Bloodspot Screening

Primarily, the aim is to ascertain the patient's physiological status, discerning whether they are metabolically stable or if there is evidence of manifest or impending metabolic decompensation or organ involvement. Baseline investigations are indicated in Table 1. A baseline assessment not only establishes a diagnosis but also facilitates monitoring of disease progression and efficacy of potential treatments.

Targeted diagnostic biochemical, genetic, and/or functional testing to confirm (or exclude) diagnosis are as described in the recommendations in sections 3.1.4 and 3.1.5. False positive NBS results for LC‐FAOD occur and should be identified by follow‐up testing [10, 11]. Several publications report normal subsequent acylcarnitine profiles in infants with an abnormal NBS [12, 13, 14]. False positive and negative NBS results have been reported to be addressed by enzyme/functional assays and/or molecular genetic testing [10, 11, 15, 16, 17, 18, 19].

##### 3.1.1.3 Post‐Mortem and Prenatal Testing

LC‐FAOD have been reported as a cause for sudden infant death syndrome (SIDS) and acute life‐threatening events (ALTE) [20], but the interpretation of acylcarnitine profiles collected post‐mortem can be challenging. Genetic investigations provide clarification, specifically for family planning and genetic counseling.

Pregnancies at risk for carrying a fetus with an LC‐FAOD (typically identified because of a prior affected child) have several options for diagnosis [6]. Optional testing includes amniotic fluid acylcarnitine profiling or amniocyte or placental molecular testing for a known familial genotype. See Section 3.1.2 for prenatal diagnosis. Alternatively, waiting for delivery of the baby and acylcarnitine testing on cord blood, or preferably a sample from the baby by 24 h of age, is appropriate as long as the neonate remains in the nursery, is fed and monitored regularly until the results are available. Note that diagnostic metabolites in cord blood could be minimally abnormal or absent due to clearance by the maternal circulation.

Recommendations:

- Appropriate clinical and laboratory evaluation should be performed in patients suspected of having a LC‐FAOD regardless of the clinical circumstances. The number of possible tests is large and listed in Table 1. Initial clinical assessments should address neurological, hepatic, and cardiac function, while the laboratory evaluation should include biochemical parameters as well as organ function screening, including liver, heart, and muscle parameters.
  Quality of evidence: low.
  Strength of recommendation: strong.
- In (apparently) asymptomatic newborns with positive NBS, the standard of care is to conduct additional biochemical and clinical investigations, including functional testing. Follow‐up testing will also identify false‐positive NBS results.
  Quality of evidence: low.
  Strength of recommendation: strong.
- In neonates with symptoms suggestive of LC‐FAOD, selective metabolic screening is required in addition to NBS.
  Quality of evidence: low.
  Strength of recommendation: strong.
- At‐risk family members of an affected individual should have clinical assessment and testing for possible LC‐FAOD.
  Quality of evidence: low.
  Strength of recommendation: strong.
- LC‐FAOD should be considered in the context of SIDS or ALTE.
  Quality of evidence: low.
  Strength of recommendation: strong.
- Molecular genetic testing should be performed.
  Quality of evidence: low.
  Strength of recommendation: strong.

#### 3.1.2 What Methods Are Available and Recommended for Prenatal Diagnostics?

In the past, prenatal diagnostics has been based on biochemical and enzymatic techniques, but more recently, genetic analysis of fetal DNA has become the standard of care [21]. In families in whom the underlying molecular defect has been established, genetic prenatal testing is the method of choice. Biochemical and/or enzymatic analyses can be used as alternative or additional methods when genetic results are inconclusive (e.g., identification of variants of unknown significance) or unavailable. In some cases, e.g., CPT2 deficiency, acylcarnitine analysis is not sensitive enough to enable a reliable prenatal diagnosis of the metabolic defect [22].

The consideration of individual, cultural, and ethical values regarding prenatal diagnostics is beyond the scope of this guideline, and these aspects should be discussed with the family on an individual basis.

Recommendation:

- Molecular genetic analysis is recommended for the prenatal diagnosis of LC‐FAOD.
  Quality of evidence: very low.
  Strength of recommendation: strong.

#### 3.1.3 What Are the Best Tools for Severity Assessment?

The term disease severity is inconsistently defined in the literature. For the purposes of this guideline, disease severity refers to the frequency and intensity of symptoms. Ideally, assessing disease severity in a patient enables better individualized management and prediction of the clinical course. The body of literature on this topic is still relatively scarce, but several tools exist that are of use in quantifying disease severity.

With some exceptions, genotype/phenotype correlations for most diseases are difficult to predict, including for LC‐FAOD. The common c.338C>T (p.Ser113Leu) variant in *CPT2* is usually associated with an adult‐onset mild phenotype, while inactivating variants result in a severe neonatal form [9, 23, 24, 25]. LCHAD deficiency is caused by homozygosity for the common *HADHA* variant c.1528G>C (p.Glu510Gln); however, despite genetic homogeneity, the clinical phenotype of individuals with LCHAD deficiency can be highly variable. In TFP deficiency, pathogenic variants in the outer protein loops, which are expected to have less impact on protein stability, are usually associated with mild disease [26]. The severity of the clinical presentation is also correlated with the degree of reduction in thiolase antigen [26]. In VLCAD deficiency, evidence suggests milder phenotypes in cases caused by missense variants, especially in the C‐terminus of the protein. The c.848T>C (p.V283A) variant is the most frequent pathogenic variant found in individuals with a positive VLCADD NBS [27]. It is usually associated with onset of rhabdomyolysis in adolescence or early adulthood and not cardiomyopathy. In CACT deficiency, homozygosity for the most common pathogenic splice site variant c.199‐10T>G is associated with a severe neonatal‐onset form of the disease [28].

Measurement of FAO enzyme activities is also helpful, though availability is geographically limited and often available only on a research basis. The level of VLCAD enzyme activity (palmitoyl‐CoA oxidation) measured in lymphocytes or fibroblasts correlates with clinical phenotype [18, 29, 30, 31]. Residual enzyme activity > 20% is consistent with mild/late‐onset disease or even lack of symptoms throughout life; the acylcarnitine profile can still be abnormal [30]. Measurement of cellular LC‐FAO flux has been shown to correlate with the clinical severity of VLCADD patients [32]. Incubation of VLCADD patient‐derived fibroblasts with labeled palmitate produces two distinct profiles of acylcarnitine species that directly correlate with severe early‐onset disease with hypertrophic cardiomyopathy and mild disease manifesting primarily as hypoketotic hypoglycemia [33]. Analysis of cellular acylcarnitine intermediates following incubation with *cis*‐vaccenic, *trans*‐vaccenic, or *trans*‐7‐C18:1 distinguishes milder phenotypes of VLCADD from patients whose course is complicated by cardiomyopathy [34]. In LCHAD and TFP deficiencies, acylcarnitine profile ratios of C18(OH)/[(C14(OH)+C12(OH))] and a fatty acid oxidation flux assay showed positive correlations with the severity of the clinical phenotype [35]. Direct enzyme assays measuring LCHAD and long‐chain 3‐ketothiolase activities in patient cells did not correlate with the clinical phenotype [35].

The Collaborative Laboratory Integrated Reports (CLIR) is an interactive Web Tool created and hosted by the Mayo Clinic [36]. It collects metabolite data from NBS labs around the world and presents them as multiples of the mean of the reporting lab. These values, along with previously demonstrated useful metabolite ratios, can be compared to similar values from other laboratories, including multiple confirmed positive samples for all possible diagnoses, to provide improved sensitivity and specificity compared to results from a single laboratory, though determination of severity is not always possible.

Recommendation:

- Analysis of genetic variants, assessment of enzyme activity and other functional studies, and specialized methods of acylcarnitine profiling can be useful to inform on clinical severity.
  Quality of evidence: low.
  Strength of recommendation: strong.

#### 3.1.4 What Methods Are Available and Recommended for Confirmation of NBS Results?

Diagnostic testing has evolved considerably over the years, and the literature reflects this evolution. Prior to the ready clinical availability of molecular genetic testing, enzyme testing served as the gold standard for confirmation. However, geographic limitations in test availability and a dearth of clinical labs capable of performing the testing led most confirmations to follow the same technology as the NBS (i.e., tandem mass spectrometry characterization of blood acylcarnitine profiles). As molecular genetic testing has become more widely available, especially next‐generation sequencing panels covering all LC‐FAOD disorders or even those included in most NBS programs, it has become the preferred method. However, enzyme analyses, as functional tests, are often superior to molecular genetic testing for severity assessment (see Section 3.1.3). Most reports reviewed did not specifically compare techniques, but large numbers of case series and screening program follow‐ups provide high confidence in molecular techniques, though issues with variants of uncertain significance and non‐exon variants missed by standard existing technology still create ambiguity that requires functional testing in some cases. While most literature reviewed was of moderate quality due to a lack of direct comparison of different modalities, the sheer volume of literature demonstrating the effectiveness of molecular genetic testing raises the recommendation for its use to a strong level.

Recommendation:

- Utilize molecular genetic and enzyme/functional analyses for the confirmation of a LC‐FAOD identified by NBS.
  Quality of evidence: moderate.
  Strength of recommendation: strong.

#### 3.1.5 What Is the Role of Next‐Generation Sequencing in the Diagnostics of LC‐FAOD?

Molecular diagnostic techniques have evolved in keeping with NBS technology. Single‐gene sequencing served as the first molecular gold standard for diagnosing an LC‐FAOD. In recent years, next‐generation sequencing panels covering all LC‐FAOD have become more widely available, shifting the most common method to panels. Numerous publications reported either Sanger or next‐generation sequencing as a follow‐up to metabolite screening, giving a high level of confidence in molecular techniques [37, 38, 39, 40, 41, 42]. There were no direct comparisons of Sanger versus next‐generation sequencing, but nearly all recent literature reported the use of the latter technique. Issues with variants of uncertain significance and non‐exon variants missed with standard existing technology still create ambiguity that requires functional testing in some cases. A direct comparison of exome sequencing in a NBS setting showed traditional metabolite screening to be more sensitive for a LC‐FAOD [43, 44]. Clinical testing, starting with metabolite screening, is still likely to be the frontline test for centers where it is readily available. Most of the literature reviewed was moderate to high quality due to the sheer volume of literature demonstrating the effectiveness of molecular testing.

Recommendation:

- Next‐generation sequencing (NGS), either as a panel or a single gene, can be used for diagnosing an LC‐FAOD as a follow‐up of metabolic screening. It is not yet recommended as the primary NBS.
  Quality of evidence: moderate.
  Strength of recommendation: strong.

### 3.2 Treatment

As noted in the previous section, there is considerable variability in the definition of disease severity for LC‐FAOD. For the purposes of the remainder of this guideline, we have standardized severity categories to support clinical decision‐making rather than serve as rigid diagnostic definitions. Individuals are categorized as having mild/attenuated, moderate or indeterminate, or severe LC‐FAOD based on the combined assessment of clinical history, biochemical findings, molecular results, and, when available, enzyme activity or functional studies.

Mild/attenuated disease refers to individuals without clinical symptoms or prior metabolic decompensation, with normal clinical and laboratory assessments, and supportive genotype or functional data. Moderate or indeterminate‐risk disease includes individuals who are asymptomatic or minimally symptomatic but have persistent disease‐specific biochemical abnormalities, uncertain molecular findings, or unavailable/inconclusive functional testing. Severe disease refers to individuals with neonatal or early/infantile presentation, cardiomyopathy, hypoketotic hypoglycemia, hepatopathy, hyperammonemia, recurrent rhabdomyolysis, hospitalization, and/or genetic/functional findings consistent with severe d**i**sease. In general, CACT, LCHAD, and TFP deficiencies are usually considered to have severe phenotypes. Severity category should be reassessed over time as clinical course, biochemical monitoring, and additional diagnostic information become available.

#### 3.2.1 What Fasting Intervals Are Safe (Age Groups: Infants, Children, Adolescents, Adults)?

Mitochondrial fatty acid oxidation is crucial for energy production, especially during fasting. Thus, LC‐FAOD are considered energy deficiency disorders. Patients with LC‐FAOD are at risk during fasting because they cannot properly oxidize long‐chain fatty acids for energy. Dependency on glycolysis increases, and blood glucose concentrations fall as glycogen stores become depleted. Hypoglycemia is compounded by the lack of ketones as an alternative fuel [45]. Accumulated long‐chain fatty acids and acylcarnitines, or sequestered acyl‐CoA intermediates, may also contribute to acute clinical symptoms [46].

During fasting, several physiological changes have been observed in individuals with LC‐FAOD. In one individual with VLCADD, long‐chain acylcarnitine species increased after a long‐chain triglyceride load at 12 h of fasting [47]. Another experienced rhabdomyolysis and elevated creatine kinase (CK) without hypoglycemia during a 10‐h fasting test, although acylcarnitine levels remained normal [48]. Fasting for more than 15 h led to elevated free fatty acid (FFA) and low ketone levels, with elevated CK and hypoglycemia after 22 h [49].

Fasting intolerance has been reported in LCHAD/TFP deficiencies. In 5 patients with LCHADD, fatty acid intermediates, glycerol, growth hormone, and heart rate increased after 3–4 h of fasting, negatively correlating with glucose, lactate, and pyruvate [50]. A girl with LCHADD showed 50% higher lipolysis and elevated resting energy expenditure versus her healthy sister, with higher acylcarnitines, 3‐hydroxy fatty acids, total FFA, and dicarboxylic acids [51]. In a 15‐year‐old with TFPD, 36 h of fasting induced muscle pain and weakness without hypoglycemia [52]. Therefore, the primary strategy to prevent metabolic decompensation and/or hypoglycemia in LC‐FAOD is avoiding prolonged fasting and ensuring consistent caloric intake, particularly from carbohydrates, medium‐chain fat, and protein. While this applies across all ages and severity levels of LC‐FAOD [53], there is no clear evidence for specific feeding times, and guidance derives mainly from VLCADD literature.

Schedules for maximum fasting intervals include weight‐based schedules (e.g., 1 h per kg of body weight, up to 8 h at 8 kg) and age‐based schedules (e.g., every 3–4 h for the first 4 months, increasing to 12 h by 12 months) [10]. However, there are no specific data supporting any safe fasting interval in well infants; no genotype/phenotype correlations have been investigated to predict safe overnight fasting tolerance [10]. Guidelines for feeding intervals are based on summaries of the literature and expert opinion [45].

In a World Health Organization study, median breastfeeding frequency among compliant infants was 10, 9, 7, and 5 feeds per day at 3, 6, 9, and 12 months, respectively [54]. In suspected LC‐FAOD, this should be the goal. One guideline recommends VLCADD feeding intervals based on age and severity, ranging from 3 h in a severely affected newborn to 12 h in a one‐year‐old with mild or moderate disease [6]. Suggested frequency for individuals with VLCADD: 0 to < 4 months: 3–4 h; 4 to < 6 months: 4–6 h; 6 to < 9 months: 6–8 h; 9 to < 12 months: 8–10 h; ≥ 12 months: 10–12 h. For patients with severe VLCADD, the lower end of the range is recommended. For other severe LC‐FAOD forms, the safe interval must be determined individually, as no evidence guides recommendations.

An exception can be considered for asymptomatic individuals with VLCADD, based on several criteria. For example, the LC‐FAO flux score predicts that patients with > 90% of normal activity will remain asymptomatic without intervention [55]. Similarly, residual VLCAD enzyme activity above 20% of normal is typically associated with late‐onset disease or no symptoms [18, 30]. Without such testing, clinical judgment—based on symptoms, cardiac status, and laboratory findings, particularly normal CK levels—is used to assess asymptomatic status [18, 30].

During illness or metabolic stress, additional precautions should be taken: shorten fasting periods, increase intake of carbohydrate and/or medium‐chain fat sources, and lower overall fat intake. In some cases, continuous enteral feeding might be required [55, 56]. Uncooked cornstarch supplementation at bedtime is generally not indicated for VLCADD patients when they are well, excessive fasting is avoided, and energy needs are met [6]. Providing a bedtime snack that emphasizes complex carbohydrates is recommended to help prevent catabolism.

Recommendations:

- Initiate fasting precautions for neonates suspected of having LC‐FAOD while awaiting confirmation of the diagnosis. Feeding intervals should be no longer than 3 h.
  Quality of evidence: low.
  Strength of recommendation: fair.
- Prevent prolonged fasting through regular, frequent meals and snacks, with specific intervals adjusted based on age, severity, and clinical stability.
  Quality of evidence: low.
  Strength of recommendation: strong.
- During illness, fasting intervals should be shortened further, and dietary management should focus on maintaining adequate carbohydrate intake to prevent metabolic crises.
  Quality of evidence: low.
  Strength of recommendation: fair.
- For patients requiring bedtime feedings to prevent lipolysis, providing a nutritive source of energy, such as complex carbohydrates, is preferred over uncooked cornstarch.
  Quality of evidence: low.
  Strength of recommendation: weak.

#### 3.2.2 What Dietary Measures Are Recommended for Asymptomatic Individuals With LC‐FAOD to Promote Anabolism/Prevent Catabolism in the Long‐Term Treatment (Medium‐Chain Fat Sources, Complex Carbohydrates, That is, Uncooked Starch)?

The literature reviewed included primarily case studies, case series, review articles, and expert opinion addressing dietary management for patients with asymptomatic or mild/attenuated forms of LC‐FAOD, particularly VLCADD.

Observational studies of individuals identified before symptom onset report heterogeneous management and outcomes. Ambrose et al. reported that 9 of 24 individuals who were asymptomatic at diagnosis later developed symptoms, supporting continued monitoring and severity‐based management rather than routine dietary treatment of all asymptomatic individuals [57]. Additional reported outcomes after NBS suggest that dietary management could benefit some patients but insufficient detail is provided to define a uniform dietary prescription for all asymptomatic individuals [58, 59]. Consistent with prior VLCADD nutrition management guidance, long‐chain fat restriction and medium‐chain fat supplementation should therefore be individualized based on the severity categories described above, clinical course, biochemical monitoring, and tolerance [6].

A Delphi survey on VLCADD management concluded that asymptomatic individuals could continue breastfeeding with careful monitoring. For bottle‐fed infants, there was some agreement to switch to a formula with higher medium‐chain fat content, though the exact choice—medium‐chain fat‐enriched versus maximally enriched—did not reach consensus [10].

In VLCADD, LC‐FAO flux scores and residual enzyme activity have been used to inform extent of dietary modification [55]. Patients with an LC‐FAO flux score above 90% were generally asymptomatic and did not require dietary modification, but continuing monitoring of CK and acylcarnitine profiles was recommended. Residual enzyme activity in patient cells indicates disease severity; activity above 10% has been associated with attenuated disease in which essentially unrestricted diet is possible, with modification during metabolic stress when clinically indicated.

Therefore, dietary management should be guided by the severity categories defined above rather than by asymptomatic status alone. Individuals with mild/attenuated diagnosed via NBS or family history might not need dietary fat modification, though some diet modification is often prescribed to prevent metabolic decompensation. In contrast, individuals who are asymptomatic at diagnosis but have uncertain severity, persistent disease‐specific biochemical abnormalities, or molecular/functional findings not clearly consistent with mild disease may require individualized dietary modification [60, 61].

Detailed recommendations for asymptomatic patients suggest a long‐chain fat restriction of 10%–35% of total energy intake, with medium‐chain fat supplementation making up the remaining fat requirement [6]. Medium‐chain fat can be provided via medical formulas or other sources, with specific recommendations varying by patient age, activity level, and tolerance.

Recommendations:

- Patients with mild/attenuated LC‐FAOD (normal labs, absence of clinical symptoms, and, if available, functional studies indicative of a mild phenotype) can follow a normal, unrestricted diet. Laboratory and clinical status should continue to be monitored.
  Quality of evidence: low.
  Strength of recommendation: fair.
- Total fat intake should remain within the recommended range, not exceeding fat intake of 20%–35% of energy intake for adults, 20%–40% for children, and 30%–55% for infants.
  Quality of evidence: low.
  Strength of recommendation: fair.

#### 3.2.3 What Dietary Measures Are Recommended for Symptomatic Patients With LC‐FAOD to Promote Anabolism/Prevent Catabolism in the Long‐Term Treatment (Medium‐Chain Fat Sources, Complex Carbohydrates, or Uncooked Starch)?

There is little empirical evidence on best dietary practices for symptomatic patients with LC‐FAOD; for this reason, most recommendations are based on descriptive case series, case reports, or expert opinions.

The primary goal of nutrition management of LC‐FAOD is to maintain metabolic stability by preventing catabolism, avoiding prolonged fasting, and providing adequate energy during illness, exercise, and other periods of increased energy demand [62]. Dietary long‐chain fat restriction reduces the dietary long‐chain fatty acid load that requires oxidation through the affected pathway, while medium‐chain fat sources provide an alternative energy substrate that can enter mitochondria independently of the carnitine shuttle and bypass the long‐chain β‐oxidation enzymes. The benefit of medium‐chain fat sources is therefore not presumed to result from meaningful changes in adipose triglyceride composition, but rather from providing readily oxidizable fuel and, in some circumstances, supporting ketone body production and reducing reliance on long‐chain fatty acid oxidation. Providing 40%–55% of total energy from fat with 10%–15% from long‐chain fat has been recommended in **infants** with severe disease [6]. However, long‐chain fat intake depends on the individual patient's age, pathogenic variant, activity and disease severity. While medium‐chain fat sources provide an alternative energy source in LC‐FAOD, they do not fully replace long‐chain fatty acid metabolism and pose risks including gastrointestinal intolerance, excess energy intake, and disruption of essential fatty acid balance [61, 63]. In a series of 23 patients with CACT deficiency, neonatal‐onset patients all had restricted LCF between 5% and 10% of total energy intake throughout childhood [64]. See Section 3.2.4 regarding breastfeeding.

After infancy, it has been suggested that LCF be limited to 10%–15% of energy, and that medium‐chain fat sources provide 20%–40% of energy intake. A fat‐modified diet has been reported to resolve cardiomyopathy in some patients [65, 66, 67, 68, 69, 70, 71, 72] but did not prevent retinopathy in LCHAD/TFP patients [69]. For these patients, supplemental docosahexaenoic acid has been recommended, as it appears to slow, but not stop, the progression of eye disease (see Section 3.2.7) [6, 62, 73, 74].

In childhood and adulthood, 20%–40% of total energy from fat in the diet has been recommended. These recommendations are otherwise similar to those for infants; however, long‐chain fatty acids are provided through food sources rather than formula.

In severe disease, limiting long‐chain fat to 10% of total energy allows providing medium‐chain fat sources to 20%–25% of the energy requirement. All other nutrients should be provided in amounts consistent with the dietary reference intakes (DRI). Monitoring protein intake is important, as restricting dietary fat can lead to insufficient protein intake, since many high‐fat foods are also rich in protein.

As infant formulas for LC‐FAOD are enriched with essential fatty acids, deficiency of fat‐soluble vitamins is a risk with subsequent long‐chain fat‐restricted diets [6, 75]. Linoleic acid (C18:2n6) and α‐linolenic acid (C18:3n3) have been recommended to comprise 3% and 1.4% of energy intake, respectively, typically through supplementation with specific oils.

After 12 months of age, the most common recommendation is 25%–35% of calories from long‐chain fat with medium‐chain fat supplementation. More stringent LCF restriction can be considered, allocating less than half of fat calories to LCF and more than half to medium‐chain fat sources.

The dietary macronutrient composition in diets for patients with LC‐FAOD has been investigated. Patients with a higher‐protein diet intake (25%–28% of calories from proteins and 50%–56% from carbohydrates) have increased blood levels of short‐chain acylcarnitines, reduced intrahepatic lipid content, and greater lean body mass compared with patients on a high‐carbohydrate diet (11%–13% protein and 64.74% carbohydrates) [76].

Recommendations:

- In symptomatic infants with LC‐FAOD, nutritional management should provide 40%–55% of total energy from fat, with 10%–15% from long‐chain fat. Symptomatic infants with severe disease require exclusive feeding with formula enriched with a medium‐chain fat source or with skimmed breast milk containing a medium‐chain fat source. Symptomatic infants with mild disease might be able to breastfeed, but it might be necessary to limit it, adding a supplementary metabolic formula containing medium‐chain fat or supplementing pumped breast milk with a metabolic formula.
  Quality of evidence: low.
  Strength of recommendation: fair.
- In symptomatic children and adults with LC‐FAOD, nutritional management should provide 20%–35% of total energy from fat. All other nutrients should be provided in amounts to meet reference intakes. In severe disease, limit long‐chain fat to 10%–15% of total energy, provide medium‐chain fat sources to 20%–25%, or use triheptanoin (an odd‐chain, medium‐chain fat) to 25%–40% of the energy requirement. For individuals with moderate disease, limit LCF to 15%–25% of energy, and provide the remainder of fat from medium‐chain sources.
  Quality of evidence: low.
  Strength of recommendation: fair.
- Linoleic acid (C18:2n6) and α‐linolenic acid (C18:3n3) should comprise 3% and 1.4% of energy intake, respectively, typically through supplementation with specific oils.
  Quality of evidence: low.
  Strength of recommendation: fair.

#### 3.2.4 Who Can be Breastfed/Partially Breastfed?

Breast milk is high in fat content; thus, breastfeeding has been suggested to be replaced in symptomatic infants with a formula enriched with medium‐chain fat [6, 10, 62]. Hesitancy about breastfeeding arises in part from a report of two infants diagnosed with CACTD who were breastfed and died suddenly [77]. In clinically stable children, LCF to cover essential fatty acids has been safely delivered by breastmilk‐supplemented formula and/or breastfeeding.

For neonates with severe LC‐FAOD, discontinuation of breastfeeding or regular infant formula has been recommended, with the addition of a medium‐chain fat source using a medium‐chain fat‐based medical formula with sufficient LCF to meet dietary requirements [6]. Skimmed breast milk, which retains the nutritional and immunological benefits of regular breast milk while having reduced long‐chain fat content, has been used in seriously ill LC‐FAOD patients. A baby with CACTD presenting on the first day of life with hyperammonemia, increased CK, and cardiomyopathy had reported treatment with triheptanoin mixed with skimmed breast milk using a portable cream separator, resulting in residual long‐chain fat closer to 6%–7% of total calories [78]. The cardiomyopathy reversed, and no further metabolic decompensation occurred during the first year of life. Given the nutritional and immunological benefits of breast milk, skimmed breast milk with a supplemental medium‐chain fat source is an excellent option for seriously ill patients, although it adds complexity to nutritional management.

Growing evidence suggests that nutrition management of infants with LC‐FAOD should primarily depend on their clinical status. Infants who remain asymptomatic and have confirmatory tests indicating a milder/later‐onset form of VLCADD can continue to breastfeed without the need for supplemental medium‐chain fats. In a report of five infants with LC‐FAOD, three with LCHADD were fed expressed breast milk, one supplemented with medium‐chain fat for two months without crises, while a symptomatic CPT2D infant and a VLCADD infant were breastfed [79].

Recommendations:

- To the extent possible, include breast milk in the diet of an infant with LC‐FAOD in collaboration with a knowledgeable lactation consultant within the metabolic team:
  ○ For an infant with mild/late‐onset LC‐FAOD (i.e., infant with VLCADD identified via NBS, who is asymptomatic), provide breast milk exclusively (from the breast or expressed).
  ○ For an infant with moderate LC‐FAOD, use a combination of breast milk (expressed or from the breast) and a medical food (modified in LCF and supplemented with medium‐chain fat) to provide 15%–30% of energy as LCF and 10%–30% of energy as medium‐chain fat, with a total fat intake of 35%–55% of energy needs.
  ○ For an infant with severe LC‐FAOD, skimmed breast milk can be used as part of the diet in combination with a medium‐chain fat source to meet fat intake goals.
  ○ For infants fed from the breast, ensure adequate breastmilk intake by closely monitoring the infant's growth and clinical status.
    Quality of evidence: low.
    Strength of recommendation: strong.
- For an infant with severe LC‐FAOD, discontinue breastfeeding and provide a medical food (modified in LCF and supplemented with medium‐chain fat) or a modular formula and medium‐chain fat sources to provide a total fat intake of 40%–50% of energy needs, with 10%–15% of energy as LCF, and 35%–40% of energy as medium‐chain fat.
  If non‐proprietary medical foods are used (i.e., skimmed milk, modular formulas), pay attention to meeting all nutrient needs, not only fat intake goals.
  Quality of evidence: low.
  Strength of recommendation: strong.

#### 3.2.5 What Are the Indications for Tube Feeding?

Tube feeding has been reported to be a helpful intervention for patients who experience severe feeding difficulties. One review studied its role in improving clinical outcomes for these patients. In 3 out of 16 LCHADD patients, tube feeding was used to avoid fasting periods longer than 3–4 h in infants and children when oral intake was inadequate [80]. Patients who received early, strict dietary treatment, including tube feeding, showed improved survival rates and overall better health outcomes compared with earlier cohorts without such treatment. Tube feeding was beneficial, especially in infants and small children, improving weight and dental health, increasing satiety, and reducing daytime feeding. Additional indications for tube feeding are chronic vomiting and feeding refusal [81].

The type of tube (NG, gastric (G‐tube), or gastro‐jejunal) depends on each patient's specific needs and complications, with G‐tubes preferred for long‐term use. Regular monitoring of feeding tubes is needed to ensure proper functioning and integrity.

Recommendation:

- Implement tube feeding in LC‐FAOD patients with severe oral intake issues, such as poor weight gain or frequent vomiting, to ensure consistent nutrient delivery and prevent metabolic crises.
  Quality of evidence: low.
  Strength of recommendation: strong.

#### 3.2.6 What Dietary Measures Are Recommended Before Physical Exercise?

Extensive or sustained exercise can trigger metabolic decompensation in patients with LC‐FAOD. However, exercise restriction is not necessary, and indeed is beneficial for them [82, 83]. The effects of supplementation with a medium‐chain fat source prior to exercise have been compared across different studies. Providing medium‐chain fats before exercise improves their efficiency as an energy source. When taken before exercise, medium‐chain fats increase fat utilization, reduce reliance on glucose, and lower heart rate during steady‐state exercise at the same workload, compared with simple carbohydrates such as juice [84]. When medium‐chain fats are combined with juice, potentially harmful long‐chain intermediates (LC‐3‐hydroxy acylcarnitines) are reduced by about 30%, while levels of ketones (beta‐hydroxybutyrate, BHB)—an alternative energy source—are increased threefold, compared to juice alone [84].

In children with LCHAD deficiency and TFP deficiency, ingestion of medium‐chain fats (0.5 g/kg) ~20 min before periods of increased activity improved metabolic stability after exercise [82]. Coordinating medium‐chain fat supplementation with periods of increased activity improved the ability of children with LCHADD to tolerate exercise and reduced the frequency of rhabdomyolysis episodes [85]. In patients treated with triheptanoin, a dose 30 min before strenuous physical activity has been recommended [86]. Another study suggested the use of complex carbohydrates (0.5–1 g/kg) with or without protein [83]. A study evaluating ketone esters plus dextrose vs. dextrose before exercise demonstrated a suppression of lipolysis [87]. However, ketone esters are not yet available in clinical practice.

Recommendation:

- Exercise should be preceded by energy intake to meet the extra demand. Dietary intervention approximately 10–20 min before exercise can consist of medium‐chain fatty acids (0.5–1 g/kg), triheptanoin (0.5 g/kg), or complex carbohydrates with or without protein.
  Quality of evidence: moderate.
  Strength of recommendation: fair.

#### 3.2.7 Is There Evidence for Treatment With DHA in LC‐FAOD?

See also Section 3.3.7. Supplementation with essential fatty acids (EFAs) and docosahexaenoic acid (DHA) is important for preventing deficiencies and ensuring proper growth and development in LC‐FAOD. Linoleic acid (omega‐6) and α‐linolenic acid (omega‐3) are necessary, as they cannot be synthesized endogenously. Supplementation should aim to meet the body's requirements without burdening the defective metabolic pathway, with reported intake ranging from 2% to 4% of total energy as linoleic acid and 0.3%–1.4% as α‐linolenic acid [6]. The best sources of linoleic acid are safflower oil, sunflower oil, and corn oil, while flaxseed and chia seed oil are the richest sources of α‐linolenic acid. Oils that provide both EFAs in significant amounts include soybean oil, hempseed oil, canola oil, and walnut oil.

DHA is synthesized from α‐linolenic acid and is needed for brain and eye development, cognitive function, and overall neurological health, especially in children. Low concentrations of DHA have been measured in children with VLCAD and LCHAD deficiency [75, 88]. In the face of a severe LCF restricted diet, supplementing pre‐formed DHA directly is often necessary (60 mg/d for individuals weighing under 20 kg, 120 mg/d for individuals weighing over 20 kg) [61].

Recommendations:

- For individuals with LC‐FAOD on a fat‐restricted diet, ensure adequate intake (diet and/or supplements) of EFAs to provide **2%–4% of total energy intake** as linoleic acid, **0.3% to 1.4% of total caloric intake as** α‐linolenic acid.
  Quality of evidence: moderate.
  Strength of recommendation: fair.
- Consider supplementation with DHA if normal DHA concentrations cannot be achieved with essential fat supplementation.
  Quality of evidence: moderate.
  Strength of Recommendation: fair.

#### 3.2.8 Which Laboratory Parameters Should be Assessed to Monitor Nutritional Status? What Are the Indications for Nutritional Protocols?

Effective management of patients with LC‐FAOD requires regular clinical assessments—encompassing medical, nutritional, and psychological evaluations, as well as biochemical monitoring. This comprehensive approach helps detect metabolic imbalances early, guide dietary interventions, and prevent severe complications during periods of illness or stress. The frequency of monitoring depends on the individual's clinical status; routine monitoring has been recommended at diagnosis, every 3 months during the first year or two of life, and every 6–12 months afterwards [89]. The following outlines key biomarkers for monitoring individuals with LC‐FAOD that have been reported to help promote optimal outcomes.

##### 3.2.8.1 Creatine Kinase (CK)

Monitoring CK is critical for detecting muscle damage, particularly rhabdomyolysis. Elevated CK activity, particularly CK‐MM and CK‐MB, signal metabolic decompensation, with activity above 1000 U/L defining rhabdomyolysis and aiding in determining the severity of metabolic crises [6]. Correlations between elevated CK activity and muscle damage in patients with LCHAD and TFP deficiency are also well established and correlate with transaminase and acylcarnitine concentrations during episodes of stress [69].

##### 3.2.8.2 Plasma Carnitine (Free, Total, Esterified)

Monitoring plasma carnitine concentrations is frequently reported in the literature, but there is no direct evidence that correlates to metabolic function or clinical symptoms [6, 69].

##### 3.2.8.3 Plasma Acylcarnitine Profile

Long‐chain acylcarnitines are elevated during acute metabolic decompensation and correlate with CK levels. Outpatient monitoring of disease‐specific acylcarnitines in LC‐FAOD has been suggested. However, plasma acylcarnitine concentrations fluctuate based on physiological states such as fasting, feeding, and exercise. Feeding reduces long‐chain acylcarnitine concentrations, whereas fasting and exercise can increase them. This variability complicates the interpretation of acylcarnitine profiles for routine outpatient monitoring unless sample collection is carefully standardized with respect to these factors [90]. To improve the utility of acylcarnitine profiles in a clinical setting, collecting blood samples under standardized conditions, such as controlling the timing relative to meals and physical activity, has been recommended [6, 91].

##### 3.2.8.4 Essential Fatty Acids

Monitoring EFAs in individuals on an LCF‐restricted diet, including linoleic and α‐linolenic acids, is crucial to ensure nutritional adequacy in LC‐FAOD patients on fat‐restricted diets. Patients with LC‐FAOD can develop deficiencies when long‐chain fatty acid intake is restricted, necessitating regular monitoring [6, 75].

##### 3.2.8.5 Fat‐Soluble Vitamins

Fat‐soluble vitamins (Vitamins A, D, E, and K) should be monitored, and in individuals treated with an LCF‐restricted diet, supplements should be provided if deficiencies develop. Regular assessment of these vitamins helps avoid long‐term complications such as bone density loss or immune dysfunction. Persistent vitamin E and K deficiencies, even with supplementation, have been noted in LCHADD patients, highlighting the importance of vigilant monitoring [6, 75].

Recommendation:

- Monitor the clinical, biochemical and nutritional status of individuals with LC‐FAOD as outlined in Table 1.
  Quality of evidence: moderate.
  Strength of recommendation: strong.

#### 3.2.9 Is There Evidence for Treatment With L‐Carnitine in LC‐FAOD?

Literature on LC‐FAOD does not specifically address the use of L‐carnitine treatment. It has been proposed to conjugate plasma metabolites and replete low carnitine levels [92, 93]. However, concerns have been raised about possible arrhythmogenic effects of long‐chain acylcarnitines, though there are supporting data [94, 95, 96]. There have also been no published clinical trials on the use of L‐carnitine in LC‐FAOD.

The management practices in 18 European metabolic centers have been reviewed, and there was no evidence supporting L‐carnitine supplementation for any LC‐FAOD [61]. Several case series or reports have commented specifically on L‐carnitine supplementation [67, 88, 97, 98, 99, 100, 101, 102]. In a study of LCHADD, in 31 of 50 surviving patients, 12 were treated with L‐carnitine (50–100 mg/kg/day) [103]. There were no statistically significant differences in morbidity (recurrent metabolic attacks and/or muscle cramps) with or without L‐carnitine supplements. In another study of 13 individuals with LCHADD, 8 were treated with carnitine, 12 patients died during follow‐up, with no difference in symptoms or survival correlated with carnitine treatment [104]. Two brothers with VLCADD have been reported to have fewer rhabdomyolysis episodes when supplementation with L‐carnitine was ceased [105].

Of 52 patients with proven LC‐FAOD participating in a clinical trial of treatment with triheptanoin, all but two CPT1 patients received supplemental L‐carnitine (100 mg/kg/day, divided in 4 daily doses, max 1000 mg/day) [106]. Patients included 8 infants, 28 children, 7 adolescents, and 9 adults representing the following disorders: 2 CACT, 11 CPT2, 21 VLCAD, 6 TFP, and 10 LCHAD deficiencies. No obvious adverse events related to carnitine supplementation were reported [106].

Recommendation:

- There is no evidence to support supplementation with L‐carnitine in LC‐FAOD, nor does any evidence document adverse effects.
  However, if total and free carnitine levels are very low, a low dose (10 mg/kg/day for 4–6 weeks) can be considered, with monitoring and discontinuation when levels normalize.
  Quality of evidence: low.
  Strength of recommendation: fair.

#### 3.2.10 Is There Evidence for Treatment With Triheptanoin in LC‐FAOD?

Treatment with triheptanoin (marketed in the USA by Ultragenyx as Dojolvi) was first suggested for patients with LC‐FAOD who have persistent myopathy or cardiomyopathy despite treatment with conventional medium‐chain fat preparations that contain predominantly even‐carbon oils [107, 108]. In vitro studies have demonstrated deficits in tricarboxylic acid (TCA) cycle intermediates, particularly succinate, in patient‐derived cells and in LC‐FAOD animal models. Acetyl‐CoA from medium‐chain fat sources addresses only a deficit of oxaloacetate and citrate. However, triheptanoin generates both acetyl‐CoA and the 3‐carbon propionyl‐CoA that enters the TCA cycle as succinyl‐CoA [108]. In this manner, triheptanoin provides an appropriate substrate balance for the TCA cycle (anaplerotic effect).

The first report of its use was in three children with VLCADD previously supplemented with MCT oil. Treatment with triheptanoin led to marked improvement in muscle strength and reduction in rhabdomyolysis in all patients and improved cardiomyopathy in one [107]. The frequency of rhabdomyolysis also fell. Treatment with triheptanoin was also reported in seven patients with myopathic CPT2 deficiency. Before starting triheptanoin, all patients had muscle pain on exercise, and six required hospital admission at least once for rhabdomyolysis. Triheptanoin was included in the diet (30% of total energy intake) and taken before exercise for 7–61 months and almost completely prevented muscle pain. The only two hospital admissions occurred when patients had temporarily stopped taking the oil [109].

Since then, further clinical trials have demonstrated that triheptanoin reproducibly resulted in decreased hypoglycemia, rhabdomyolysis, and cardiomyopathy event rates as well as decreased hospitalizations and total hospital days in patients with LC‐FAOD. They cover well over 100 patients with different LC‐FAOD [63, 106, 109, 110, 111, 112, 113, 114, 115]. In a short‐term study (4 months), double‐blind comparison of triheptanoin versus trioctanoin in 32 patients with LC‐FAOD, subjects in the C7 group increased left ventricular (LV) ejection fraction (EF) while experiencing a decrease in LV wall mass on their resting echocardiogram though they were normal at the start of the study [110]. Medium‐chain fat sources and triheptanoin were administered to 12 patients with LC‐FAOD in combination, with no obvious adverse effects [113].

There are two published regimens for the use of triheptanoin. First, it can replace all medium‐chain fatty acids at a dosage of 2–4 g/kg/day (up to 35% of total dietary calories) [116]. It has also been prescribed at a dose of 1–2 g/kg/day in combination with medium‐chain fat [113], which allows more flexibility when mixed with commercially available formulas. Medium‐chain triglyceride preparations have been used in cooking or mixed with solid foods as long as temperatures do not exceed 200°C (400°F). Tubes made from polyvinyl chloride should be avoided when administering triheptanoin.

Since the completion of the evidence review for this question, triheptanoin has been approved for the treatment of LC‐FAOD patients in the US and other countries. However, longer‐term evidence was not available for broader consideration of its efficacy in this guideline.

Recommendation:

- Treatment with triheptanoin can be a part of the therapy in symptomatic patients with LC‐FAOD.
  Quality of evidence: moderate.
  Strength of recommendation: strong.

#### 3.2.11 What Are the Criteria for Hospital Admission?

Criteria for hospital admission are not published, and thus the evaluation of this question relied on published case series and case reports describing the symptoms that led to hospital admission.

Sick day protocols that alert parents to symptoms of imminent metabolic decompensation have been recommended (see Section 3.2.12) [6], along with avoidance of situations that induce catabolism, including excessive fasting or intervals between meals, and inadequate calorie support for exercise or physical activity. Increased energy intake, with reduced long‐chain fat intake during intercurrent illnesses, has also been noted to be important [117, 118].

Symptoms reported to lead to hospitalization include cold sweating and other signs of possible hypoglycemia, vomiting, lack of appetite, somnolence, lethargy, seizures, abdominal and/or muscular pain, dark‐colored urine, high or low heart rate, arrhythmias, and pallor. Laboratory and clinical findings that have resulted in hospitalizations include hypoglycemia, hyperammonemia, elevated CK, and deteriorating cardiac function.

Recommendations:

- Parents and patients should be aware of the presenting symptoms of imminent metabolic decompensation.
  Quality of evidence: low.
  Strength of recommendation: strong.
- Catabolic situations should be recognized so they can be prevented or foreseen with countermeasures taken.
  Quality of evidence: low.
  Strength of recommendation: strong.
- Symptoms and laboratory testing indicative of hypoglycemia, metabolic acidosis, rhabdomyolysis, or organ involvement (liver, heart, muscle) should trigger hospital admission.
  Quality of evidence: low.
  Strength of recommendation: strong.

#### 3.2.12 Sick Day Protocol for Intercurrent Illnesses at Home: When to Start and Which Regimen?

Published sick‐day management involves frequent meals and increased carbohydrate intake to prevent catabolism. LCF restriction is usually recommended to reduce the accumulation of toxic metabolites, along with early antipyretic treatment to minimize energy expenditure associated with febrile infections. These measures are based on the pathophysiology of the diseases and have not been studied in controlled clinical trials [6, 119, 120, 121].

Although there is little information in the literature on the efficacy of sick day protocols/emergency letters for LC‐FAOD, Rossi et al. have studied the use of generic emergency protocols for glycogen storage diseases and FAOD in a single center [117]. This study included only a few individuals with LC‐FAOD, but the emergency protocols were found to be safe and easy to use for home management by the caregivers and during the first hour in‐hospital management.

Emergency letters for individual patients with LC‐FAOD can be generated at the website www.emergencyprotocol.net in different languages. This website is freely accessible to patients and healthcare providers [117].

Recommendations:

- Sick day protocols/emergency letters should be provided to LC‐FAOD patients and their caregivers to help with early management in acute situations, reducing the need for hospitalization and clinical complications.
  Quality of evidence: low.
  Strength of recommendation: strong.
- Sick day regimens at home should include frequent meals, long‐chain fat restriction, increased energy intake, and early antipyretic treatment in order to prevent catabolism.
  Quality of evidence: low.
  Strength of recommendation: strong.

#### 3.2.13 What Is the Recommended Nutritional and Medical Management During Hospitalization?

Published hospital management has largely been case‐based, with single case reports or small case series. LCF restriction is usually recommended to reduce the accumulation of toxic metabolites [6, 117, 119, 120, 121]. These treatments are based on the pathophysiology and have not been formally studied in clinical trials. Most publications focus on acute medical management while providing little specific nutritional information.

A few NBS follow‐up articles have demonstrated that nutritional intervention (i.e., initiation of oral feeding) is sufficient to treat asymptomatic infants prior to discharge and during transition to outpatient management. For acutely ill hospitalized patients, there is agreement that intravenous (IV) fluids should be given to deliver supraphysiologic calories (most commonly a 1.5‐times‐maintenance glucose infusion) plus appropriate electrolytes. Some patients have been reported to continue enteral intake, including a medium‐chain fat source, depending on their level of consciousness and ability to tolerate enteral nutrition. Maintenance of IV therapy until blood CK levels begin to decline is reported in some studies, but there are no data to suggest a specific level for discontinuation. Respiratory (intubation) and cardiac (extracorporeal membrane oxygenation, ECMO) support are reported in numerous critically ill cases. Additional supplementation with carnitine and (less frequently) riboflavin has been used, though there are no data to support efficacy unless deficiency is present. Concerns about the toxicity of long‐chain acylcarnitines have been raised in some publications, but no literature supports them. Particularly dramatic case and case series publications report the use of triheptanoin in patients with acute cardiac failure (typically in those already on standard medium‐chain fat supplementation) and are compelling in terms of efficacy but are uncontrolled.

Recommendations:

- Hospital management should be managed by metabolic experts and should include the administration of IV glucose to restore an anabolic state, along with appropriate electrolytes to maintain normal blood levels.
  Quality of evidence: low.
  Strength of recommendation: strong.
- Standard rescue therapies such as ventilator support and ECMO should follow institutional standards of care for initiation and discontinuation.
  Quality of evidence: low.
  Strength of recommendation: strong.
- There is insufficient evidence to provide a recommendation on the use of carnitine (unless deficient) or riboflavin in an acutely ill patient.
  Quality of evidence: low.
  Strength of recommendation: strong.
- Triheptanoin supplementation can be utilized in acutely ill children, even in patients supplemented with medium‐chain fat.
  Quality of evidence: low.
  Strength of recommendation: strong.

#### 3.2.14 What Special Treatment Recommendations Exist for LC‐FAOD and Pregnancy?

##### 3.2.14.1 HELLP/AFLP

Literature on LC‐FAOD in pregnancy focuses on the association between neonatal LCHAD deficiency and maternal complications such as HELLP (hemolysis, elevated liver enzymes, and low platelet count) syndrome and Acute Fatty Liver of Pregnancy (AFLP). The presence of LCHAD deficiency in the fetus has been linked to liver disease in pregnant persons. For example, Ibdah et al. reported that 79% of mothers with the Glu474Gln variant experienced liver disease during pregnancy [122]. Individuals carrying fetuses with LCHADD have a higher risk of developing AFLP, with one report that 49% of individuals with affected fetuses had AFLP and 11% had HELLP syndrome [123]. In an Austrian case series of 14 infants with LCHAD, 4 of the infants were born to mothers who had HELLP syndrome [81]. Ramanathan et al. suggested that mitochondrial dysfunction due to LCHAD deficiency in the fetus might lead to the accumulation of hepatotoxic long‐chain 3‐hydroxyacyl fatty acid intermediates in the maternal circulation, causing liver injury and contributing to the development of AFLP and HELLP syndrome [124].

Variations in the gene encoding for LCHAD (*HADHA, HADHB*) such as the common c.1528G>C (p.Glu474Gln) *HADHA* variants have been identified in affected children and are associated with maternal AFLP [125, 126]. In some populations, such as the Dutch, the carrier frequency of the common LCHAD variant is low, and heterozygosity for this variant does not seem to significantly increase the risk of HELLP syndrome, leading to the recommendation against routine screening of pregnant women for this mutation [127]. Other studies did not find a strong correlation between LC‐FAOD and HELLP syndrome. For example, heterozygosity for the common LCHAD variant was not found in a retrospective analysis of 88 Austrian infants from mothers with HELLP syndrome [128].

While there is evidence to suggest an association between fetal LCHAD deficiency and maternal liver diseases, including HELLP syndrome, the relationship is complex and not uniformly observed across all cases. Genetic factors play a role, but the exact mechanisms and risk factors remain unclear and warrant further investigation. Nevertheless, children born to mothers with HELLP/AFLP should be tested for LCHAD via NBS or diagnostic testing.

Recommendations:

- Do not screen for LCHAD in pregnant persons with HELLP/AFLP without additional risk factors or clinical indications.
  Quality of evidence: moderate.
  Strength of recommendation: strong.
- Monitor for development of HELLP syndrome and AFLP in pregnant individuals who are heterozygous for HADHA or HADHB pathogenic variants.
  Quality of evidence: moderate.
  Strength of recommendation: strong.

##### 3.2.14.2 Special Considerations for Pregnant Individuals With LC‐FAOD

Successful management of pregnant individuals with metabolic disorders, including VLCAD, TFP, and CPT2 deficiencies, has been reported using a multidisciplinary approach and tailored interventions to ensure adequate energy intake, particularly during periods of poor appetite, such as hyperemesis gravidarum, as well as during labor, delivery, and the postpartum period.

In published case studies and case series, patients with LC‐FAOD continued their pre‐pregnancy high‐carbohydrate, low‐fat diets with frequent meals and snacks [129, 130]. The biochemical profile and clinical symptoms often improve during the anabolic phase of pregnancy (the second and third trimesters) as the fetus aids in metabolizing fatty acids [131]. L‐carnitine is often provided if plasma concentrations are low. Additionally, supplements such as riboflavin and coenzyme Q10 have demonstrated subjective improvement in some patients [132].

While successful vaginal deliveries have been reported, Cesarean section delivery has been performed in some to mitigate the risk of complications associated with prolonged labor [129]. Pre‐delivery discussions with critical care teams ensure preparedness for potential complications [133]. During labor and delivery, intravenous glucose and hydration have been used to prevent rhabdomyolysis and potential renal complications [131]. In the post‐partum period, elevated CK associated with myalgia has been reported in several LC‐FAOD pregnancies, along with instances of post‐partum heart failure in patients with VLCADD [134].

Recommendations:

- For the nutritional management during pregnancy:
  ○ Ensure adequate energy intake with a high‐carbohydrate, low LCF diet (10%–25% of energy) to reduce reliance on body fat stores for energy during pregnancy.
  ○ Supplement with medium‐chain fat or triheptanoin to support energy metabolism.
  ○ Incorporate frequent meals and snacks, especially during periods of poor appetite and during labor.
    Quality of evidence: moderate.
    Strength of recommendation: strong.
- For management during labor and delivery: provide intravenous 10% dextrose in half normal saline at 1.35–1.5 times maintenance to prevent catabolism, rhabdomyolysis, and renal complications during labor and delivery and until the patient's normal diet resumes.
  Quality of evidence: moderate.
  Strength of recommendation: strong.
- For management post‐partum: Monitor blood CK level and clinical symptoms for early detection and management of complications such as rhabdomyolysis and acute heart failure.
  Quality of evidence: moderate.
  Strength of recommendation: strong.

### 3.3 Long‐Term Complications

#### 3.3.1 When do Neurologic Symptoms Occur? When Should Neurologic Examinations/Monitoring be Started, and at What Intervals? What Are the Necessary/Best Methods to Monitor Neurologic Manifestations?

LC‐FAOD can present at any age. There are case reports of antenatal presentation of LC‐FAOD [22, 135].

All forms of LC‐FAOD can present with neonatal encephalopathy associated with hypoglycemia, metabolic acidosis, deranged liver function, cardiomyopathy, and hyperammonemia. With prompt identification and treatment, many manifestations can be reversed, but long‐term neurocognitive sequelae can arise with severe metabolic decompensation at any age [81]. Later presentations of acute rhabdomyolysis can also occur with all forms of LC‐FAOD [136]. NBS can detect individuals with apparently abnormal LC‐FAOD biochemistry and genotype, but phenotypes are variable, with some being asymptomatic [137, 138].

Myopathy is well recognized in LC‐FAOD, with cases of LC‐FAOD identified after investigation of isolated elevated CK.

Peripheral neuropathy is a common finding only in patients with LCHAD/TFP deficiency, with a median time of onset being 5 years in TFPD and 15 years in LCHADD [139, 140, 141]. Isolated sensory, isolated motor, and combined sensorimotor neuropathy forms have been reported. Adult‐onset symptoms can mimic hereditary sensory motor neuropathy in TFP [142]. Neuropathy typically progresses, with acceleration during severe metabolic decompensation. Identification by NBS and subsequent early initiation of dietary treatment and emergency measures do not prevent the development of neuropathy [139, 141]. In some patients, there is a fulminant onset of neuropathy, mostly following specific triggers. In these cases, partial recovery is possible, especially with optimal metabolic and supportive management [143, 144]. More recently, a new phenotype of periodic paralysis in relation to febrile illness has been described in a child with a thermosensitive form of TFPD. Other presentations in patients with thermosensitive forms of TFPD are myopathic symptoms triggered by fever or exercise [145, 146].

Regular clinical examination, conducted during acute episodes, in recovery and at scheduled clinical visits, identifies the neurological status, progression, and potential recovery. Motor function is dramatically compromised with rhabdomyolysis in childhood but usually recovers. However, recurrent episodes can lead to chronic myopathy requiring appropriate metabolic treatment, physiotherapy, and regular exercise in order to restore baseline function [147].

In LCHAD/TFP deficiencies, the first sign of peripheral neuropathy is loss of the Achilles tendon reflex. This can progress to decreased sensation and impaired heel walking. Upper limbs are typically spared until later, but can be impacted, especially in TFPD. Nerve conduction abnormalities have been identified in several studies of LCHAD/TFP deficiencies. In the early phase, electrophysiological studies show decreased amplitude, indicative of distal axonal neuropathy, in the lower limb preserved. However, with age, progressive manifestations are observed, characterized by decreased amplitude and velocity in sensory and motor neurons. Electrophysiological manifestations can be observed before the onset of clinical neuropathy [139, 148].

One study used cross‐sectional analysis of muscle MRI to quantify abnormalities across a range of LC‐FAOD. T1W and STIR MRI sequences detected changes in the muscle of patients with LC‐FAOD, particularly during rhabdomyolysis [149]. The three patients with TFP deficiency all showed increased STIR signal in the lower leg muscles, whereas four of the 12 patients with VLCADD had increased signal in the upper leg muscles. Muscle MRI remains a research investigation.

Recommendations:

- Neurologic symptoms can occur at any age, depending on clinical severity, and should be regularly monitored through clinical examination in all LC‐FAOD patients. Periodic nerve conduction studies can be helpful in TFP and LCHAD deficiencies.
  Quality of evidence: low.
  Strength of recommendation: strong.
- If there are severe recurrent episodes of rhabdomyolysis or acute neuropathy, optimal metabolic management and supportive therapy including physiotherapy and exercise rehabilitation should be implemented.
  Quality of evidence: low.
  Strength of recommendation: strong.

#### 3.3.2 When do Cardiac Symptoms Usually Occur? Are They Associated With Metabolic Instability/Decompensations?

Cardiac manifestations such as hypertrophic and/or dilated cardiomyopathy, pericardial effusion, heart failure, arrhythmia, and sudden death are reported in LCHAD, TFP, neonatal CPT2, CACT, and VLCAD but not in CPT1 deficiencies. Despite NBS and early treatment, symptoms can still occur, especially during metabolic decompensation [64, 137, 150, 151, 152].

Severe cardiomyopathy has been seen in utero in TFP and CPT2 deficiencies [135]. However, cardiac manifestations most often appear in the neonatal period and in early childhood in all LC‐FAOD. In a few patients, cardiac complications begin later in life, usually associated with metabolic decompensation or with suboptimal treatment. Cardiac manifestations can appear independently of skeletal muscle involvement.

Severe variants and very low enzyme activity are more likely to result in in utero and neonatal cardiac presentation with poor outcome [150, 153]. In general, infections and catabolic conditions may provoke cardiac symptoms. The accumulation of arrhythmogenic intermediary metabolites of fatty acids, such as long‐chain acylcarnitines, may be responsible for arrhythmias [154]. Cardiomyopathy can be reversible with appropriate treatment [155].

Recommendation:

- Monitoring should be initiated after diagnosis. Frequency of monitoring depends on the severity of the disorder and symptoms. Monitor for cardiac complications, especially during metabolic decompensation.
  Quality of evidence: low.
  Strength of recommendation: fair.

#### 3.3.3 Is There Evidence for a LC‐FAOD‐Specific Treatment of Cardiac Symptoms/Cardiomyopathy (or Is the Cardiomyopathy Treated as Other Cardiomyopathies)?

Cardiomyopathy can be reversible on treatment with sufficient energy intake with a high intake of carbohydrates, LCF restriction, and supplementation of medium‐chain fat sources or triheptanoin [67, 68, 109, 110, 155]. Sodium‐D,L‐3‐hydroxybutyrate has been used to improve cardiac function in children with LC‐FAOD [156]. However, sodium overload is a risk with large doses. Alternatives include mixed ketone salts and ketone esters, evaluated in a small patient cohort [87]. Apart from dietary intervention, cardiomyopathy and arrhythmias are treated with non‐LC‐FAOD‐specific medication. Successful heart transplantation has been reported in TFP and CPT2 deficiencies [151, 157].

Severe ventricular arrhythmias, most commonly long QT syndrome, cause unexpected death and occur suddenly [103]. Apart from dietary management, no specific LC‐FAOD treatments are reported. The use of pacemaker and defibrillator devices has been suggested in patients who experience arrhythmia, but is unstudied.

Recommendation:

- In case of cardiac symptoms, optimal LC‐FAOD specific nutrition is key. Nutrition should be adapted to the age and clinical situation and consist of appropriate energy intake with a high energy percentage of carbohydrates, restriction of LCF, and supplementation with medium‐chain fat sources and/or ketones.
  Quality of evidence: low.
  Strength of recommendation: fair.

#### 3.3.4 What Is the Best Way to Diagnose and Monitor Cardiac Symptoms?

In most cases, echocardiography (left ventricular EF and wall mass), electrocardiography (ECG), and Holter are used to investigate cardiac symptoms [158]. Pre‐emptive prediction of acute arrhythmias has been difficult. Arrhythmias can occur independently of cardiomyopathy.

One study described the use of proton magnetic resonance imaging (MRI) and spectroscopy (1H‐MRS) in adults with LC‐FAOD. Subclinical morphological and functional differences between the hearts of patients with LC‐FAOD and matched controls were found, suggesting a chronic cardiac disease phenotype and hypertrophic LV remodeling in LC‐FAOD. This effect could be triggered by ongoing mild energy deficiency, rather than by lipotoxic effects of accumulating lipid metabolites [159]. Further research is needed to discern this process.

Recommendation:

- Regular monitoring by echocardiography (left ventricular EF and wall mass), electrocardiography (ECG), and continuous ECG monitoring (Holter) for cardiac symptoms is part of the follow‐up evaluation program of patients with VLCAD, CACT, early‐onset CPT2, and LCHAD/TFP deficiencies (see Section 3.1.1 for laboratory evaluations).
  Quality of evidence: low.
  Strength of recommendation: strong.

#### 3.3.5 At What Age do Ocular Symptoms Occur? When Should Ophthalmologic Examination be Started? At What Intervals?

Retinopathy is very common in LCHADD, uncommon in TFP deficiency, and not present in other LC‐FAOD [160, 161]. The fundus is reported to be normal at birth. Changes in the retina have been described in untreated patients from the age of 4 months, evolving from granular retinal pigment epithelium (“salt and pepper” scattering of pigment in retinas), with or without pigment clumping in the macula, to circumscribed chorioretinal atrophy, occlusion of choroidal vessels, and deterioration of central vision, often with relative sparing of the peripheral fundus. Subsequent posterior staphylomas and central scotomas can develop with progressive myopia, deterioration of visual fields and color vision, and finally severe loss of vision [160, 162, 163].

It is not yet clear which patients are at risk of developing ocular symptoms, and at what age these symptoms develop. The impact of NBS and treatment on ocular outcome is unclear.

Recommendation:

- In LCHADD and TFPD, ophthalmologic monitoring should be initiated at an early age. Frequency depends on symptom severity and potential interventions.
  Quality of evidence: low.
  Strength of recommendation: fair.

#### 3.3.6 What Are the Necessary/Best Methods to Monitor Ocular Manifestations?

The retinal changes in LCHAD/TFP deficiencies are described as a progressive rod‐cone dystrophy. Different methods have been reported to measure vision and the dystrophy: visual acuity, refraction, visual fields, ophthalmoscopy, fluorescein angiography, biometry, corneal topography, electroretinography (ERG), visual‐evoked potentials (VEP), color vision, dark adaptation, and color fundus photos. Images should demonstrate the optic nerve head, macula, retinal blood vessels, choroid, and the vitreous, and sub‐staging based on reference photographs in the follow‐up of LCHAD/TFPD retinopathy is advised in all. The images should be graded based on a standard scale published for this disease [160, 162, 163].

Recommendation:

- Periodic retinal imaging to document progression is recommended. Electroretinogram (ERG), visual acuity, visual evoked potentials (VEP), spectral domain optical coherence tomography (OCT) imaging, OCT angiography, and wide field fundus autofluorescence (FAF) imaging provide additional clinical information.
  Quality of evidence: low.
  Strength of recommendation: fair.

#### 3.3.7 Is There Any Evidence for Treatment With DHA to Prevent Retinopathy?

See also Section 3.2.7 One study demonstrated that retinal function as measured by ERG was inversely correlated with plasma concentrations of long‐chain 3‐hydroxyacylcarnitines. Early optimal dietary therapy, as indicated by low plasma 3‐hydroxyacylcarnitines and high plasma DHA concentrations, is associated with improved retention of retinal function and visual acuity in children with LCHAD or TFP deficiencies, but retinopathy appears to progress regardless [73, 80, 164].

Recommendation:

- DHA supplementation is advised if plasma concentrations are low. The effect of prophylaxis on retinopathy is not clear.
  Quality of evidence: low.
  Strength of recommendation: weak.

#### 3.3.8 What Is the Ideal Assessment Tool for the Quality of Life to LC‐FAOD Patients? What Are the Main Factors Influencing the QOL of LC‐FAOD Patients?

There is no validated quality‐of‐life assessment tool specifically for LC‐FAOD [165]. One study used the SF‐36 questionnaire to evaluate the efficacy of bezafibrate treatment in a small, open‐label cohort, but no comparison of QoL scores with a control population was reported [166]. Another looked at a range of parent coping mechanisms over the spectrum of IMD, including LC‐FAOD. It is likely that the majority of these LC‐FAOD were newborn‐screened patients and asymptomatic; the aggregate analysis across all IMDs demonstrated good coping mechanisms [167]. One study has examined thematic analysis across patients and carers with symptomatic LC‐FAOD. There were emerging themes that need to be validated on a broader spectrum of patients [168].

Several studies evaluated clinical outcomes, including fatalities (especially in CACT deficiency), episodes of hypoglycemia, metabolic decompensation, cardiomyopathy, rhabdomyolysis, and hospitalizations in general [64, 169]. QOL in most studies was impacted by a variety of symptoms. In most studies, clinical and social impacts were observed in severe LC‐FAOD. Further studies are required to determine whether the same impacts apply to attenuated forms of LC‐FAOD. A conceptual model has been proposed incorporating symptoms perceived in severe LC‐FAOD by caregivers and patients, including impacts on physical health, lifestyle, activities of daily living, psychological wellbeing, medical care, nutrition, and social integration, including school and work [168].

Similar to other IMD such as phenylketonuria and glycogen storage diseases, intensive dietary management is likely to impact social integration and self‐confidence. The morbidity associated with life‐threatening decompensations and neurological, ophthalmological, and cardiac dysfunction is very likely to have an impact on QOL. Physical and emotional support of patients through acute illness and recovery, dietary management, and social support is likely to improve confidence and QOL [170, 171, 172, 173].

Recommendation:

- LC‐FAOD‐specific outcome measures incorporating QOL need to be developed. Similar to other rare diseases, validated patient reported outcomes and lived experiences are required to document the patient journey and the effectiveness of interventions.
  Quality of evidence: low.
  Strength of recommendation: fair.

#### 3.3.9 Should Regular Exercise be Recommended? If Yes, What Kind of Physical Activity Should be Recommended (Endurance vs. Strength)?

Myopathy, characterized by recurrent episodes of rhabdomyolysis often provoked by physical exercise, is a frequent complication in LC‐FAOD. However, the use of exercise on long‐term outcomes and QOL has not been studied [85, 110]. Most research studies on exercise in LC‐FAOD have examined the effect of different substrates in short‐term exercise. Apart from two single‐case studies, exercise itself as an independent variable has not been comprehensively assessed [83, 174, 175, 176]. However, both in cases and in randomized crossover studies, the ability to exercise at moderate intensity for 40 min or longer has been demonstrated with either the use of carbohydrate, medium‐chain fat sources, triheptanoin or ketones 20–30 min prior to exercise [84, 85, 87, 110]. Individual exercise capacity appears to vary with the severity of the disorder and the participant's age.

Recommendation:

- Prescribe an exercise regimen for all patients. Individual exercise recommendations should be regularly adjusted based on the patient's exercise capacity, dietary management, age, and clinical symptoms.
  Quality of evidence: low.
  Strength of recommendation: strong.

#### 3.3.10 What Is the Best Way to Monitor Muscular Symptoms?

In several studies, CK, resting energy expenditure, steady‐state heart rate, glycolytic intermediates, and ketone bodies were measured during and/or following exercise [84, 85, 87, 110]. For other metabolic myopathies, such as McArdle syndrome, field testing, including wrist heart rate monitoring, has been used to monitor tachycardia associated with muscle symptoms [177]. There are no studies comparing different methods to monitor exercise effects and muscular symptoms. Multiple clinical reports identify pain and reduced mobility as prominent acute and chronic muscular symptoms. CK is typically measured in clinical practice; however, CK can be slightly elevated at baseline and rise later than symptoms during acute episodes.

Recommendation:

- Patients experiencing increased pain or decreased mobility should be evaluated clinically. Should acute symptoms arise, monitor CK. Renal output and function should be monitored during an episode, and in recovery from rhabdomyolysis.
  Quality of evidence: low.
  Strength of recommendation: strong.

## 4 Concluding Remarks

This first international guideline is the result of a 6‐year working process that utilized the modified GRADE methodology, combined with a Delphi vote among experts, to deliver the best available level of evidence for each recommendation. To summarize, these recommendations are based predominantly on low‐level evidence and primarily on expert consensus. This is mainly due to small case series and observational studies, rather than large‐scale randomized controlled trials, and the rarity and heterogeneity of the diseases. The natural history of LC‐FAOD is evolving in the era of NBS, with increased detection of phenotypically variable cases that complicate the interpretation of the true clinical course of these conditions. Together with evidence gaps, the diversity of regulatory systems governing the classification and reimbursement of dietary therapies, supplements, and pharmacological agents across countries has contributed to divergence in practice and disparities in access, underscoring the challenges inherent in producing global guidelines. Therefore, the recommendations contained herein should be considered as evolving and as a basis for further prospective clinical studies. At present, significant knowledge gaps remain regarding the optimal management of patients with LC‐FAOD. More and better‐quality evidence is needed, especially as the therapeutic landscapes evolve. Future multi‐center collaborative efforts and patient involvement will be critical to better characterize long‐term outcomes and inform evidence‐based management. Future integration of digital technologies and standardized data collection across centers will enable more efficient evidence aggregation, harmonized outcomes and patient care, and real‐time analysis of clinical practice patterns. This guideline should be updated regularly to enable health care professionals working in the field to make well‐informed decisions, ensuring the best evidence‐based care. An international approach is challenging, but for patients and caregivers worldwide, coordinated recommendations are essential for LC‐FAOD.

## Author Contributions

S.C.G., R.H.S., U.S.: conceptualization. K.B., S.C.G., D.K., M.L., F.R., G.V., J.V.: formal analysis. K.B., S.C.G., D.K., M.L., F.R., G.V., J.V.: investigation. S.C.G., R.H.S., J.V.: methodology. A.K., R.H.S.: project administration. R.H.S., U.S.: resources. K.B., S.C.G., D.K., M.L., F.R., G.V., J.V., International LC‐FAOD Guideline Workgroup: data curation. S.C.G., R.H.S.: supervision. K.B., S.C.G., D.K., M.L., F.R., G.V., J.V.: validation. K.B., S.C.G., D.K., M.L., F.R., R.H.S., G.V., J.V.: visualization. K.B., S.C.G., D.K., M.L., F.R., G.V., J.V.: writing – original draft preparation. K.B., S.C.G., D.K., M.L., F.R., R.H.S., U.S., G.V., J.V., International LC‐FAOD Guideline Workgroup: writing – review and editing.

## Funding

The guideline was supported by an SSIEM guideline grant, and by Emory University’s Medical Nutrition Therapy for Prevention (MNT4P) program.

## Disclosure

This guideline is formally endorsed by (1) European Reference Network for Rare Hereditary Metabolic Disorders (MetabERN); (2) International Network for Fatty Acid Oxidation Research and Management (INFORM).

## Ethics Statement

The Delphi survey data were collected under a protocol approved by the Emory University Institutional Review Board (IRB) (protocol #00009134).

## Conflicts of Interest

S.C.G. received honoraria from Vitaflo GmbH, Ultragenyx GmbH, Immedica Pharma GmbH, Danone GmbH, and Sprout GmbH; received support for meeting attendance from Nutricia Metabolics (Danone) GmbH; and participated on an Advisory Board for Ultragenyx GmbH. IA has received honoraria from PTC Therapeutics. D.K. received honoraria from Ultragenyx and Immedica; and received support for meeting attendance from INFORM. M.L. serves on the Dutch Advisory Board on Newborn Screening; and is a Council member for the Society for the Studies on Inborn Errors of Metabolism. F.R. is a managing director and board member of Met Ed Co. J.V. received research grants from Ultragenyx Pharmaceuticals and Reneo Pharmaceuticals; received consulting fees from Reneo Pharmaceuticals; received support for meeting attendance from Ultragenyx; participates on the Data Safety Monitoring Board at Jag Pharmaceuticals; is an editor for the North American Metabolic Academy; and is on the Board of Directors for the American College of Medical Genetics and Genomics. U.S. received grants and consulting fees from Ultragenyx; served as a consultant to the European Metabolic Group (Danone) and the Ultragenyx Advisory Board; and received support for meeting attendance from INFORM and the European Metabolic Group. The other authors declare no conflicts of interest.

## Supporting information


**File S1:** Literature search strategy.

**File S2:** Literature included in the review process.

## Data Availability

The data that support the findings of this study are available from the corresponding author upon reasonable request and subject to institutional data sharing protocols.

## Appendix A International LC‐FAOD Guideline Workgroup Members

Georgianne L. Arnold

University of Pittsburgh, Pittsburgh, Pennsylvania, USA

Kiera Batten

1. Department of Nutrition and Dietetics; and Genetic Metabolic Disorders Service, The Children's Hospital at Westmead, Sydney, Australia

2. School of Health Sciences, Faculty of Medicine and Health, UNSW Sydney, Australia

Terry G. J. Derks

1. Department of Metabolic Diseases, Beatrix Children's Hospital, University Medical Center Groningen, University of Groningen, Groningen, The Netherlands

2. UMCG Center of Expertise for Carbohydrate, Fatty Acid Oxidation and Ketone Bodies Disorders, University Medical Center Groningen, Groningen, The Netherlands

Patrick Forny

1. Department of Cell Biology and Physiology, Washington University School of Medicine, St. Louis, Missouri, USA

2. Division of Metabolism and Children's Research Center, University Children's Hospital Zurich, University of Zurich, Zurich, Switzerland

Melanie B. Gillingham

Department of Molecular and Medical Genetics, Graduate Programs in Human Nutrition, Oregon Health & Science University, Portland, Oregon, USA

Emma E. Glamuzina

Paediatric and Adult National Metabolic Service, Starship Children's Health, Te Whatu Ora Health New Zealand, Auckland, New Zealand

Anita Inwood

Queensland Children's Hospital, Queensland Australia and the University of Queensland, St Lucia, Queensland Australia

Monika Jörg‐Streller

Division of Nutrition and Dietetics, University Hospital Innsbruck, Innsbruck, Austria

Erle Kristensen

1. Department of Medical Biochemistry, Oslo University Hospital, Oslo, Norway

2. Department of Clinical Medicine, University of Bergen, Bergen, Norway

Svetlana Lajic

1. Department of Women's and Children's Health, Karolinska Institutet, Pediatric Endocrinology Unit, Karolinska University Hospital, SE‐171 76 Stockholm, Sweden

2. Department of Clinical Sciences, University of Gothenburg, Pediatric Endocrinology Unit, Sahlgrenska University Hospital, SE‐416 50 Gothenburg, Sweden

Alexander Laemmle

1. University Institute of Clinical Chemistry, Inselspital, Bern University Hospital, University of Bern, Bern, Switzerland

2. Division of Pediatric Endocrinology, Diabetology and Metabolism, Department of Pediatrics, Inselspital, University Hospital Bern, University of Bern, Switzerland

Risto Lapatto

Helsinki University Hospital and University of Helsinki, Helsinki, Finland

Elena Martín‐Hernández

Reference Center for Inherited Metabolic Disorders MetabERN, Hospital Research Institute (imas12), CIBERER, U723, “12 de Octubre” University Hospital, 28041 Madrid, Spain

Diego Martinelli

Inpatient Unit of Metabolic Diseases, Unit of Metabolic Diseases and Hepatology, Bambino Gesù Children's Research Hospital, Piazza Sant'Onofrio, 4 ‐ 00165 Rome, Italy

Mel McSweeney

Metabolic Medicine Department, Great Ormond Street Hospital, London, United Kingdom

Sonia Moreira

Internal Medicine Department, Hospitais da Universidade de Coimbra, Unidade Local de Saúde de Coimbra, Coimbra, Portugal

Margot F. Mulder

Department of Pediatrics, Division of Metabolic Diseases, Emma Children's Hospital, Amsterdam University Medical Center, 1105 AZ Amsterdam, The Netherlands

Anna Nordenström

Pediatric endocrinology and inborn errors of metabolism, Karolinska University Hospital, Department of Women's and Children's Health, Karolinska Institutet, S‐17176 Stockholm, Sweden

Pilar Quijada‐Fraile

Reference Center for Inherited Metabolic Disorders MetabERN, Hospital Research Institute (imas12), “12 de Octubre” University Hospital, 28041 Madrid, Spain

Bryony Ryder

Paediatric and Adult National Metabolic Service, Starship Children's Health, Te Whatu Ora Health New Zealand, Auckland, New Zealand

Marit Schwantje

Department of Metabolic Diseases, Wilhelmina Children's Hospital, University Medical Center Utrecht, Utrecht, the Netherlands

Ida Vanessa D. Schwartz

Hospital de Clínicas de Porto Alegre/Universidade Federal do Rio Grande do Sul, Brazil

Arthavan Selvanathan

1. Genetic Metabolic Disorders Service, Sydney Children's Hospital Network, Sydney, Australia

2. Disciplines of Genetic Medicine and Child and Adolescent Health, The University of Sydney, Sydney, Australia

Serap Sivri

Division of Pediatric Metabolism, Department of Pediatrics, Faculty of Medicine, Hacettepe University, Ankara, Türkiye

Maren Thiel

Patient advocate, Chairwoman of the German‐speaking support group for Inborn Errors of Fatty Acid Oxidation (Fett‐SOS e.V.), Berlin, Germany

Johan L.K. Van Hove

1. Department of Pediatrics, Section of Clinical Genetics and Metabolism, University of Colorado, Aurora, Colorado, USA

2. Department of Pathology and Laboratory Medicine, Children's Hospital Colorado, Aurora, Colorado, USA

Yılmaz Yıldız

Division of Pediatric Metabolism, Department of Pediatrics, Faculty of Medicine, Hacettepe University, Ankara, Türkiye

### Competing Interest Statement for Members of the Workgroup

K.B. received support for meeting attendance from Nutricia Danone, Vitaflo, and the Australasian Society of Inborn Errors of Metabolism. T.G.J.D. received grants from Ultragenyx Pharmaceutical Inc. Funding for fundamental research by The Nina Contreras D'Agosto Fund, and for the GSD Value project by Sophie's Hope Foundation and Global Center for GSD; received consulting fees from Ultragenyx Pharmaceutical Inc, ModernaTX Inc, and Beam Therapeutics; received payments or honoraria from MEDTalks, and Vitaflo; received support for meeting attendance from Danone; participated on a Data Safety Monitoring Board and Advisory Board for ModernaTX Inc; and is a Medical and Scientific Advisor for Sophie's Hope Foundation, and a Scientific Advisory Board member for Ketotic Hypoglycemia International, Glykogenose Deutschland e.V. and The GSD3 Foundation. M.B.G. received grant from Nestle Bioscience; received consulting fees from Travere Pharmaceutical; received payments or honoraria from Ultragenyx Pharmaceutical and Abbott; and received support for meeting attendance from INFORM. M.J.‐S. has received honoraria from LCHADD Preceptorship Salzburg and Vitaflo; and received support for meeting attendance from MetaX and Nutricia metabolics. A.L. received consulting fees for LC‐FAOD Advisory Board Meeting from Ultragenyx. D.M. received honoraria from Immedica, Mamoxi and Piam. E.M.‐H. received honoraria from Mead Johnson Nutrition; received support for meeting attendance from Nutricia Metabolics. M.M. received consulting fees from Glycomine; received honoraria and support to attend meetings from Immedica Pharma; is a member of British Inherited Metabolic Disease Group and the CDG UK advisory board. S.M. participates on the Advisory Board for Ultragenyx. A.N. received honoraria from the Swedish Board for Health and Welfare. P.Q.‐F. received honoraria from Vitaflo (Nestle) and Mead Johnson Nutrition. B.R. is a metabolic representative on the Royal Australasian College of Physicians’ Clinical Genetics Training Committee. I.V.D.S. received grant, honoraria and support for meeting attendance from Ultragenyx Pharmaceucal Inc. S.S. received honoraria from Biomarin; and received support for meeting attendance from Biomarin, PTC, Nutricia, and Nestle Healthcare. M.T. received honoraria from Nutricia and Ultragenyx; received support for meeting attendance from Ultragenyx; received dietary products and other services from Vitaflo, Dr. Schar/kanso and Nutricia metabolics; and is the chairwoman of the FAOD support group Fett‐SOS e.V. Y.Y. received honoraria from Nutricia Metabolics; received support for meeting attendance from Ultragenyx, Nutricia Metabolics, and Nestle Vitaflo; and is a local advisory board member for Nutricia Metabolics. The other authors do not declare any conflict of interest related to this work.
