# Supplementary material for: International Guideline on the Diagnosis, Treatment, and Monitoring of Long‐Chain Fatty Acid Oxidation Disorders (LC‐FAOD)
Source: J Inherit Metab Dis. 2026 Sep 24;49(5):e70251. doi: 10.1002/jimd.70251 (PMC13601914; doi:10.1002/jimd.70251)
Supplement: Supplementary file 1 [file JIMD-49-0-s001.docx]

**Methodology:**

The searches were originally designed for PubMed and were translated for Embase and Web of Science. To perform the search, a cassette of disease terms was grouped together and used in every search, see example below, and to this cassette the specified search terms for each question were added. In PubMed originally the Humans filter was used to filter out potential animal studies. In the follow-up search, it was decided that following search string: NOT ("animals"[MeSH Terms] NOT "humans"[MeSH Terms]) would be used instead of the Humans filter to allow additional articles in the search not indexed with MeSH terms. The “animals” MeSH terms were used to filter out studies conducted on model systems, such as mice, drosophila, or yeast. In addition, MeSH terms were used to filter out other meanings of the FAO or FAOD acronyms, such as Food and Agriculture Organization, fructosyl-amino acid oxidase, Fast Automatic Option Discovery, and Food Allergy outpatient department. The search results were exported from each database into EndNote. EndNote was used to de-duplicate the search results.

Thus, each research question had additional search terms used in conjunction with the disease search cassette:

((VLCAD OR Very-long-chain acyl-CoA dehydrogenase deficiency OR LCHAD OR “Long-chain L-3 hydroxyacyl-CoA” OR MTP OR Mitochondrial trifunctional protein dehydrogenase deficiency OR TFP OR Trifunctional protein deficiency OR CACT OR Carnitine-acylcarnitine translocase deficiency OR Carnitine palmitoyltransferase I deficiency OR CPT1 deficiency OR Carnitine palmitoyltransferase 2 deficiency OR CPT2 deficiency OR carnitine transporter deficiency OR OCTN2 OR “primary carnitine deficiency” OR “Long-chain fatty acid oxidation disorders” OR “FAOD”) AND ( Search Terms for each Individual Question) NOT ("animals"[MeSH Terms] NOT "humans"[MeSH Terms]))

**Exclusion terms:**

mouse/murine/mice

rat

bovine/cattle/cows

sheep

escherichia coli

cynopterus sphinx

aedes

caenorhabditis elegans/nematodes

porcine/piglets/sow

chicken/chicks

drosophila

takifugu

dogs

zebrafish

yeast

ctenopharyngodon

scotophilus

corynebacterium

equine/horses

bufo gargarizans

Food and Agriculture Organization

fructosyl-amino acid oxidase

Fast Automatic Option Discovery

Food Allergy outpatient department

**Search terms:**

Original 2020 search date: 1958 to Dec 31st 2020

Second search date range: Jan 1, 2021 through Nov 1, 2021

**Search terms for all key questions: "Fatty acid oxidation" and name of diseases (in different spelling/abbreviation)**

**Included diseases:**

VLCAD

LCHAD

MTP or TFP

CACT

CPT 1

CPT 2

carnitine transporter deficiency or OCTN2 or primary carnitine deficiency

**Working Group 1: Treatment**

**1.1.1 For the long-term nutrition management of asymptomatic individuals with LCFAOD, what is the recommended diet to promote optimal nutrition and health outcomes?**

**o   What fasting intervals are safe (age groups: infants, children, adolescents, adults)?	fasting	cornstarch	precautions**

**o   Who can be breastfed/ partially breastfed?**

**breastfeeding	infant formula**

**o   What is the recommended diet, including total fat, long-chain fat and medium chain fat, for different age groups and different FAODS?**

**energy	calories	total fat	long chain fat	MCT	carbohydrates	protein	cornstarch	glucose polymers	medical food	formula**

**1.2.1 For the long-term nutrition management of symptomatic individuals with LCFAOD, what is the recommended diet to promote optimal nutrition and health outcomes?**

**o   What fasting intervals are safe (age groups: infants, children, adolescents, adults)?	fasting	cornstarch**

**o   Who can be breastfed/ partially breastfed?**

**breastfeeding	infant formula**

**o   What is the recommended diet, including total fat, long-chain fat and medium chain fat, for different age groups and different FAODS?**

**energy	calories	total fat	long chain fat	MCT	carbohydrates	protein	cornstarch	glucose polymers	medical food	formula**

**1.3 Indications for tube-feeding**

**growth failure	tube feeding	enteral feeds	gastrostomy	parenteral nutrition**

**1.4 What dietary measures are recommended before physical exercise?	carbohydrate	MCT intake	MCT supplementation	glucose polymers	hydration	fluids	precautions**

**1.5 Is there evidence for treatment with DHA in FAODs?**

**Essential Fatty Acids	Arachodonic acid (ARA)	Docosohexaenoic acid (DHA)	Eicosapentaenoic acid (EPA)	Essential fatty acid supplementation	Linoleic acid	alpha-linoleinc acid**

**1.6 What is recommended for monitoring of nutrition status?**

**Nutrient analysis, , fat soluble vitamins-d,25, acylcarnitine profile, creatine kinase, CKMB, BNP, total carnitine, free carnitine, carnitine esters, blood glucose levels, ECHO, EKG, holter monitoring, troponin levels	Anthropometrics	Laboratory values	liver function tests-AST/ALT	essential fatty acid profiles	complete blood count	iron	ferritin	fat-soluble vitamins	D-25	acylcarnitine profile	creatine kinase	CKMB	BNP	total carinitne	free carnitine	carnitine esters	blood glucose	ECHO	EKG	holter monitoring	troponin levels**

**1.7 -  Is there evidence for treatment with L-carnitine in FAODs?**

**carnitine supplementation	carnitine	L-carnitine	and treatment or therapy**

**1.8 - Is there evidence for treatment with C7 in FAODs?**

**triheptanoin	heptanoate	C7 oil	doljovi	and treatment or therapy**

**1.9 - What are the criteria for hospital admission?**

**metabolic decompensation	hospital admission	hospitalization	hospital	metabolic crisis**

**1.10 - Sick day protocol for intercurrent illnesses at home (When to start and which regimen?)**

**emergency protocol	unwell regimen	treatment protocol	acute management	nutrition support	parenteral nutrition	treatment	therapy**

**1.11 - What is the recommended nutritional and medical management during hospitalization?**

**emergency protocol	unwell regimen	treatment protocol	acute management	nutrition support	parenteral nutrition	treatment	therapy**

**1.12 - What special treatment recommendations exist for pregnant patients?**

**pregnancy	post-partum	HELLP sydrome	fatty liver of pregnancy	labor and delivery**

**Working Group 2: Diagnosis**

**What is the preferable method for primary confirmation diagnostics after positive NBS (reliable and rapid disease confirmation)? (ranking, according to availability)	confirmation diagnostics	enzyme analysis	genetic/ molecular analysis	biochemical work-up/ biochemical markers	flux analysis	newborn screening**

**What are the best tools for severity assessment?**

**genotype-phenotype correlation	severity assessment	enzyme testing/ studies / analysis	flux analysis	prognosis**

**What methods are available and recommended for prenatal diagnostics?**

**prenatal diagnostics**

**What clinical and laboratory work-up has to be performed?**

**laboratory work-up	clinical examination	o    At time of positive NBS in asymptomatic cases**

**laboratory work-up	clinical examination	asymptomatic	newborn screening**

- **After confirmation of the disease in asymptomatic cases**

**laboratory work-up	clinical examination	asymptomatic	newborn screening**

**o In symptomatic cases**

**laboratory work-up	clinical examination	symptomatic**

**What is the role of next generation sequencing in the diagnostics of FAODs?**

**next generation sequencing	whole exome sequencing	diagnostics**

**Working Group 3: Monitoring and long-term complications**

**Quality of life**

**o    What is the ideal assessment tool for the quality of life to FAOD patients?	Quality of life	disease burden	assessment**

**o   What are the main factors influencing the QoL of FAOD patients?**

**Quality of life	disease burden	assessment**

**Cardiac manifestations (e.g. cardiomyopathy, rhythm disorders, pericardial effusion)**

**o    When do cardiac symptoms usually occur? Are they associated with metabolic instability/decompensations?**

**cardiomyopathy	heart rhythm disorders	arrhythmia	cardiologic diagnostics	pericardial effusion**

**o    Is there evidence for a FAOD-specific treatment of cardiac symptoms/cardiomyopathy (or is the cardiomyopathy treated as other cardiomyopathies)?**

**cardiomyopathy	heart rhythm disorders	arrhythmia	treatment	pericardial effusion**

**o    What is the best way to diagnose and monitor cardiac symptoms?	cardiomyopathy	heart rhythm disorders	arrhythmia	monitoring	diagnostics	echocardiography	MRI**

**Myopathy/Muscular weakness/Exercise intolerance**

**o    What is the best way to monitor muscular symptoms?**

**myopathy	muscle weakness	exercise intolerance	monitoring	assessment	rhabdomyolysis	muscle pain**

**o    Should regular exercise be recommended? If yes, what kind of physical activity should be recommended (endurance versus strength)?**

**physical activity	sports	exercise**

**Ocular manifestations (e.g. pigmentary retinopathy)**

**o    When (age) do ocular symptoms occur?**

**retinopathy	onset	eye symptoms	ocular symptoms	ophthalmologic symptoms**

**o    When should ophthalmologic examination be started? At what intervals?	retinopathy	monitoring	ophthalmologic examination	interval	manifestation	onset**

**o    What are the necessary/best methods to monitor ocular manifestations?	retinopathy	monitoring	assessment/evaluation	diagnostics**

**o    Is there any evidence for treatment with DHA? (see also working group 1)	retinopathy	Docosahexaenoic acid (DHA)**

**Neurological manifestations (e.g. peripheral neuropathy)/ Neurocognitive status	neuropathy	Neurocognitive status/development	apnea**

**ventilation**

**o    When do neurological symptoms occur?**

**neuropathy	manifestation	onset**

**o    When should neurologic examinations/ monitoring be started? At what intervals?**

**neuropathy	monitoring	assessment	onset	examination	nerve conduction velocity	somatosensory potential**

**o    What are the necessary/best methods to monitor neurologic manifestations?	neuropathy	monitoring	assessment	neurocognitive testing/ IQ testing	neurography	electrophysiology	nerve conduction velocity	somatosensory potential**

**Growth and nutritional monitoring**

**o    Which laboratory parameters have to be assessed to monitor nutritional status?	nutritional status/ nutrition status	growth	laboratory monitoring	nutritional deficiency	essential fatty acids	vitamins**

**o    Indications for nutritional protocols?**

**nutritional status/ nutrition status	growth	dietary/nutrition protocols	calculation**
