# Supplementary material for: International Guideline on the Diagnosis, Treatment, and Monitoring of Long‐Chain Fatty Acid Oxidation Disorders (LC‐FAOD)
Source: J Inherit Metab Dis. 2026 Sep 24;49(5):e70251. doi: 10.1002/jimd.70251 (PMC13601914; doi:10.1002/jimd.70251)
Supplement: Supplementary file 2 [file JIMD-49-0-s002.docx]

**Literature included in the review process**

Abdenur, J. E., Lavorgna, S., Schenone, A. B., Guinle, A., Jorge, L., Andresen, B., Gregersen, N., & Chamoles, N. (2004). Complex carbohydrates in the treatment of LCHAD deficiency. Annual Meeting of the Society for Inherited Metabolic Disorders.

Adeva-Andany, M. M., Carneiro-Freire, N., Seco-Filgueira, M., Fernández-Fernández, C., & Mouriño-Bayolo, D. (2019). Mitochondrial β-oxidation of saturated fatty acids in humans. Mitochondrion, 46, 73–90. https://doi.org/10.1016/J.MITO.2018.02.009

Adhikari, A. N., Gallagher, R. C., Wang, Y., Currier, R. J., Amatuni, G., Bassaganyas, L., Chen, F., Kundu, K., Kvale, M., Mooney, S. D., Nussbaum, R. L., Randi, S. S., Sanford, J., Shieh, J. T., Srinivasan, R., Sunderam, U., Tang, H., Vaka, D., Zou, Y., … Brenner, S. E. (2020). The role of exome sequencing in newborn screening for inborn errors of metabolism. Nature Medicine, 26(9), 1392–1397. https://doi.org/10.1038/s41591-020-0966-5

Akar, H. T., Çağan, M., Yıldız, Y., & Sivri, H. S. (2021). Complicated peripartum course in a patient with very long-chain acyl-coenzyme A dehydrogenase (VLCAD) deficiency. Neuromuscular Disorders, 31(6), 566–569. https://doi.org/10.1016/J.NMD.2021.03.015

Alaei, F., Shakiba, M., Saneifard, H., Vahidshahi, K., & Alaei, M. (2022). Defects in Very Long-Chain Fatty Acid Oxidation Presenting as Different Types of Cardiomyopathy. Case Reports in Cardiology, 2022, 1–4. https://doi.org/10.1155/2022/5529355

Al-Amrani, F., Ruiter, J. P. N., Doolaard, M., Kumar, A., Ferdinandusse, S., & Al-Thihli, K. (2025). Periodic Paralysis in a Child With Thermosensitive Mitochondrial Trifunctional Protein Deficiency. American Journal of Medical Genetics. Part A, 197(2), e63900. https://doi.org/10.1002/ajmg.a.63900

Alaygut, D., Bayram, M. T., Kasap, B., Soylu, A., Türkmen, M., & Kavukcu, S. (2017). Rhabdomyolysis with different etiologies in childhood. World Journal of Clinical Pediatrics, 6(4), 161–168. https://doi.org/10.5409/WJCP.V6.I4.161

Alfares, A., Alfadhel, M., Mujamammi, A., Alotaibi, B., Albahkali, S., Al Balwi, M., Benabdelkamel, H., Masood, A., Ali, R., Almuaysib, A., Al Mahri, S., Mohammad, S., Alanazi, I. O., Alfadda, A., AlGhamdi, S., & Alrfaei, B. M. (2020). Proteomic and Molecular Assessment of the Common Saudi Variant in ACADVL Gene Through Mesenchymal Stem Cells. Frontiers in Cell and Developmental Biology, 7. https://doi.org/10.3389/FCELL.2019.00365

Alghamdi, A., Almalki, H., Shawli, A., Waggass, R., & Hakami, F. (2018). A case of atypical systemic primary carnitine deficiency in Saudi Arabia. Pediatric Reports, 10(2), 39–42. https://doi.org/10.4081/PR.2018.7705

Alhashem, A., Mohamed, S., Abdelraheem, M., AlGufaydi, B., & Al-Aqeel, A. (2020). Molecular and clinical characteristics of very long-chain acyl-CoA dehydrogenase deficiency. Saudi Medical Journal, 41(6), 590–596. https://doi.org/10.15537/smj.2020.6.25131

Al-Hertani, W., Cordeiro, D., Burr, L., Olpin, S., & Raiman, J. (2010). A puzzling case of carnitine-acylcarnitine translocase (CACT) deficiency, diagnosed on newborn screening. Journal of Inherited Metabolic Disease, 33, S58. https://doi.org/10.1007/s10545-010-9163-x

Almannai, M., Alfadhel, M., & El-Hattab, A. W. (2019). Carnitine inborn errors of metabolism. Molecules, 24(18). https://doi.org/10.3390/MOLECULES24183251

Al-Sannaa, N. A., & Cheriyan, G. M. (2010). Carnitine-acylcarnitine translocase deficiency. Clinical course of three Saudi children with a severe phenotype. Saudi Med J, 31(8), 931–934.

Al-Thihli, K., Sinclair, G., Sirrs, S., Mezei, M., Nelson, J., & Vallance, H. (2014). Performance of serum and dried blood spot acylcarnitine profiles for detection of fatty acid β-oxidation disorders in adult patients with rhabdomyolysis. Journal of Inherited Metabolic Disease, 37(2), 207–213. https://doi.org/10.1007/S10545-012-9578-7

Ambrose, A., Sheehan, M., Bahl, S., Athey, T., Ghai-Jain, S., Chan, A., & Mercimek-Andrews, S. (2022). Outcomes of mitochondrial long chain fatty acid oxidation and carnitine defects from a single center metabolic genetics clinic. Orphanet Journal of Rare Diseases, 17(1). https://doi.org/10.1186/S13023-022-02512-5

Anderson, S., & Brooks, S. S. (2013). When the usual symptoms become an unusual diagnosis: A case report of trifunctional protein complex. Neonatal Network, 32(4), 262–273. https://doi.org/10.1891/0730-0832.32.4.262

Andresen, B. S., Olpin, S., Kvittingen, E. A., Augoustides-Savvopoulou, P., Lindhout, D., Halley, D. J. J., Vianey-Saban, C., Wanders, R. J. A., Ijlst, L., Schroeder, L. D., Bolund, L., & Gregersen, N. (1999). DNA-based prenatal diagnosis for very-long-chain acyl-CoA dehydrogenase deficiency. Journal of Inherited Metabolic Disease, 22(3), 281–285. https://doi.org/10.1023/A:1005558828223

Andresen, B. S., Olpin, S., Poorthuis, B. J. H. M., Scholte, H. R., Vianey-Saban, C., Wanders, R., Ijlst, L., Morris, A., Pourfarzam, M., Bartlett, K., Baumgartner, E. R., DeKlerk, J. B. C., Schroeder, L. D., Corydon, T. J., Lund, H., Winter, V., Bross, P., Bolund, L., & Gregersen, N. (1999). Clear correlation of genotype with disease phenotype in very-long-chain acyl-CoA dehydrogenase deficiency. American Journal of Human Genetics, 64(2), 479–494. https://doi.org/10.1086/302261

Androudi, S., Mataftsi, A., & Brazitikos, P. (2018). Retinal Dystrophy in Long-Chain 3-hydroxyacyl-CoA Dehydrogenase Deficiency. Ophthalmology Retina, 2(1), 74. https://doi.org/10.1016/J.ORET.2017.09.012

Angelini, C., Federico, A., Reichmann, H., Lombes, A., Chinnery, P., & Turnbull, D. (2006). Task force guidelines handbook: EFNS guidelines on diagnosis and management of fatty acid mitochondrial disorders. European Journal of Neurology, 13(9), 923–929. https://doi.org/10.1111/J.1468-1331.2006.01482.X

Angelini, C., Nascimbeni, A. C., & Semplicini, C. (2013). Therapeutic advances in the management of Pompe disease and other metabolic myopathies. Therapeutic Advances in Neurological Disorders, 6(5), 311–321. https://doi.org/10.1177/1756285613487570

Anichini, A., Fanin, M., Vianey-Saban, C., Cassandrini, D., Fiorillo, C., Bruno, C., & Angelini, C. (2011). Genotype - Phenotype correlations in a large series of patients with muscle type CPT II deficiency. Neurological Research, 33(1), 24–32. https://doi.org/10.1179/016164110X12767786356390

Antunes, A. P., Nogueira, C., Rocha, H., Vilarinho, L., & Evangelista, T. (2013). Intermittent rhabdomyolysis with adult onset associated with a mutation in the ACADVL Gene. Journal of Clinical Neuromuscular Disease, 15(2), 69–72. https://doi.org/10.1097/CND.0000000000000012

Arnold, G. L., Van Hove, J., Freedenberg, D., Strauss, A., Longo, N., Burton, B., Garganta, C., Ficicioglu, C., Cederbaum, S., Harding, C., Boles, R. G., Matern, D., Chakraborty, P., & Feigenbaum, A. (2009). A Delphi clinical practice protocol for the management of very long chain acyl-CoA dehydrogenase deficiency. Molecular Genetics and Metabolism, 96(3), 85–90. https://doi.org/10.1016/J.YMGME.2008.09.008

Arnold, G. L., Yester, J., McCracken, E., Feingold, B. D., & Vockley, J. (2021). Successful orthotopic heart transplantation in CPTII deficiency. Molecular Genetics and Metabolism, 133(2), 182–184. https://doi.org/10.1016/J.YMGME.2021.04.005

Arunath, V., Liyanarachchi, M. S., Gajealan, S., Jasinge, E., Weerasekara, K., & Moheb, L. A. (2021). A novel mutation in ACADVL causing very long-chain acyl-coenzyme-A dehydrogenase deficiency in a South Asian pediatric patient: a case report and review of the literature. Journal of Medical Case Reports, 15(1). https://doi.org/10.1186/S13256-021-03013-Y

Atkins, A. E., Tarini, B. A., Phillips, E. K., & Calhoun, A. R. U. L. (2019). Misclassification of VLCAD carriers due to variable confirmatory testing after a positive NBS result. Journal of Community Genetics, 10(4), 447–451. https://doi.org/10.1007/S12687-019-00409-8

Autti‐Rämö, I., Måkelå, M., Sintonen, H., Koskinen, H., Laajalahti, L., Halila, R., Kååriåinen, H., Lapatto, R., Nåntö‐salonen, K., Pulkki, K., Renlund, M., Salo, M., & Tyni, T. (2005). Expanding screening for rare metabolic disease in the newborn: An analysis of costs, effect and ethical consequences for decision‐making in Finland. Acta Paediatrica, 94(8), 1126–1136. https://doi.org/10.1111/J.1651-2227.2005.TB02056.X

Avila-Smirnow, D., Boutron, A., Beytía-Reyes, M. D. L. Á., Contreras-Olea, O., Caicedo-Feijoo, A., Gejman-Enríquez, R., Escobar-Henríquez, R., & Förster-Mujica, J. (2018). Carnitine palmitoyltransferase type 2 deficiency: Novel mutation in a Native South American family with whole-body muscle magnetic resonance imaging findings: Two case reports. Journal of Medical Case Reports, 12(1). https://doi.org/10.1186/S13256-018-1702-3

Baker, J. J., & Burton, B. K. (2021). Diagnosis and Clinical Management of Long-chain Fatty-acid Oxidation Disorders: A Review. TouchREVIEWS in Endocrinology, 17(2), 108. https://doi.org/10.17925/EE.2021.17.2.108

Balci, M. C., Karaca, M., Ergul, Y., Omeroglu, R. E., Demirkol, M., & Gokcay, G. F. (2022). Cardiologic evaluation of Turkish mitochondrial fatty acid oxidation disorders. Pediatrics International, 64(1). https://doi.org/10.1111/PED.15317

Baruteau, J., Sachs, P., Broué, P., Brivet, M., Abdoul, H., Vianey-Saban, C., & Ogier De Baulny, H. (2013). Clinical and biological features at diagnosis in mitochondrial fatty acid beta-oxidation defects: A French pediatric study of 187 patients. Journal of Inherited Metabolic Disease, 36(5), 795–803. https://doi.org/10.1007/S10545-012-9542-6

Baskin, B., Geraghty, M., & Ray, P. N. (2010). Paternal isodisomy of chromosome 2 as a cause of long chain 3-hydroxyacyl-CoA dehydrogenase (LCHAD) deficiency. American Journal of Medical Genetics, Part A, 152(7), 1808–1811. <https://doi.org/10.1002/AJMG.A.33462>

Batten, K., Bhattacharya, K., Simar, D., & Broderick, C. (2023) Exercise testing and prescription in patients with inborn errors of muscle energy metabolism. *J Inherit Metab Dis,* 46, 763-777. doi: 10.1002/jimd.12644.

Behrend, A. M., Harding, C. O., Shoemaker, J. D., Matern, D., Sahn, D. J., Elliot, D. L., & Gillingham, M. B. (2012). Substrate oxidation and cardiac performance during exercise in disorders of long chain fatty acid oxidation. Molecular Genetics and Metabolism, 105(1), 110–115. https://doi.org/10.1016/J.YMGME.2011.09.030

Bennett, M. J., Rinaldo, P., & Strauss, A. W. (2000). Inborn errors of mitochondrial fatty acid oxidation. Critical Reviews in Clinical Laboratory Sciences, 37(1), 1–44. https://doi.org/10.1080/10408360091174169

Berardinelli, A., & D’Antona, G. (2019). Physiological aspects of muscular adaptations to training translated to neuromuscular diseases. Acta Myologica, 38(4), 197–206.

Bertini, E., Dionisi-Vici, C., Garavaglia, B., Burlina, A. B., Sabatelli, M., Rimoldi, M., Bartuli, A., Sabetta, G., & DiDonato, S. (1992). Peripheral sensory-motor polyneuropathy, pigmentary retinopathy, and fatal cardiomyopathy in long-chain 3-hydroxy-acyl-CoA dehydrogenase deficiency. European Journal of Pediatrics, 151(2), 121–126. https://doi.org/10.1007/BF01958956

Bhattacharya, K., Matar, W., Tolun, A. A., Devanapalli, B., Thompson, S., Dalkeith, T., Lichkus, K., & Tchan, M. (2020). The use of sodium DL-3-Hydroxybutyrate in severe acute neuro-metabolic compromise in patients with inherited ketone body synthetic disorders. Orphanet Journal of Rare Diseases, 15(1), 53. https://doi.org/10.1186/s13023-020-1316-x

Bialer, M. G., Nada, M. A., Vianeysaban, C., Roe, C. R., Mathieu, M., McGlynn, J. A., Ding, J. H., Mandon, G., & Slonim, A. E. (1995). Prenatal-Diagnosis of very long chain acyl-CoA dehydrogenase (VLCAD) deficiency. American Society of Human Genetics 45th Annual Meeting.

Bleeker, J. C., Kok, I. L., Ferdinandusse, S., de Vries, M., Derks, T. G. J., Mulder, M. F., Williams, M., Gozalbo, E. R., Bosch, A. M., van den Hurk, D. T., de Sain-van der Velden, M. G. M., Waterham, H. R., Wijburg, F. A., & Visser, G. (2019). Proposal for an individualized dietary strategy in patients with very long-chain acyl-CoA dehydrogenase deficiency. Journal of Inherited Metabolic Disease, 42(1), 159–168. https://doi.org/10.1002/JIMD.12037

Bleeker, J. C., Kok, I. L., Ferdinandusse, S., van der Pol, W. L., Cuppen, I., Bosch, A. M., Langeveld, M., Derks, T. G. J., Williams, M., de Vries, M., Mulder, M. F., Gozalbo, E. R., de Sain-van der Velden, M. G. M., Rennings, A. J., Schielen, P. J. C. I., Dekkers, E., Houtkooper, R. H., Waterham, H. R., Pras-Raves, M. L., … Visser, G. (2019). Impact of newborn screening for very-long-chain acyl-CoA dehydrogenase deficiency on genetic, enzymatic, and clinical outcomes. Journal of Inherited Metabolic Disease, 42(3), 414–423. https://doi.org/10.1002/JIMD.12075

Bleeker, J. C., Visser, G., Clarke, K., Ferdinandusse, S., de Haan, F. H., Houtkooper, R. H., IJlst, L., Kok, I. L., Langeveld, M., van der Pol, W. L., de Sain-van der Velden, M. G. M., Sibeijn-Kuiper, A., Takken, T., Wanders, R. J. A., van Weeghel, M., Wijburg, F. A., van der Woude, L. H., Wüst, R. C. I., Cox, P. J., & Jeneson, J. A. L. (2020). Nutritional ketosis improves exercise metabolism in patients with very long-chain acyl-CoA dehydrogenase deficiency. Journal of Inherited Metabolic Disease, 43(4), 787–799. https://doi.org/10.1002/JIMD.12217

Bo, R., Awano, H., Yamada, K., Ooi, M., Okata, Y., Bitoh, Y., Mizobuchi, S., & Iijima, K. (2021). The perioperative transition of serum biomarkers of a 1.5-year-old boy with very-long-chain acyl-CoA dehydrogenase deficiency. Molecular Genetics and Metabolism Reports, 27. https://doi.org/10.1016/J.YMGMR.2021.100760

Bo, R., Hasegawa, Y., Yamada, K., Kobayashi, H., Taketani, T., Fukuda, S., & Yamaguchi, S. (2016). A fetus with mitochondrial trifunctional protein deficiency: Elevation of 3-OH-acylcarnitines in amniotic fluid functionally assured the genetic diagnosis. Molecular Genetics and Metabolism Reports, 6, 1–4. https://doi.org/10.1016/J.YMGMR.2015.11.005

Bo, R., Musha, I., Yamada, K., Kobayashi, H., Hasegawa, Y., Awano, H., Arao, M., Kikuchi, T., Taketani, T., Ohtake, A., Yamaguchi, S., & Iijima, K. (2020). Need for strict clinical management of patients with carnitine palmitoyltransferase II deficiency: Experience with two cases detected by expanded newborn screening. Molecular Genetics and Metabolism Reports, 24. https://doi.org/10.1016/J.YMGMR.2020.100611

Bo, R., Yamada, K., Kobayashi, H., Jamiyan, P., Hasegawa, Y., Taketani, T., Fukuda, S., Hata, I., Niida, Y., Shigematsu, Y., Iijima, K., & Yamaguchi, S. (2017). Clinical and molecular investigation of 14 Japanese patients with complete TFP deficiency: A comparison with Caucasian cases. Journal of Human Genetics, 62(9), 809–814. https://doi.org/10.1038/JHG.2017.52

Boemer, F., Deberg, M., Schoos, R., Caberg, J. H., Gaillez, S., Dugauquier, C., Delbecque, K., François, A., Maton, P., Demonceau, N., Senterre, G., Ferdinandusse, S., & Debray, F. G. (2016). Diagnostic pitfall in antenatal manifestations of CPT II deficiency. Clinical Genetics, 89(2), 193–197. https://doi.org/10.1111/CGE.12593

Boese, E. A., Jain, N., Jia, Y., Schlechter, C. L., Harding, C. O., Gao, S. S., Patel, R. C., Huang, D., Weleber, R. G., Gillingham, M. B., & Pennesi, M. E. (2016). Characterization of Chorioretinopathy Associated with Mitochondrial Trifunctional Protein Disorders: Long-Term Follow-up of 21 Cases. Ophthalmology, 123(10), 2183–2195. https://doi.org/10.1016/J.OPHTHA.2016.06.048

Boles, R. G. (2002). Very long‐chain acyl‐CoA dehydrogenase deficiency in an infant presenting with massive hepatomegaly. Journal of Inherited Metabolic Disease, 25(4), 315–316. https://doi.org/10.1023/A:1016554409214

Boneh, A., Andresen, B. S., Gregersen, N., Ibrahim, M., Tzanakos, N., Peters, H., Yaplito-Lee, J., & Pitt, J. J. (2006). VLCAD deficiency: Pitfalls in newborn screening and confirmation of diagnosis by mutation analysis. Molecular Genetics and Metabolism, 88(2), 166–170. https://doi.org/10.1016/J.YMGME.2005.12.012

Bonnefont, J. P., Demaugre, F., Prip-Buus, C., Saudubray, J. M., Brivet, M., Abadi, N., & Thuillier, L. (1999). Carnitine palmitoyltransferase deficiencies. Molecular Genetics and Metabolism, 68(4), 424–440. https://doi.org/10.1006/MGME.1999.2938

Bonnet, D., Martin, D., De Lonlay, P., Villain, E., Jouvet, P., Rabier, D., Brivet, M., & Saudubray, J. M. (1999). Arrhythmias and conduction defects as presenting symptoms of fatty acid oxidation disorders in children. Circulation, 100(22), 2248–2253. https://doi.org/10.1161/01.CIR.100.22.2248

Boonsimma, P., Crosby, K., Mohan, P., Puscasiu, E., & Tanpaiboon, P. (2021). A patient with atypical presentation of chronic hepatosteatosis harboring a novel variant in the CPT1A gene. European Journal of Medical Genetics, 64(1). https://doi.org/10.1016/J.EJMG.2020.104034

Borch, L., Lund, A. M., Wibrand, F., Christensen, E., Søndergaard, C., Gahrn, B., Hougaard, D. M., Andresen, B. S., Gregersen, N., & Olsen, R. K. J. (2012). Normal levels of plasma free carnitine and acylcarnitines in follow-up samples from a presymptomatic case of carnitine palmitoyl transferase 1 (CPT1) deficiency detected through newborn screening in Denmark. JIMD Reports, 3, 11–15. https://doi.org/10.1007/8904_2011_35

Bower, A., Imbard, A., Benoist, J. F., Pichard, S., Rigal, O., Baud, O., & Schiff, M. (2019). Diagnostic contribution of metabolic workup for neonatal inherited metabolic disorders in the absence of expanded newborn screening. Scientific Reports, 9(1). https://doi.org/10.1038/S41598-019-50518-0

Brivet, M., Boutron, A., Slama, A., Costa, C., Thuillier, L., Demaugre, F., Rabier, D., Saudubray, J. M., & Bonnefont, J. P. (1999). Defects in activation and transport of fatty acids. Journal of Inherited Metabolic Disease, 22(4), 428–441. https://doi.org/10.1023/A:1005552106301

Brown, A., Crowe, L., Boneh, A., & Anderson, V. (2017). Parent coping and the behavioural and social outcomes of children diagnosed with inherited metabolic disorders. JIMD Reports, 31, 29–36. https://doi.org/10.1007/8904_2016_544

Brown-Harrison, M. C., Nada, M. A., Sprecher, H., Vianey-Saban, C., Farquhar, J., Gilladoga, A. C., & Roe, C. R. (1996). Very long chain acyl-CoA dehydrogenase deficiency: Successful treatment of acute cardiomyopathy. Biochemical and Molecular Medicine, 58(1), 59–65. https://doi.org/10.1006/BMME.1996.0033

Browning, M. F., Larson, C., Strauss, A., & Marsden, D. L. (2005). Normal acylcarnitine levels during confirmation of abnormal newborn screening in long-chain fatty acid oxidation defects. Journal of Inherited Metabolic Disease, 28(4), 545–550. https://doi.org/10.1007/S10545-005-0545-4

Bursle, C., Weintraub, R., Ward, C., Justo, R., Cardinal, J., & Coman, D. (2018). Mitochondrial trifunctional protein deficiency: Severe cardiomyopathy and cardiac transplantation. JIMD Reports, 40, 91–95. https://doi.org/10.1007/8904_2017_68

Carmona, S. M. G., Abacan, M. A. R., & Alcausin, M. M. L. B. (2023) Carnitine-acylcarnitine Translocase Deficiency with c.199-10T>G Mutation in Two Filipino Neonates Detected through Parental Carrier Testing. *Int J Neonatal Screen*, 9, 4. doi: 10.3390/ijns9010004.

Chalmers, R. A., Stanley, C. A., English, N., & Wigglesworth, J. S. (1997). Mitochondrial carnitine-acylcarnitine translocase deficiency presenting as sudden neonatal death: Postmortem diagnosis, prenatal exclusion in subsequent pregnancies, and biochemical specificity of the mitochondrial translocase. Journal of Pediatrics, 131(2), 220–225. https://doi.org/10.1016/S0022-3476(97)70157-4

Chen, M., Cai, Y., Li, S., Xiong, H., Liu, M., Ma, F., Xiao, X., & Hao, H. (2020). Late-Onset Carnitine–Acylcarnitine Translocase Deficiency With SLC25A20 c.199-10T>G Variation: Case Report and Pathologic Analysis of Liver Biopsy. Frontiers in Pediatrics, 8. https://doi.org/10.3389/FPED.2020.585646

Chen, Y. C., Chien, Y. H., Chen, P. W., Leung-Sang Tang, N., Chiu, P. C., Hwu, W. L., & Lee, N. C. (2013). Carnitine Uptake Defect (Primary Carnitine Deficiency): Risk in Genotype-Phenotype Correlation. Human Mutation, 34(4), 655. https://doi.org/10.1002/humu.22286

Chien, Y. H., Lee, N. C., Chao, M. C., Chen, L. C., Chen, L. H., Chien, C. C., Ho, H. C., Suen, J. H., & Hwu, W. L. (2013). Fatty acid oxidation disorders in a chinese population in Taiwan. JIMD Reports, 11, 165–172. https://doi.org/10.1007/8904_2013_236

Chinen, Y., Yanagi, K., Nakamura, S., Nakayama, N., Kamiya, M., Nakayashiro, M., Kaname, T., Naritomi, K., & Nakanishi, K. (2020). A novel homozygous missense SLC25A20 mutation in three CACT-deficient patients: clinical and autopsy data. Human Genome Variation, 7(1). https://doi.org/10.1038/S41439-020-0098-Y

Cho, S. Y., Siu, T. S., Ma, O., Tam, S., & Lam, C. wan. (2013). Novel mutations in myopathic form of carnitine palmitoyltransferase II deficiency in a chinese patient. Clinica Chimica Acta, 425, 125–127. https://doi.org/10.1016/J.CCA.2013.07.018

Choi, J. S., Yoo, H. W., Lee, K. J., Ko, J. M., Moon, J. S., & Ko, J. S. (2016). Novel mutations in the CPT1A gene identified in the patient presenting jaundice as the first manifestation of carnitine palmitoyltransferase 1A deficiency. Pediatric Gastroenterology, Hepatology and Nutrition, 19(1), 76–81. https://doi.org/10.5223/PGHN.2016.19.1.76

Chong, S. C., Law, L. K., Hui, J., Lai, C. Y., Leung, T. Y., & Yuen, Y. P. (2017). Expanded newborn metabolic screening programme in Hong Kong: A three-year journey. Hong Kong Medical Journal, 23(5), 489–496. https://doi.org/10.12809/HKMJ176274

Chung, H., Choi, D., Gregor, A., Sim, E., Lau, A., Black, D., Scanga, H. L., Linshinski, A., Pennesi, M. E., Sahel, J.-A., Nischal, K. K., Yang, P., Everett, L., Vockley, J., Matern, D., Harding, C. O., & Gillingham, M. B. (2025). Plasma Metabolomics, Lipidomics, and Acylcarnitines Are Associated With Vision and Genotype but Not With Dietary Intake in Long-Chain 3-Hydroxyacyl-CoA Dehydrogenase Deficiency (LCHADD). Journal of Inherited Metabolic Disease, 48(4), e70060. https://doi.org/10.1002/jimd.70060

Corr, P. B., Creer, M. H., Yamada, K. A., Saffitz, J. E., & Sobel, B. E. (1989). Prophylaxis of early ventricular fibrillation by inhibition of acylcarnitine accumulation. The Journal of Clinical Investigation, 83(3), 927–936. https://doi.org/10.1172/JCI113978

Corti, S., Bordoni, A., Ronchi, D., Musumeci, O., Aguennouz, M., Toscano, A., Lamperti, C., Bresolin, N., & Comi, G. P. (2008). Clinical features and new molecular findings in Carnitine Palmitoyltransferase II (CPT II) deficiency. Journal of the Neurological Sciences, 266(1–2), 97–103. https://doi.org/10.1016/J.JNS.2007.09.015

Costa, C., Costa, J. M., Slama, A., Boutron, A., Vequaud, C., Legrand, A., & Brivet, M. (2003). Mutational spectrum and DNA-based prenatal diagnosis in carnitine-acylcarnitine translocase deficiency. Molecular Genetics and Metabolism, 78(1), 68–73. https://doi.org/10.1016/S1096-7192(02)00205-6

Costa, C. G., Dorland, L., Tavares De Almeida, I., Jakobs, C., Duran, M., & Poll-The, B. T. (1998). The effect of fasting, long-chain triglyceride load and carnitine load on plasma long-chain acylcarnitine levels in mitochondrial very long-chain acyl-CoA dehydrogenase deficiency. Journal of Inherited Metabolic Disease, 21(4), 391–399. https://doi.org/10.1023/A:1005354624735

Coughlin, C. R., & Ficicioglu, C. (2010). Genotype-phenotype correlations: Sudden death in an infant with very-long-chain acyl-CoA dehydrogenase deficiency. Journal of Inherited Metabolic Disease, 33(SUPPL. 3). https://doi.org/10.1007/S10545-009-9041-6

Cox, G. F., Souri, M., Aoyama, T., Rockenmacher, S., Varvogli, L., Rohr, F., Hashimoto, T., & Korson, M. S. (1998). Reversal of severe hypertrophic cardiomyopathy and excellent neuropsychologic outcome in very-long-chain acyl-coenzyme A dehydrogenase deficiency. Journal of Pediatrics, 133(2), 247–253. https://doi.org/10.1016/S0022-3476(98)70228-8

Das, A. M., Illsinger, S., Lücke, T., Hartmann, H., Ruiter, J. P. N., Steuerwald, U., Waterham, H. R., Duran, M., & Wanders, R. J. A. (2006). Isolated mitochondrial long-chain ketoacyl-CoA thiolase deficiency resulting from mutations in the HADHB gene. Clinical Chemistry, 52(3), 530–534. https://doi.org/10.1373/CLINCHEM.2005.062000

Das, A. M., Steuerwald, U., & Illsinger, S. (2010). Inborn errors of energy metabolism associated with myopathies. Journal of Biomedicine and Biotechnology, 2010. https://doi.org/10.1155/2010/340849

De Biase, I., Champaigne, N. L., Schroer, R., Pollard, L. M., Longo, N., & Wood, T. (2012). Primary carnitine deficiency presents atypically with long QT syndrome: A case report. JIMD Reports, 2, 87–90. https://doi.org/10.1007/8904_2011_52

De Biase, I., Viau, K. S., Liu, A., Yuzyuk, T., Botto, L. D., Pasquali, M., & Longo, N. (2017). Diagnosis, treatment, and clinical outcome of patients with mitochondrial trifunctional protein/long-chain 3-hydroxy acyl-CoA dehydrogenase deficiency. JIMD Reports, 31, 63–71. https://doi.org/10.1007/8904_2016_558

de Sain-van der Velden, M. G. M., Diekman, E. F., Jans, J. J., van der Ham, M., Prinsen, B. H. C. M. T., Visser, G., & Verhoeven-Duif, N. M. (2013). Differences between acylcarnitine profiles in plasma and bloodspots. Molecular Genetics and Metabolism, 110(1–2), 116–121. https://doi.org/10.1016/J.YMGME.2013.04.008

Den Boer, M. E. J., Dionisi-Vici, C., Chakrapani, A., Van Thuijl, A. O. J., Wanders, R. J. A., & Wijburg, F. A. (2003). Mitochondrial trifunctional protein deficiency: A severe fatty acid oxidation disorder with cardiac and neurologic involvement. Journal of Pediatrics, 142(6), 684–689. https://doi.org/10.1067/mpd.2003.231

Den Boer, M. E. J., Ijlst, L., Wijburg, F. A., Oostheim, W., Van Werkhoven, M. A., Van Pampus, M. G., Heymans, H. S. A., & Wanders, R. J. A. (2000). Heterozygosity for the common LCHAD mutation (1528G>C) is not a major cause of HELLP syndrome and the prevalence of the mutation in the dutch population is low. Pediatric Research, 48(2), 151–154. https://doi.org/10.1203/00006450-200008000-00006

Den Boer, M. E. J., Wanders, R. J. A., Morris, A. A. M., Ijlst, L., Heymans, H. S. A., & Wijburg, F. A. (2002). Long-chain 3-hydroxyacyl-CoA dehydrogenase deficiency: Clinical presentation and follow-up of 50 patients. Pediatrics, 109(1), 99–104. https://doi.org/10.1542/PEDS.109.1.99

Dessein, A. F., Hebbar, E., Vamecq, J., Lebredonchel, E., Devos, A., Ghoumid, J., Mention, K., Dobbelaere, D., Chevalier-Curt, M. J., Fontaine, M., Defoort, S., Smirnov, V., Douillard, C., & Dhaenens, C. M. (2022). A novel HADHA variant associated with an atypical moderate and late-onset LCHAD deficiency. Molecular Genetics and Metabolism Reports, 31. https://doi.org/10.1016/J.YMGMR.2022.100860

Deutsch, M., Vassilopoulos, D., Sevastos, N., Papadimitriou, A., Vasiliou, K., & Archimandritis, A. J. (2008). Severe rhabdomyolysis with hypoglycemia in an adult patient with carnitine palmitoyltransferase II deficiency. European Journal of Internal Medicine, 19(4), 289–291. https://doi.org/10.1016/J.EJIM.2007.04.025

Diebold, I., Schön, U., Horvath, R., Schwartz, O., Holinski-Feder, E., Kölbel, H., & Abicht, A. (2019). HADHA and HADHB gene associated phenotypes - Identification of rare variants in a patient cohort by Next Generation Sequencing. Molecular and Cellular Probes, 44, 14–20. https://doi.org/10.1016/J.MCP.2019.01.003

Diekman, E., de Sain-van der Velden, M., Waterham, H., Kluijtmans, L., Schielen, P., van Veen, E. Ben, Ferdinandusse, S., Wijburg, F., & Visser, G. (2016). The newborn screening paradox: Sensitivity vs. overdiagnosis in VLCAD deficiency. JIMD Reports, 27, 101–106. https://doi.org/10.1007/8904_2015_476

Diekman, E. F., Boelen, C. C. A., Prinsen, B. H. C. M. T., IJlst, L., Duran, M., de Koning, T. J., Waterham, H. R., Wanders, R. J. A., Wijburg, F. A., & Visser, G. (2013). Necrotizing enterocolitis and respiratory distress syndrome as first clinical presentation of mitochondrial trifunctional protein deficiency. JIMD Reports, 7, 1–6. https://doi.org/10.1007/8904_2012_128

Diekman, E. F., Ferdinandusse, S., Van Der Pol, L., Waterham, H. R., Ruiter, J. P. N., IJlst, L., Wanders, R. J., Houten, S. M., Wijburg, F. A., Blank, A. C., Asselbergs, F. W., Houtkooper, R. H., & Visser, G. (2015). Fatty acid oxidation flux predicts the clinical severity of VLCAD deficiency. Genetics in Medicine, 17(12), 989–994. https://doi.org/10.1038/GIM.2015.22

Diekman, E. F., Van Der Pol, W. L., Nievelstein, R. A. J., Houten, S. M., Wijburg, F. A., & Visser, G. (2014). Muscle MRI in patients with long-chain fatty acid oxidation disorders. Journal of Inherited Metabolic Disease, 37(3), 405–413. https://doi.org/10.1007/S10545-013-9666-3

Diekman, E. F., Visser, G., Schmitz, J. P. J., Nievelstein, R. A. J., De Sain-van Der Velden, M., Wardrop, M., Van Der Pol, W. L., Houten, S. M., Van Riel, N. A. W., Takken, T., & Jeneson, J. A. L. (2016). Altered energetics of exercise explain risk of rhabdomyolysis in very long-chain acyl-coa dehydrogenase deficiency. PLoS ONE, 11(2). https://doi.org/10.1371/JOURNAL.PONE.0147818

Doi, T., Abo, W., Tateno, M., Hayashi, K., Hori, T., Nakada, T., Fukao, T., Takahashi, Y., & Terada, N. (2000). Milder childhood form of very long-chain acyl-CoA dehydrogenase deficiency in a 6-year-old Japanese boy. European Journal of Pediatrics, 159(12), 908–911. https://doi.org/10.1007/PL00008368

Donald, E., Leb, J., Bialer, M., & Axsom, K. (2020). A Rare Presentation of Cardiomyopathy in Pregnancy. JACC: Case Reports, 2(7), 1066–1069. https://doi.org/10.1016/J.JACCAS.2020.05.022

Dulz, S., Atiskova, Y., Engel, P., Wildner, J., Tsiakas, K., & Santer, R. (2021). Retained visual function in a subset of patients with long-chain 3-hydroxyacyl-CoA dehydrogenase deficiency (LCHADD). Ophthalmic Genetics, 42(1), 23–27. https://doi.org/10.1080/13816810.2020.1836658

Dyke, P. C., Konczal, L., Bartholomew, D., McBride, K. L., & Hoffman, T. M. (2009). Acute dilated cardiomyopathy in a patient with deficiency of long-chain 3-hydroxyacyl-CoA dehydrogenase. Pediatric Cardiology, 30(4), 523–526. https://doi.org/10.1007/S00246-008-9351-8

Dykema, D. M. (2012). Carnitine Palmitoyltransferase-1A Deficiency. Advances in Neonatal Care, 12(1), 23–27. https://doi.org/10.1097/ANC.0b013e318242df6d

Edmondson, A. C., Salant, J., Ierardi-Curto, L. A., & Ficicioglu, C. (2017). Missed newborn screening case of carnitine palmitoyltransferase-II deficiency. JIMD Reports, 33, 93–97. https://doi.org/10.1007/8904_2016_528

El-Gharbawy, A., & Vockley, J. (2018). Inborn Errors of Metabolism with Myopathy: Defects of Fatty Acid Oxidation and the Carnitine Shuttle System. Pediatric Clinics of North America, 65(2), 317–335. https://doi.org/10.1016/J.PCL.2017.11.006

Elizondo, G., Matern, D., Vockley, J., Harding, C. O., & Gillingham, M. B. (2020). Effects of fasting, feeding and exercise on plasma acylcarnitines among subjects with CPT2D, VLCADD and LCHADD/TFPD. Molecular Genetics and Metabolism, 131(1–2), 90–97. https://doi.org/10.1016/j.ymgme.2020.09.001

Eminoglu, T. F., Tumer, L., Okur, I., Ezgu, F. S., Biberoglu, G., & Hasanoglu, A. (2011). Very long-chain acyl CoA dehydrogenase deficiency which was accepted as infanticide. Forensic Science International, 210(1–3), e1. https://doi.org/10.1016/J.FORSCIINT.2011.04.003

Emura, I., & Usuda, H. (2003). Morphological investigation of two sibling autopsy cases of mitochondrial trifunctional protein deficiency. Pathology International, 53(11), 775–779. https://doi.org/10.1046/j.1440-1827.2003.01558.x

Engbers, H. M., Dorland, L., De Sain, M. G. M., Eskes, P. F., & Visser, G. (2005). Rhabdomyolysis in early-onset very long-chain acyl-CoA dehydrogenase deficiency despite normal glucose after fasting. Journal of Inherited Metabolic Disease, 28(6), 1151–1152. https://doi.org/10.1007/S10545-005-0190-Y

Evans, M., Andresen, B. S., Nation, J., & Boneh, A. (2016). VLCAD deficiency: Follow-up and outcome of patients diagnosed through newborn screening in Victoria. Molecular Genetics and Metabolism, 118(4), 282–287. https://doi.org/10.1016/J.YMGME.2016.05.012

Fahnehjelm, K. T., Holmström, G., Ying, L., Haglind, C. B., Nordenström, A., Halldin, M., Alm, J., Nemeth, A., & Von Döbeln, U. (2008). Ocular characteristics in 10 children with long-chain 3-hydroxyacyl-CoA dehydrogenase deficiency: A cross-sectional study with long-term follow-up. Acta Ophthalmologica, 86(3), 329–337. https://doi.org/10.1111/J.1600-0420.2007.01121.X

Fahnehjelm, K. T., Liu, Y., Olsson, D., Amrén, U., Haglind, C. B., Holmström, G., Halldin, M., Andreasson, S., & Nordenström, A. (2016). Most patients with long-chain 3-hydroxyacyl-CoA dehydrogenase deficiency develop pathological or subnormal retinal function. Acta Paediatrica, International Journal of Paediatrics, 105(12), 1451–1460. https://doi.org/10.1111/APA.13536

Fanin, M., Anichini, A., Cassandrini, D., Fiorillo, C., Scapolan, S., Minetti, C., Cassanello, M., Donati, M. A., Siciliano, G., D’Amico, A., Lilliu, F., Bruno, C., & Angelini, C. (2012). Allelic and phenotypic heterogeneity in 49 Italian patients with the muscle form of CPT-II deficiency. Clinical Genetics, 82(3), 232–239. https://doi.org/10.1111/J.1399-0004.2011.01786.X

Fatehi, F., Okhovat, A. A., Nilipour, Y., Mroczek, M., Straub, V., Töpf, A., Palibrk, A., Peric, S., Rakocevic Stojanovic, V., Najmabadi, H., & Nafissi, S. (2020). Adult-onset very-long-chain acyl-CoA dehydrogenase deficiency (VLCADD). European Journal of Neurology, 27(11), 2257–2266. https://doi.org/10.1111/ENE.14402

Faulkner, M. S. (1999). Long-chain 3-hydroxyacyl-CoA dehydrogenase deficiency: a case example in developmental disabilities. Journal of Pediatric Nursing, 14(6), 359–368. https://doi.org/10.1016/S0882-5963(99)80065-7

Fernandez-Guerra, P., Lund, M., Corydon, T. J., Cornelius, N., Gregersen, N., Palmfeldt, J., & Bross, P. (2016). Application of an image cytometry protocol for cellular and mitochondrial phenotyping on fibroblasts from patients with inherited disorders. JIMD Reports, 27, 17–26. https://doi.org/10.1007/8904_2015_494

Fernandez-Lainez, C., Aguilar-Lemus, JJ., Vela-Amieva, M., & Ibarra-Gonzalez, I. (2012). Tandem Mass Spectrometry Newborn Screening for Inborn Errors of Intermediary Metabolism: Abnormal Profile Interpretation. Current Medicinal Chemistry, 19(26), 4511–4522. https://doi.org/10.2174/092986712803251539

Ferrara, A. M., Sciacco, M., Zovato, S., Rizzati, S., Colombo, I., Boaretto, F., Moggio, M., & Opocher, G. (2016). Coexistence of VHL disease and CPT2 deficiency: A case report. Cancer Research and Treatment, 48(4), 1438–1442. https://doi.org/10.4143/CRT.2015.450

Ficicioglu, C., Coughlin, C. R., Bennett, M. J., & Yudkoff, M. (2010). Very Long-Chain Acyl-CoA Dehydrogenase Deficiency in a Patient with Normal Newborn Screening by Tandem Mass Spectrometry. Journal of Pediatrics, 156(3), 492–494. https://doi.org/10.1016/J.JPEDS.2009.10.031

Fingerhut, R., Röschinger, W., Muntau, A. C., Dame, T., Kreischer, J., Arnecke, R., Superti-Furga, A., Troxler, H., Liebl, B., Olgemöller, B., & Roscher, A. A. (2001). Hepatic carnitine palmitoyltransferase I deficiency: Acylcarnitine profiles in blood spots are highly specific. Clinical Chemistry, 47(10), 1763–1768. https://doi.org/10.1093/clinchem/47.10.1763

Finsterer, J., & Zarrouk-Mahjoub, S. (2017). Trifunctional protein deficiency due to hadhb mutations is a multisystem, β-oxidation disorder. Archives of Iranian Medicine, 20(12), 767–769.

Fontaine, M., Kim, I., Dessein, A. F., Mention-Mulliez, K., Dobbelaere, D., Douillard, C., Sole, G., Schiff, M., Jaussaud, R., Espil-Taris, C., Boutron, A., Wuyts, W., Acquaviva, C., Vianey-Saban, C., Roland, D., Joncquel-Chevalier Curt, M., & Vamecq, J. (2018). Fluxomic assay-assisted diagnosis orientation in a cohort of 11 patients with myopathic form of CPT2 deficiency. Molecular Genetics and Metabolism, 123(4), 441–448. https://doi.org/10.1016/J.YMGME.2018.02.005

Fraser, H., Geppert, J., Johnson, R., Johnson, S., Connock, M., Clarke, A., Taylor-Phillips, S., & Stinton, C. (2019). Evaluation of earlier versus later dietary management in long-chain 3-hydroxyacyl-CoA dehydrogenase or mitochondrial trifunctional protein deficiency: A systematic review. Orphanet Journal of Rare Diseases, 14(1). https://doi.org/10.1186/S13023-019-1226-Y

Frigeni, M., Balakrishnan, B., Yin, X., Calderon, F. R. O., Mao, R., Pasquali, M., & Longo, N. (2017). Functional and molecular studies in primary carnitine deficiency. Human Mutation, 38(12), 1684–1699. https://doi.org/10.1002/HUMU.23315

Fu, X., Zheng, F., Zhang, Y., Bao, X., Wang, S., Yang, Y., & Xiong, H. (2015). Mitochondrial trifunctional protein deficiency due to HADHB gene mutation in a Chinese family. Molecular Genetics and Metabolism Reports, 5, 80–84. https://doi.org/10.1016/J.YMGMR.2015.10.015

Galron, D., Birk, O. S., Kazanovitz, A., Moses, S. W., & Hershkovitz, E. (2004). Carnitine-acylcarnitine translocase deficiency: Identification of a novel molecular defect in a Bedouin patient. Journal of Inherited Metabolic Disease, 27(2), 267–273. https://doi.org/10.1023/B:BOLI.0000028780.01670.61

Gan, Y., Yu, F., & Fang, H. (2021). Novel mutation in carnitine palmitoyltransferase 1A detected through newborn screening for a presymptomatic case in China: a case report. Italian Journal of Pediatrics, 47(1). https://doi.org/10.1186/S13052-021-01094-5

García García, L. C., Zamorano Martín, F., Rocha de Lossada, C., García Lorente, M., Luque Aranda, G., & Escudero Gómez, J. (2021). Retinitis pigmentosa as a clinical presentation of LCHAD deficiency: A clinical case and review of the literature. Archivos de La Sociedad Española de Oftalmología (English Edition), 96(9), 496–499. https://doi.org/10.1016/J.OFTALE.2020.07.013

Gataullina, S., Dellatolas, G., Perdry, H., Robert, J. J., Valayannopoulos, V., Touati, G., Ottolenghi, C., Dulac, O., & De Lonlay, P. (2012). Comorbidity and metabolic context are crucial factors determining neurological sequelae of hypoglycaemia. Developmental Medicine and Child Neurology, 54(11), 1012–1017. https://doi.org/10.1111/J.1469-8749.2012.04400.X

Gempel, K., Von Praun, C., Baumkötter, J., Lehnert, W., Ensenauer, R., Gerbitz, K. D., & Bauer, M. F. (2001). “Adult” form of muscular carnitine palmitoyltransferase II deficiency: Manifestation in a 2-year-old child. European Journal of Pediatrics, 160(9), 548–551. https://doi.org/10.1007/S004310100802

Gessner, B. D., Gillingham, M. B., Johnson, M. A., Richards, C. S., Lambert, W. E., Sesser, D., Rien, L. C., Hermerath, C. A., Skeels, M. R., Birch, S., Harding, C. O., Wood, T., & Koeller, D. M. (2011). Prevalence and distribution of the c.1436C→T sequence variant of carnitine palmitoyltransferase 1A among Alaska native infants. Journal of Pediatrics, 158(1), 124–129. https://doi.org/10.1016/J.JPEDS.2010.07.031

Giak Sim, K., Carpenter, K., Hammond, J., Christodoulou, J., & Wilcken, B. (2002). Quantitative fibroblast acylcarnitine profiles in mitochondrial fatty acid β-oxidation defects: Phenotype/metabolite correlations. Molecular Genetics and Metabolism, 76(4), 327–334. https://doi.org/10.1016/S1096-7192(02)00112-9

Gilbert-Barness, E. (2004). Review: Metabolic Cardiomyopathy and Conduction System Defleas in Children. Annals of Clinical and Laboratory Science, 34(1), 15–34.

Gillingham, M. B. (2009). Nutrition management of patients with inherited disorders of mitochondrial fatty acid oxidation. In P. B. Acosta (Ed.), Nutrition Management of Patients with Inherited Metabolic Disorders (pp. 369–403). Jones & Bartlett Learning.

Gillingham, M. B. (2012). Managing myopathy in adults and adolescents with long-chain FAO disorders. 35th Annual Meeting of the Society for Inherited Metabolic Disorders.

Gillingham, M. B., Connor, W. E., Matern, D., Rinaldo, P., Burlingame, T., Meeuws, K., & Harding, C. O. (2003). Optimal dietary therapy of long-chain 3-hydroxyacyl-CoA dehydrogenase deficiency. Molecular Genetics and Metabolism, 79(2), 114–123. https://doi.org/10.1016/S1096-7192(03)00073-8

Gillingham, M. B., Elizondo, G., Behrend, A., Matern, D., Schoeller, D. A., Harding, C. O., & Purnell, J. Q. (2019). Higher dietary protein intake preserves lean body mass, lowers liver lipid deposition, and maintains metabolic control in participants with long-chain fatty acid oxidation disorders. Journal of Inherited Metabolic Disease, 42(5), 857–869. https://doi.org/10.1002/JIMD.12155

Gillingham, M. B., Harding, C. O., Schoeller, D. A., Matern, D., & Purnell, J. Q. (2013). Altered body composition and energy expenditure but normal glucose tolerance among humans with a long-chain fatty acid oxidation disorder. American Journal of Physiology - Endocrinology and Metabolism, 305(10). https://doi.org/10.1152/AJPENDO.00225.2013

Gillingham, M. B., Heitner, S. B., Martin, J., Rose, S., Goldstein, A., El-Gharbawy, A. H., Deward, S., Lasarev, M. R., Pollaro, J., DeLany, J. P., Burchill, L. J., Goodpaster, B., Shoemaker, J., Matern, D., Harding, C. O., & Vockley, J. (2017). Triheptanoin versus trioctanoin for long-chain fatty acid oxidation disorders: a double blinded, randomized controlled trial. Journal of Inherited Metabolic Disease, 40(6), 831–843. https://doi.org/10.1007/S10545-017-0085-8

Gillingham, M. B., Hirschfeld, M., Lowe, S., Matern, D., Shoemaker, J., Lambert, W. E., & Koeller, D. M. (2011). Impaired fasting tolerance among Alaska native children with a common carnitine palmitoyltransferase 1A sequence variant. Molecular Genetics and Metabolism, 104(3), 261–264. https://doi.org/10.1016/J.YMGME.2011.06.017

Gillingham, M. B., Matern, D., & Harding, C. O. (2009). EFFECT OF FEEDING, EXERCISE AND GENOTYPE ON PLASMA 3-HYDROXYACYLCARNITINES IN CHILDREN WITH LCHAD DEFICIENCY. Topics in Clinical Nutrition, 24(4), 359–365. https://doi.org/10.1097/TIN.0b013e3181c62182

Gillingham, M. B., Purnell, J. Q., Jordan, J., Stadler, D., Haqq, A. M., & Harding, C. O. (2007). Effects of higher dietary protein intake on energy balance and metabolic control in children with long-chain 3-hydroxy acyl-CoA dehydrogenase (LCHAD) or trifunctional protein (TFP) deficiency. Molecular Genetics and Metabolism, 90(1), 64–69. https://doi.org/10.1016/J.YMGME.2006.08.002

Gillingham, M. B., Scott, B., Elliott, D., & Harding, C. O. (2006). Metabolic control during exercise with and without medium-chain triglycerides (MCT) in children with long-chain 3-hydroxy acyl-CoA dehydrogenase (LCHAD) or trifunctional protein (TFP) deficiency. Molecular Genetics and Metabolism, 89(1–2), 58–63. https://doi.org/10.1016/J.YMGME.2006.06.004

Gillingham, M. B., Weleber, R. G., Neuringer, M., Connor, W. E., Mills, M., Van Calcar, S., Ver Hoeve, J., Wolff, J., & Harding, C. O. (2005). Effect of optimal dietary therapy upon visual function in children with long-chain 3-hydroxyacyl CoA dehydrogenase and trifunctional protein deficiency. Molecular Genetics and Metabolism, 86(1–2), 124–133. https://doi.org/10.1016/J.YMGME.2005.06.001

Gillingham, M., Guillory, K., Martin, J., Matern, D., Harding, C. O., & Vockley, J. (2017). The comparison of the digestion and absorption of trioctanoin (C8) and triheptanoin (C7) in patients with long-chain fatty acid oxidation disorders. 13th International Congress of Inborn Errors of Metabolism. https://doi.org/10.1177/2326409817722292

Gillingham, M., Van Calcar, S., Ney, D., Wolff, J., & Harding, C. (1999). Dietary management of long-chain 3-hydroxyacyl-CoA dehydrogenase deficiency (LCHADD). A case report and survey. Journal of Inherited Metabolic Disease, 22(2), 123–131. https://doi.org/10.1023/A:1005437616934

Giuliani, E., Coppi, F., Bertolotti, V., Gorlato, G., Zavatta, M., & Barbieri, A. (2013). Critical illness in energy metabolism genetic disorder: Rhabdomyolysis, acute kidney injury, respiratory arrest. West Indian Medical Journal, 62(8), 773–775. https://doi.org/10.7727/WIMJ.2012.237

Glamuzina, E., Dixon, M., Prunty, H., Pitt, M., Russell- Eggitt, I., Abulhoul, L., Clayton, P., & Grunewald, S. (2012). Disorders of long-chain fatty acid oxidation:Management and outcomes in a single centre in the United Kingdom. Annual Symposium of the Society for the Study of Inborn Errors of Metabolism. https://doi.org/10.1007/s10545-012-9512-z

Gobin Limballe, S., McAndrew, R. P., Djouadi, F., Kim, J. J., & Bastin, J. (2010). Compared effects of missense mutations in Very-Long-Chain Acyl-CoA Dehydrogenase deficiency: Combined analysis by structural, functional and pharmacological approaches. Biochimica et Biophysica Acta - Molecular Basis of Disease, 1802(5), 478–484. https://doi.org/10.1016/J.BBADIS.2010.01.001

Greenberg, C. R., Dilling, L. A., Thompson, G. R., Seargeant, L. E., Haworth, J. C., Phillips, S., Chan, A., Vallance, H. D., Waters, P. J., Sinclair, G., Lillquist, Y., Wanders, R. J. A., & Olpin, S. E. (2009). The paradox of the carnitine palmitoyltransferase type Ia P479L variant in Canadian Aboriginal populations. Molecular Genetics and Metabolism, 96(4), 201–207. https://doi.org/10.1016/J.YMGME.2008.12.018

Grünert, S. C., Eckenweiler, M., Haas, D., Lindner, M., Tsiakas, K., Santer, R., Tucci, S., & Spiekerkoetter, U. (2021). The spectrum of peripheral neuropathy in disorders of the mitochondrial trifunctional protein. Journal of Inherited Metabolic Disease, 44(4), 893–902. https://doi.org/10.1002/JIMD.12372

Grünert, S. C., Eckenweiler, M., & Spiekerkoetter, U. (2022). Reversible sensory neuropathy in mitochondrial trifunctional protein deficiency. JIMD Reports, 63(3), 207–210. https://doi.org/10.1002/JMD2.12279

Grünewald, S., Bakkeren, J., Wanders, R. A., & Wendel, U. (1997). Neonatal lethal mitochondrial trifunctional protein deficiency mimicking a respiratory chain defect. Journal of Inherited Metabolic Disease, 20(6), 835–836. https://doi.org/10.1023/A:1005340322558

Guan, Y., Zhang, Y., Shen, X. M., Zhou, L., Shang, X., Peng, Y., Hu, Y., & Li, W. (2021). Charcot–Marie–Tooth Disease With Episodic Rhabdomyolysis Due to Two Novel Mutations in the β Subunit of Mitochondrial Trifunctional Protein and Effective Response to Modified Diet Therapy. Frontiers in Neurology, 12. https://doi.org/10.3389/FNEUR.2021.694966

Guffon, N., Mochel, F., Schiff, M., De Lonlay, P., Douillard, C., & Vianey-Saban, C. (2021). Clinical outcomes in a series of 18 patients with long chain fatty acids oxidation disorders treated with triheptanoin for a median duration of 22 months. Molecular Genetics and Metabolism, 132(4), 227–233. https://doi.org/10.1016/J.YMGME.2021.02.003

Gürbüz, B. B., Yılmaz, D. Y., Özgül, R. K., Koşukcu, C., Dursun, A., Sivri, H. S., Coşkun, T., & Tokatlı, A. (2021). Clinical and molecular characteristics of carnitineacylcarnitine translocase deficiency with c.270delC and a novel c.408C&gt;A variant. The Turkish Journal of Pediatrics, 63(4), 691–696. https://doi.org/10.24953/turkjped.2021.04.017

Gutiérrez Junquera, C., Balmaseda, E., Gil, E., Martínez, A., Sorli, M., Cuartero, I., Merinero, B., & Ugarte, M. (2009). Acute fatty liver of pregnancy and neonatal long-chain 3-hydroxyacyl- coenzyme a dehydrogenase (LCHAD) deficiency. European Journal of Pediatrics, 168(1), 103–106. https://doi.org/10.1007/S00431-008-0696-Z

Habib, A., Azize, N. A. A., Rahman, S. A., Yakob, Y., Suberamaniam, V., Nazri, M. I. B. A., Abdullah Sani, H., Ch’ng, G. S., Yin, L. H., Olpin, S., & Lock-Hock, N. (2021). Novel mutations associated with carnitine-acylcarnitine translocase and carnitine palmitoyl transferase 2 deficiencies in Malaysia. Clinical Biochemistry, 98, 48–53. https://doi.org/10.1016/J.CLINBIOCHEM.2021.10.002

Haglind, C. B., Nordenström, A., Ask, S., von Döbeln, U., Gustafsson, J., & Stenlid, M. H. (2015). Increased and early lipolysis in children with long-chain 3-hydroxyacyl-CoA dehydrogenase (LCHAD) deficiency during fast. Journal of Inherited Metabolic Disease, 38(2), 315–322. https://doi.org/10.1007/S10545-014-9750-3

Haglind, C. B., Stenlid, M. H., Ask, S., Alm, J., Nemeth, A., Döbeln, U., & Nordenström, A. (2013). Growth in long-chain 3-Hydroxyacyl-CoA dehydrogenase deficiency. JIMD Reports, 8, 81–90. https://doi.org/10.1007/8904_2012_164

Hahn, S. H., Lee, E. H., Jung, J. W., Hong, C. H., Yoon, H. R., Rinaldo, P., Sims, H., Gibson, B., & Strauss, A. W. (1999). Very long chain acyl coenzyme A dehydrogenase deficiency in a 5-month- old Korean boy: Identification of a novel mutation. Journal of Pediatrics, 135(2 I), 250–253. https://doi.org/10.1016/S0022-3476(99)70030-2

Halldin, M. U., Forslund, A., von Döbeln, U., Eklund, C., & Gustafsson, J. (2007). Increased lipolysis in LCHAD deficiency. Journal of Inherited Metabolic Disease, 30(1), 39–46. https://doi.org/10.1007/s10545-006-0296-x

Harding, C. O., Scott, B., & Gillingham, M. B. (2005). MCT supplementation immediately prior to exercise improves exercise tolerance in children with long-chain 3-hydroxy acyl-CoA dehydrogenase (LCHAD) deficiency. Annual Meeting of the Society for Inherited Metabolic Disorders .

Harding Cary O. (2017). Fatty Acid Beta-Oxidation Defects. In Metabolic Diseases. IOS Press. https://doi.org/10.3233/978-1-61499-718-4-155

Hayes, B., Lynch, B., O’Keefe, M., Monavari, A. A., & Treacy, E. P. (2007). Long chain fatty acid oxidation defects in children: Importance of detection and treatment options. Irish Journal of Medical Science, 176(3), 189–192. https://doi.org/10.1007/S11845-007-0025-Y

He, G., Yang, B. Z., Roe, D. S., Teramoto, R., Aleck, K., Grebe, T. A., Roe, C. R., & Ding, J. H. (1999). Identification of two novel mutations in the hypoglycemic phenotype of very long chain Acyl-CoA dehydrogenase deficiency. Biochemical and Biophysical Research Communications, 264(2), 483–487. https://doi.org/10.1006/BBRC.1999.1411

Hebbar, S., Shashidhara, S., Mundkur, S., & Kanaparthi, S. (2018). A Clinical and Biochemical Camouflage-Carnitine Palmitoyltransferase-1 Deficiency: A Case Series. JOURNAL OF CLINICAL AND DIAGNOSTIC RESEARCH. https://doi.org/10.7860/JCDR/2018/29861.11164

Herrera-Olivares, A. M., Fernández-Luque, J. A., Paradas, C., Lucia, A., & Santalla, A. (2019). Combined HIIT and resistance training in very long-chain acyl-CoA dehydrogenase deficiency: A case report. Frontiers in Physiology, 10(MAY). https://doi.org/10.3389/FPHYS.2019.00650

Hesse, J., Braun, C., Behringer, S., Matysiak, U., Spiekerkoetter, U., & Tucci, S. (2018). The diagnostic challenge in very-long chain acyl-CoA dehydrogenase deficiency (VLCADD). Journal of Inherited Metabolic Disease, 41(6), 1169–1178. https://doi.org/10.1007/S10545-018-0245-5

Hindawy, A., Gouda, A., El-Ayyadi, A., Megahed, H., & Bazaraa, H. (2007). Metabolic encephalopathy in Egyptian children. Bratislavské Lekárske Listy, 108(2), 75–82.

Hintz, S. R., Matern, D., Strauss, A., Bennett, M. J., Hoyme, H. E., Schelley, S., Kobori, J., Colby, C., Lehman, N. L., & Enns, G. M. (2002). Early neonatal diagnosis of long-chain 3-hydroxyacyl coenzyme a dehydrogenase and mitochondrial trifunctional protein deficiencies. Molecular Genetics and Metabolism, 75(2), 120–127. https://doi.org/10.1006/MGME.2001.3282

Hisahara, S., Matsushita, T., Furuyama, H., Tajima, G., Shigematsu, Y., Imai, T., & Shimohama, S. (2015). A heterozygous missense mutation in adolescent-onset very long-chain acyl-coa dehydrogenase deficiency with exercise-induced rhabdomyolysis. Tohoku Journal of Experimental Medicine, 235(4), 305–310. https://doi.org/10.1620/TJEM.235.305

Hoffman, J. D., Steiner, R. D., Paradise, L., Harding, C. O., Ding, L., Strauss, A. W., & Kaplan, P. (2006). Rhabdomyolysis in the military: Recognizing late-onset very long-chain acyl Co-A dehydrogenase deficiency. Military Medicine, 171(7), 657–658. https://doi.org/10.7205/MILMED.171.7.657

Hoffmann, L., Haussmann, U., Mueller, M., & Spiekerkoetter, U. (2012). VLCAD enzyme activity determinations in newborns identified by screening: A valuable tool for risk assessment. Journal of Inherited Metabolic Disease, 35(2), 269–277. https://doi.org/10.1007/S10545-011-9391-8

Holub, M., Bodamer, O. A., Item, C., Mühl, A., Pollak, A., & Stöckler-Ipsiroglu, S. (2005). Lack of correlation between fatty acid oxidation disorders and haemolysis, elevated liver enzymes, low platelets (HELLP) syndrome? Acta Paediatrica (Oslo, Norway : 1992), 94(1), 48–52. https://doi.org/10.1111/j.1651-2227.2005.tb01787.x

Hong, Y. B., Lee, J. H., Park, J. M., Choi, Y. R., Hyun, Y. S., Yoon, B. R., Yoo, J. H., Koo, H., Jung, S. C., Chung, K. W., & Choi, B. O. (2013). A compound heterozygous mutation in HADHB gene causes an axonal Charcot-Marie-tooth disease. BMC Medical Genetics, 14(1). https://doi.org/10.1186/1471-2350-14-125

Howat, A. J., Bennett, M. J., Variend, S., Shaw, L., & Engel, P. C. (1985). Defects of metabolism of fatty acids in the sudden infant death syndrome. BMJ, 290(6484), 1771–1773. https://doi.org/10.1136/bmj.290.6484.1771

Iacobazzi, V., Invernizzi, F., Baratta, S., Pons, R., Chung, W., Garavaglia, B., Dionisi-Vici, C., Ribes, A., Parini, R., Huertas, M. D., Roldan, S., Lauria, G., Palmieri, F., & Taroni, F. (2004). Molecular and functional analysis of SLC25A20 mutations causing carnitine-acylcarnitine translocase deficiency. Human Mutation, 24(4), 312–320. https://doi.org/10.1002/HUMU.20085

Iacobazzi, V., Pasquali, M., Singh, R., Matern, D., Rinaldo, P., di San Filippo, C. A., Palmieri, F., & Longo, N. (2004). Response to therapy in carnitine/acylcarnitine translocase (CACT) deficiency due to a novel missense mutation. American Journal of Medical Genetics Part A, 126A(2), 150–155. https://doi.org/10.1002/ajmg.a.20573

Ibdah, J. A., Bennett, M. J., Rinaldo, P., Zhao, Y., Gibson, B., Sims, H. F., & Strauss, A. W. (1999). A fetal fatty-acid oxidation disorder as a cause of liver disease in pregnant women. The New England Journal of Medicine, 340(22), 1723–1731. https://doi.org/10.1056/NEJM199906033402204

Ibdah, J. A., Tein, I., Dionisi-Vici, C., Bennett, M. J., Ijlst, L., Gibson, B., Wanders, R. J. A., & Strauss, A. W. (1998). Mild trifunctional protein deficiency is associated with progressive neuropathy and myopathy and suggests a novel genotype-phenotype correlation. Journal of Clinical Investigation, 102(6), 1193–1199. https://doi.org/10.1172/JCI2091

Ijlst, L., Ruiter, J. P. N., Hoovers, J. M. N., Jakobs, M. E., & Wanders, R. J. A. (1996). Common missense mutation G1528C in long-chain 3-hydroxyacyl-CoA dehydrogenase deficiency: Characterization and expression of the mutant protein, mutation analysis on genomic DNA and chromosomal localization of the mitochondrial trifunctional protein α subunit gene. Journal of Clinical Investigation, 98(4), 1028–1033. https://doi.org/10.1172/JCI118863

Illsinger, S., Lücke, T., Peter, M., Ruiter, J. P. N., Wanders, R. J. A., Deschauer, M., Handig, I., Wuyts, W., & Das, A. M. (2008). Carnitine-palmitoyltransferase 2 deficiency: Novel mutations and relevance of newborn screening. American Journal of Medical Genetics, Part A, 146(22), 2925–2928. https://doi.org/10.1002/AJMG.A.32545

Immonen, T., Turanlahti, M., Paganus, A., Keskinen, P., Tyni, T., & Lapatto, R. (2016). Earlier diagnosis and strict diets improve the survival rate and clinical course of long-chain 3-hydroxyacyl-CoA dehydrogenase deficiency. Acta Paediatrica, International Journal of Paediatrics, 105(5), 549–554. https://doi.org/10.1111/APA.13313

Isackson, P. J., Bennett, M. J., Lichter-Konecki, U., Willis, M., Nyhan, W. L., Sutton, V. R., Tein, I., & Vladutiu, G. D. (2008). CPT2 gene mutations resulting in lethal neonatal or severe infantile carnitine palmitoyltransferase II deficiency. Molecular Genetics and Metabolism, 94(4), 422–427. https://doi.org/10.1016/J.YMGME.2008.05.002

Ivin, N., Della Torre, V., Sanders, F., & Youngman, M. (2020). Rhabdomyolysis caused by carnitine palmitoyltransferase 2 deficiency: A case report and systematic review of the literature. Journal of the Intensive Care Society, 21(2), 165–173. https://doi.org/10.1177/1751143719889766

Jackson, S., Bartlett, K., Land, J., Moxon, E. R., Pollitt, R. J., Leonard, J. V, & Turnbull, D. M. (1991). Long-Chain 3-Hydroxyacyl-CoA Dehydrogenase Deficiency. Pediatric Research, 29(4), 406–411. https://doi.org/10.1203/00006450-199104000-00016

Janeiro, P., Jotta, R., Ramos, R., Florindo, C., Ventura, F. V., Vilarinho, L., Tavares de Almeida, I., & Gaspar, A. (2019). Follow-up of fatty acid β-oxidation disorders in expanded newborn screening era. European Journal of Pediatrics, 178(3), 387–394. https://doi.org/10.1007/S00431-018-03315-2

Jiang, M., Liu, L., Mei, H., Li, X., Cheng, J., & Cai, Y. (2015). Detection of inborn errors of metabolism using GC-MS: Over 3 years of experience in southern China. Journal of Pediatric Endocrinology and Metabolism, 28(3–4), 375–380. https://doi.org/10.1515/JPEM-2014-0164

Joshi, P. R., Deschauer, M., & Zierz, S. (2012). Clinically symptomatic heterozygous carnitine palmitoyltransferase II (CPT II) deficiency. Wiener Klinische Wochenschrift, 124(23–24), 851–854. https://doi.org/10.1007/S00508-012-0296-9

Joshi, P. R., Deschauer, M., & Zierz, S. (2014). Carnitine palmitoyltransferase II (CPT II) deficiency: Genotype-Phenotype analysis of 50 patients. Journal of the Neurological Sciences, 338(1–2), 107–111. https://doi.org/10.1016/J.JNS.2013.12.026

Joshi, P. R., Deschauer, M., & Zierz, S. (2019). Phenotype of carnitine palmitoyltransferase II (CPT II) deficiency: A questionnaire-based survey. Journal of Clinical Neuroscience, 59, 32–36. https://doi.org/10.1016/j.jocn.2018.11.023

Kaluzny, L., Siwinska-Mrozek, Z., Mrozinski, B., Siwinska, A., & Cichy, W. (2011). Cardiac involvement in children with fatty acid oxidation disorders. Annual Symposium of the Society for the Study of Inborn Errors of Metabolism. https://doi.org/10.1007/s10545-011-9371-z

Kang, E., Kim, Y. M., Kang, M., Heo, S. H., Kim, G. H., Choi, I. H., Choi, J. H., Yoo, H. W., & Lee, B. H. (2018). Clinical and genetic characteristics of patients with fatty acid oxidation disorders identified by newborn screening. BMC Pediatrics, 18(1). https://doi.org/10.1186/S12887-018-1069-Z

Karall, D., Brunner-Krainz, M., Kogelnig, K., Konstantopoulou, V., Maier, E. M., Möslinger, D., Plecko, B., Sperl, W., Volkmar, B., & Scholl-Bürgi, S. (2015). Clinical outcome, biochemical and therapeutic follow-up in 14 Austrian patients with Long-Chain 3-Hydroxy Acyl CoA Dehydrogenase Deficiency (LCHADD). Orphanet Journal of Rare Diseases, 10(1). https://doi.org/10.1186/S13023-015-0236-7

Karall, D., Mair, G., Albrecht, U., Niedermayr, K., Karall, T., Schobersberger, W., & Scholl-Bürgi, S. (2014). Sports in LCHAD deficiency: maximal incremental and endurance exercise tests in a 13-year-old patient with long-chain 3-hydroxy Acyl-CoA dehydrogenase deficiency (LCHADD) and heptanoate treatment. JIMD Reports, 17, 7–12. https://doi.org/10.1007/8904_2014_313

Karall, D., Scholl-Burgi, S., & Angeborene Stoffwechselstorungen, A. G. (2018). The spectrum of clinical phenotypes in fatty acid oxidation disorders. 32nd Annual Meeting Arbeitsgemeinschaft Für Pädiatrische Stoffwechselstörungen.

Kasapkara, Ç. S., Nuoffer, J.-M., Baysoy, G., Aldudak, B., Özbek, M. N., Akcaboy, M., & Largiadèr, C. R. (2019). Lethal Very Long-Chain Acyl-Coa Dehydrogenase Deficiency with A Novel Mutation. Gazi Medical Journal, 30(3). https://doi.org/10.12996/gmj.2019.83

Katz, S., Landau, Y., Pode-Shakked, B., Pessach, I. M., Rubinshtein, M., Anikster, Y., Salem, Y., & Paret, G. (2017). Cardiac failure in very long chain acyl-CoA dehydrogenase deficiency requiring extracorporeal membrane oxygenation (ECMO) treatment: A case report and review of the literature. Molecular Genetics and Metabolism Reports, 10, 5–7. https://doi.org/10.1016/J.YMGMR.2016.11.008

Katzir, Z., Hochman, B., Biro, A., Rubinger, D. I., Feigel, D., Silver, J., Friedlaender, M. M., Popovtzer, M. M., & Smetana, S. (1996). Carnitine palmitoyltransferase deficiency: An underdiagnosed condition? American Journal of Nephrology, 16(2), 162–166. https://doi.org/10.1159/000168991

Kelly, D. P., Mendelsohn, N. J., Sobel, B. E., & Bergmann, S. R. (1993). Detection and assessment by positron emission tomography of a genetically determined defect in myocardial fatty acid utilization (long-chain acyl-coa dehydrogenase deficiency). The American Journal of Cardiology, 71(8), 738–744. https://doi.org/10.1016/0002-9149(93)91020-I

Kim, Y. M., Kim, G., Ko, H., Yoo, H. W., & Lee, H. D. (2018). Treatable massive pericardial effusion and hypertrophic cardiomyopathy in an infant with a novel homozygous ACADVL mutation: A case report. Medicine (United States), 97(20). https://doi.org/10.1097/MD.0000000000010813

Kluge, S., Kühnelt, P., Block, A., Merkel, M., Gocht, A., Lukacs, Z., Kohlschütter, A., & Kreymann, G. (2003). A young woman with persistent hypoglycemia, rhabdomyolysis, and coma: Recognizing fatty acid oxidation defects in adults. Critical Care Medicine, 31(4), 1273–1276. https://doi.org/10.1097/01.CCM.0000045201.10682.F6

Knottnerus, S. J. G., Bleeker, J. C., Ferdinandusse, S., Houtkooper, R. H., Langeveld, M., Nederveen, A. J., Strijkers, G. J., Visser, G., Wanders, R. J. A., Wijburg, F. A., Boekholdt, S. M., & Bakermans, A. J. (2020). Subclinical effects of long-chain fatty acid β-oxidation deficiency on the adult heart: A case-control magnetic resonance study. Journal of Inherited Metabolic Disease, 43(5), 969–980. https://doi.org/10.1002/JIMD.12266

Knottnerus, S. J. G., Bleeker, J. C., Wüst, R. C. I., Ferdinandusse, S., IJlst, L., Wijburg, F. A., Wanders, R. J. A., Visser, G., & Houtkooper, R. H. (2018). Disorders of mitochondrial long-chain fatty acid oxidation and the carnitine shuttle. Reviews in Endocrine and Metabolic Disorders, 19(1), 93–106. https://doi.org/10.1007/S11154-018-9448-1

Knottnerus, S. J. G., Pras-Raves, M. L., van der Ham, M., Ferdinandusse, S., Houtkooper, R. H., Schielen, P. C. J. I., Visser, G., Wijburg, F. A., & de Sain-van der Velden, M. G. M. (2020). Prediction of VLCAD deficiency phenotype by a metabolic fingerprint in newborn screening bloodspots. Biochimica et Biophysica Acta - Molecular Basis of Disease, 1866(6). https://doi.org/10.1016/J.BBADIS.2020.165725

Knowles, J. A., Dimopoulos, I. S., & Macdonald, I. M. (2019). RETINAL PHENOTYPE in A CASE of LCHAD/TFP DEFICIENCY with LATE-STAGE DIAGNOSIS. Retinal Cases and Brief Reports, 13(3), 279–282. https://doi.org/10.1097/ICB.0000000000000579

Ko, J. M., Seo, J., Choi, M., Song, J., Lee, K. A., & Shin, C. H. (2016). Rare Korean Cases of very-long-chain Acyl-CoA dehydrogenase deficiency with a novel recurrent mutation. Annals of Clinical and Laboratory Science, 46(1), 97–101.

Kobayashi, T., Minami, S., Mitani, A., Tanizaki, Y., Booka, M., Okutani, T., Yamaguchi, S., & Ino, K. (2015). Acute fatty liver of pregnancy associated with fetal mitochondrial trifunctional protein deficiency. Journal of Obstetrics and Gynaecology Research, 41(5), 799–802. https://doi.org/10.1111/JOG.12609

Kompare, M., & Rizzo, W. B. (2008). Mitochondrial Fatty-Acid Oxidation Disorders. Seminars in Pediatric Neurology, 15(3), 140–149. https://doi.org/10.1016/J.SPEN.2008.05.008

Korman, S. H., Pitt, J. J., Boneh, A., Dweikat, I., Zater, M., Meiner, V., Gutman, A., & Brivet, M. (2006). A novel SLC25A20 splicing mutation in patients of different ethnic origin with neonatally lethal carnitine-acylcarnitine translocase (CACT) deficiency. Molecular Genetics and Metabolism, 89(4), 332–338. https://doi.org/10.1016/J.YMGME.2006.06.009

Kritzer, A., Tarrant, S., Sussman-Karten, K., & Barbas, K. (2020). Use of skimmed breast milk for an infant with a long-chain fatty acid oxidation disorder: A novel therapeutic intervention. JIMD Reports, 55(1), 44–50. https://doi.org/10.1002/JMD2.12152

Kumar, G., Nair, R., Hendawy, B. S., AlShkeili, O. A., Alabdouli, A. A., Ali, A. M. Al, & AlTenaiji, A. M. J. (2019). Collapsing glomerulopathy in a child with LCHAD deficiency: a rare association. CEN Case Reports, 8(3), 173–177. https://doi.org/10.1007/S13730-019-00387-3

Kwiatkowska, J., Wierzba, J., Karaszewska, A., Kozlowski, D., Sykut-Cegielska, J., & Stanko, A. (2017). Clinical course and cardiovascular outcomes in patients with the long-chain 3-hydroxyacyl-coenzyme A dehydrogenase deficiency. Cardiology Journal, 24(1), 101–104. https://doi.org/10.5603/CJ.2017.0009

Laforêt, P., Acquaviva-Bourdain, C., Rigal, O., Brivet, M., Penisson-Besnier, I., Chabrol, B., Chaigne, D., Boespflug-Tanguy, O., Laroche, C., Bedat-Millet, A. L., Behin, A., Delevaux, I., Lombès, A., Andresen, B. S., Eymard, B., & Vianey-Saban, C. (2009). Diagnostic assessment and long-term follow-up of 13 patients with Very Long-Chain Acyl-Coenzyme A dehydrogenase (VLCAD) deficiency. Neuromuscular Disorders, 19(5), 324–329. https://doi.org/10.1016/J.NMD.2009.02.007

Landau, Y. E., Waisbren, S. E., Chan, L. M. A., & Levy, H. L. (2017). Long-term outcome of expanded newborn screening at Boston children’s hospital: benefits and challenges in defining true disease. Journal of Inherited Metabolic Disease, 40(2), 209–218. https://doi.org/10.1007/S10545-016-0004-4

Lee, H. C. H., Mak, C. M., Lam, C. W., Yuen, Y. P., Chen, A. O. K., Shek, C. C., Siu, T. S., Lai, C. K., Ching, C. K., Siu, W. K., Chen, S. P. L., Law, C. Y., Tai, M. H. L., Tam, S., & Chan, A. Y. W. (2011). Analysis of inborn errors of metabolism: Disease spectrum for expanded newborn screening in Hong Kong. Chinese Medical Journal, 124(7), 983–989. https://doi.org/10.3760/cma.j.issn.0366-6999.2011.07.006

Leslie, N. D., & Saenz-Ayala, S. (1993). Very Long-Chain Acyl-Coenzyme A Dehydrogenase Deficiency. In GeneReviews®.

Li, X., Ding, Y., Ma, Y., Liu, Y., Wang, Q., Song, J., & Yang, Y. (2015). Very long-chain acyl-coenzyme A dehydrogenase deficiency in Chinese patients: Eight case reports, including one case of prenatal diagnosis. European Journal of Medical Genetics, 58(3), 134–139. https://doi.org/10.1016/J.EJMG.2015.01.005

Li, X., Zhao, F., Zhao, Z., Zhao, X., Meng, H., Zhang, D., Zhao, S., & Ding, M. (2022). Neonatal sudden death caused by a novel heterozygous mutation in SLC25A20 gene: A case report and brief literature review. Legal Medicine, 54. https://doi.org/10.1016/J.LEGALMED.2021.101990

Liewluck, T., Mundi, M. S., & Mauermann, M. L. (2013). Mitochondrial trifunctional protein deficiency: a rare cause of adult-onset rhabdomyolysis. Muscle & Nerve, 48(6), 989–991. https://doi.org/10.1002/MUS.23959

Lim, C. C., Vockley, J., Ujah, O., Kirby, R. S., Edick, M. J., Berry, S. A., & Arnold, G. L. (2022). Outcomes and genotype correlations in patients with mitochondrial trifunctional protein or isolated long chain 3-hydroxyacyl-CoA dehydrogenase deficiency enrolled in the IBEM-IS database. Molecular Genetics and Metabolism Reports, 32. https://doi.org/10.1016/J.YMGMR.2022.100884

Lin, J., Boudreault, K., & Tsang, S. (2017). Multimodal imaging and electroretinography in long-chain 3-hydroxyacyl coenzyme a dehydrogenase deficiency. Retinal Cases and Brief Reports, 11(1), S107–S112. https://doi.org/10.1097/ICB.0000000000000428

Lin, W. (2022). Expert consensus on diagnosis and treatment of very long-chain acyl-CoA dehydrogenase deficiency. Zhejiang Da Xue Xue Bao. Yi Xue Ban = Journal of Zhejiang University. Medical Sciences, 51(1), 122–128. https://doi.org/10.3724/ZDXBYXB-2022-0107

Lin, Y., Zhang, W., Chen, D., Lin, C., Zheng, Z., Fu, Q., Li, M., & Peng, W. (2020). Newborn screening and genetic characteristics of patients with short- and very long-chain acyl-CoA dehydrogenase deficiencies. Clinica Chimica Acta, 510, 285–290. https://doi.org/10.1016/J.CCA.2020.07.038

Lin, Y., Zheng, Q., Zheng, T., Zheng, Z., Lin, W., & Fu, Q. (2019). Expanded newborn screening for inherited metabolic disorders and genetic characteristics in a southern Chinese population. Clinica Chimica Acta, 494, 106–111. https://doi.org/10.1016/J.CCA.2019.03.1622

Lindhardt Madsen, K., Preisler, N., Rasmussen, J., Hedermann, G., Olesen, J. H., Lund, A. M., & Vissing, J. (2018). L-carnitine improves skeletal muscle fat oxidation in primary carnitine deficiency. Journal of Clinical Endocrinology and Metabolism, 103(12), 4580–4588. https://doi.org/10.1210/JC.2018-00953

Liu, Z. R., Dong, H. L., Ma, Y., & Wu, Z. Y. (2019). Identification and functional characterization of mutations within HADHB associated with mitochondrial trifunctional protein deficiency. Mitochondrion, 49, 200–205. https://doi.org/10.1016/J.MITO.2019.09.004

Llorca-Cardeñosa, A., Català-Mora, J., García-Cazorla, A., Meavilla, S., & Castejón-Ponce, E. (2016a). Déficit de 3-hidroxiacil-CoA-deshidrogenasa de cadena larga: a propósito de un caso. Archivos de La Sociedad Espanola de Oftalmologia, 91(5), 236–239. https://doi.org/10.1016/J.OFTAL.2016.01.006

Llorca-Cardeñosa, A., Català-Mora, J., García-Cazorla, A., Meavilla, S., & Castejón-Ponce, E. (2016b). Long-chain 3-hydroxyacyl-CoA dehydrogenase deficiency: A case report. Archivos de La Sociedad Española de Oftalmología, 91(5), 236–239. https://doi.org/10.1016/j.oftal.2016.01.006

Longo, N., Amat Di San Filippo, C., & Pasquali, M. (2006). Disorders of carnitine transport and the carnitine cycle. American Journal of Medical Genetics - Seminars in Medical Genetics, 142 C(2), 77–85. https://doi.org/10.1002/AJMG.C.30087

Lopriore, E., Gemke, R. J. B. J., Verhoeven, N. M., Jakobs, C., Wanders, R. J. A., Roeleveld-Versteeg, A. B. C., & Poll-The, B. T. (2001). Carnitine-acylcarnitine translocase deficiency: Phenotype, residual enzyme activity and outcome. European Journal of Pediatrics, 160(2), 101–104. https://doi.org/10.1007/S004310000644

Lotz-Havla, A. S., Röschinger, W., Schiergens, K., Singer, K., Karall, D., Konstantopoulou, V., Wortmann, S. B., & Maier, E. M. (2018). Fatal pitfalls in newborn screening for mitochondrial trifunctional protein (MTP)/long-chain 3-Hydroxyacyl-CoA dehydrogenase (LCHAD) deficiency. Orphanet Journal of Rare Diseases, 13(1). https://doi.org/10.1186/S13023-018-0875-6

Lu, Y., Wu, R., Meng, L., Lv, H., Liu, J., Zuo, Y., Zhang, W., Yuan, Y., & Wang, Z. (2018). HADHB mutations cause infantile-onset axonal Charcot-Marie-Tooth disease: A report of two cases. Clinical Neuropathology, 37(5), 232–238. https://doi.org/10.5414/NP301097

Lund, A. M., Joensen, F., Hougaard, D. M., Jensen, L. K., Christensen, E., Christensen, M., Nørgaard-Petersen, B., Schwartz, M., & Skovby, F. (2007). Carnitine transporter and holocarboxylase synthetase deficiencies in The Faroe Islands. Journal of Inherited Metabolic Disease, 30(3), 341–349. https://doi.org/10.1007/S10545-007-0527-9

Lund, A. M., Skovby, F., Vestergaard, H., Christensen, M., & Christensen, E. (2010). Clinical and biochemical monitoring of patients with fatty acid oxidation disorders. Journal of Inherited Metabolic Disease, 33(5), 495–500. https://doi.org/10.1007/S10545-009-9000-2

Lund, A. M., Wibrand, F., Skogstrand, K., Bækvad-Hansen, M., Gregersen, N., Andresen, B. S., Hougaard, D. M., Dunø, M., & Olsen, R. K. J. (2021). Use of molecular genetic analyses in danish routine newborn screening. International Journal of Neonatal Screening, 7(3). https://doi.org/10.3390/IJNS7030050

Lund, K. C., Scottoline, B., & Jordan, B. K. (2023). Carnitine-Acylcarnitine Translocase Deficiency Masked by Extreme Prematurity. Journal of Pediatric Genetics, 12(02), 179–183. https://doi.org/10.1055/S-0041-1723960

MacDonald, A., Webster, R., Whitlock, M., Gerrard, A., Daly, A., Preece, M. A., Evans, S., Ashmore, C., Chakrapani, A., Vijay, S., & Santra, S. (2018). The safety of Lipistart, a medium-chain triglyceride based formula, in the dietary treatment of long-chain fatty acid disorders: A phase i study. Journal of Pediatric Endocrinology and Metabolism, 31(3), 297–304. https://doi.org/10.1515/JPEM-2017-0426

Madden, M. C., Wołkowicz, P. E., Pohost, G. M., McMillin, J. B., & Pike, M. M. (1995). Acylcarnitine accumulation does not correlate with reperfusion recovery in palmitate-perfused rat hearts. The American Journal of Physiology, 268(6 Pt 2), H2505-12. https://doi.org/10.1152/ajpheart.1995.268.6.H2505

Mador‐house, R., Liu, Z., & Dyack, S. (2021). Detection of early onset carnitine palmitoyltransferase ii deficiency by newborn screening. Should cpt ii deficiency be a primary disease target? International Journal of Neonatal Screening, 7(3). https://doi.org/10.3390/IJNS7030055

Madsen, K. L., Stemmerik, M. G., Buch, A. E., Poulsen, N. S., Vissing, J., Lund, A. M., & Lund, A. M. (2019). Impaired Fat Oxidation during Exercise in Long-Chain Acyl-CoA Dehydrogenase Deficiency Patients and Effect of IV-Glucose. Journal of Clinical Endocrinology and Metabolism, 104(9), 3610–3613. https://doi.org/10.1210/JC.2019-00453

Magoulas, P. L., & El-Hattab, A. W. (2012). Systemic primary carnitine deficiency: An overview of clinical manifestations, diagnosis, and management. Orphanet Journal of Rare Diseases, 7(1). https://doi.org/10.1186/1750-1172-7-68

Maguolo, A., Rodella, G., Dianin, A., Nurti, R., Monge, I., Rigotti, E., Cantalupo, G., Salviati, L., Tucci, S., Pellegrini, F., Molinaro, G., Lupi, F., Tonin, P., Pasini, A., Campostrini, N., Ion Popa, F., Teofoli, F., Vincenzi, M., Camilot, M., … Bordugo, A. (2020). Diagnosis, genetic characterization and clinical follow up of mitochondrial fatty acid oxidation disorders in the new era of expanded newborn screening: A single centre experience. Molecular Genetics and Metabolism Reports, 24. https://doi.org/10.1016/J.YMGMR.2020.100632

Mahapatra, S., Ananth, A., Baugh, N., Damian, M., & Enns, G. M. (2018). Triheptanoin: A rescue therapy for cardiogenic shock in carnitine-acylcarnitine translocase deficiency. JIMD Reports, 39, 19–23. https://doi.org/10.1007/8904_2017_36

Marques, V., Tomaselli, P., Sobreira, C., Barreira, A., & Marques, W. (2018). Early onset neuropathy with episodes of worsening and brown urine: Mitochondrial trifunctional protein (MTP) deficiency. Journal of the Peripheral Nervous System, 23(4), 368. https://doi.org/10.1111/jns.12290

Marsden, D. (2003). Expanded newborn screening by tandem mass spectrometry: the Massachusetts and New England experience. The Southeast Asian Journal of Tropical Medicine and Public Health, 34 Suppl 3, 111–114.

Marsden, D., Bedrosian, C. L., & Vockley, J. (2021). Impact of newborn screening on the reported incidence and clinical outcomes associated with medium- and long-chain fatty acid oxidation disorders. Genetics in Medicine, 23(5), 816–829. https://doi.org/10.1038/S41436-020-01070-0

Martin, J. M., Gillingham, M. B., & Harding, C. O. (2014). Use of propofol for short duration procedures in children with long chain 3-hydroxyacyl-CoA dehydrogenase (LCHAD) or trifunctional protein (TFP) deficiencies. Molecular Genetics and Metabolism, 112(2), 139–142. https://doi.org/10.1016/J.YMGME.2014.03.012

Matern, D., Strauss, A. W., Hillman, S. L., Mayatepek, E., Millington, D. S., & Trefz, F. K. (1999). Diagnosis of mitochondrial trifunctional protein deficiency in a blood spot from the newborn screening card by tandem mass spectrometry and DNA analysis. Pediatric Research, 46(1), 45–49. https://doi.org/10.1203/00006450-199907000-00008

Mathur, A., Sims, H. F., Gopalakrishnan, D., Gibson, B., Rinaldo, P., Vockley, J., Hug, G., & Strauss, A. W. (1999). Molecular heterogeneity in very-long-chain acyl-CoA dehydrogenase deficiency causing pediatric cardiomyopathy and sudden death. Circulation, 99(10), 1337–1343. https://doi.org/10.1161/01.CIR.99.10.1337

Mathur, A., Sims, H. F., Powell, C. K., & Strauss, A. W. (1996). Infantile cardiomyopathy - A common clinical manifestation of very long chain acyl-coA dehydrogenase deficiency. American Heart Association 69th Scientific Sessions.

Mendez-Figueroa, H., Shchelochkov, O. A., Shaibani, A., Aagaard-Tillery, K., & Shinawi, M. S. (2010). Clinical and biochemical improvement of very long-chain acyl-CoA dehydrogenase deficiency in pregnancy. Journal of Perinatology : Official Journal of the California Perinatal Association, 30(8), 558–562. https://doi.org/10.1038/jp.2009.198

Merinero, B., Alcaide, P., Martín-Hernández, E., Morais, A., García-Silva, M. T., Quijada-Fraile, P., Pedrón-Giner, C., Dulin, E., Yahyaoui, R., Egea, J. M., Belanger-Quintana, A., Blasco-Alonso, J., Fernandez Ruano, M. L., Besga, B., Ferrer-López, I., Leal, F., Ugarte, M., Ruiz-Sala, P., Pérez, B., & Pérez-Cerdá, C. (2018). Four years’ experience in the diagnosis of very long-chain acyl-coa dehydrogenase deficiency in infants detected in three spanish newborn screening centers. JIMD Reports, 39, 63–74. https://doi.org/10.1007/8904_2017_40

Merinero, B., Pascual Pascual, S. I., Pérez-Cerdá, C., Gangoiti, J., Castro, M., Garcia, M. J., Pascual Castroviejo, I., Vianey-Saban, C., Andresen, B., Gregersen, N., & Ugarte, M. (1999). Adolescent myopathic presentation in two sisters with very long-chain acyl-CoA dehydrogenase deficiency. Journal of Inherited Metabolic Disease, 22(7), 802–810. https://doi.org/10.1023/A:1005553907216

Merinero, B., Pérez-Cerdá, C., Garcia, M. J., Gangoiti, J., Font, L. M., Silva, M. T. G., Vianey-Saban, C., Duran, M., & Ugarte, M. (1996). Mitochondrial very long-chain acyl-CoA dehydrogenase deficiency with a mild clinical course. Journal of Inherited Metabolic Disease, 19(2), 173–176. https://doi.org/10.1007/BF01799422

Merritt, J. L., MacLeod, E., Jurecka, A., & Hainline, B. (2020). Clinical manifestations and management of fatty acid oxidation disorders. Reviews in Endocrine and Metabolic Disorders, 21(4), 479–493. https://doi.org/10.1007/S11154-020-09568-3

Merritt, J. L., Vedal, S., Abdenur, J. E., Au, S. M., Barshop, B. A., Feuchtbaum, L., Harding, C. O., Hermerath, C., Lorey, F., Sesser, D. E., Thompson, J. D., & Yu, A. (2014). Infants suspected to have very-long chain acyl-CoA dehydrogenase deficiency from newborn screening. Molecular Genetics and Metabolism, 111(4), 484–492. https://doi.org/10.1016/J.YMGME.2014.01.009

Miller, M. J., Burrage, L. C., Gibson, J. B., Strenk, M. E., Lose, E. J., Bick, D. P., Elsea, S. H., Sutton, V. R., Sun, Q., Graham, B. H., Craigen, W. J., Zhang, V. W., & Wong, L. J. C. (2015). Recurrent ACADVL molecular findings in individuals with a positive newborn screen for very long chain acyl-coA dehydrogenase (VLCAD) deficiency in the United States. Molecular Genetics and Metabolism, 116(3), 139–145. https://doi.org/10.1016/J.YMGME.2015.08.011

Miyajima, H., Orii, K. E., Shindo, Y., Hashimoto, T., Shinka, T., Kuhara, T., Matsumoto, I., Shimizu, H., & Kaneko, E. (1997). Mitochondrial trifunctional protein deficiency associated with recurrent myoglobinuria in adolescence. Neurology, 49(3), 833–837. https://doi.org/10.1212/WNL.49.3.833

Modena, M., Castiglione, V., Aretini, P., Mazzanti, C. M., Chiti, E., Giannoni, A., Emdin, M., & Di Paolo, M. (2020). Unveiling a sudden unexplained death case by whole exome sequencing and bioinformatic analysis. Molecular Genetics and Genomic Medicine, 8(4). https://doi.org/10.1002/MGG3.1182

Mohsen, A. W., Karunanidhi, A., Seminotti, B., Leipnitz, G., Kochersperger, C., Grings, M., El-Gharbawy, A., Ghaloul-Gonzalez, L., Wipf, P., & Vockley, J. (2018). Novel drug therapies of fatty acid beta-oxidation disorders: The future focus and hope. Annual Meeting of the Society for Inherited Metabolic Disorders.

Molenaar, D. S. M., Wanders, R. J. A., Duran, M., Barth P G, & Poll-The, B. T. (2002). Mitochondrial trifunctional protein deficiency mimicking early onset hereditary motor and sensory neuropathy (HMSN) type II. 7th International Congress of the World Muscle Society.

Moore, R., Glasgow, J. F. T., Bingham, M. A., Dodge, J. A., Pollitt, R. J., Olpin, S. E., Middleton, B., & Carpenter, K. (1993). Long-chain 3-hydroxyacyl-coenzyme A dehydrogenase deficiency-diagnosis, plasma carnitine fractions and management in a further patient. European Journal of Pediatrics, 152(5), 433–436. https://doi.org/10.1007/BF01955905

Morales Corado, J. A., Lee, C. U., & Enns, G. M. (1993). Carnitine-Acylcarnitine Translocase Deficiency. GeneReviews®. http://www.ncbi.nlm.nih.gov/pubmed/12859414

Mozrzymas, R., Konikowska, K., & Regulska-Ilow, B. (2017). Energy exchangers with LCT as a precision methodfor diet control in LCHADD. Advances in Clinical and Experimental Medicine, 26(3), 515–525. https://doi.org/10.17219/ACEM/62132

Murata, K. ya, Sugie, H., Nishino, I., Kondo, T., & Ito, H. (2014). A primigravida with very-long-chain acyl-CoA dehydrogenase deficiency. Muscle and Nerve, 49(2), 295–296. https://doi.org/10.1002/MUS.24055

Musumeci, O., Ferlazzo, E., Rodolico, C., Gambardella, A., Gagliardi, M., Aguglia, U., & Toscano, A. (2020). A Family With a Complex Phenotype Caused by Two Different Rare Metabolic Disorders: GLUT1 and Very-Long-Chain Fatty Acid Dehydrogenase (VLCAD) Deficiencies. Frontiers in Neurology, 11. https://doi.org/10.3389/FNEUR.2020.00514

Nadjar, Y., Souvannanorath, S., Maisonobe, T., Brisset, M., De Lonlay, P., Schiff, M., Viala, K., Boutron, A., Nicolas, G., & Laforêt, P. (2020). Sensory neuronopathy as a major clinical feature of mitochondrial trifunctional protein deficiency in adults. Revue Neurologique, 176(5), 380–386. https://doi.org/10.1016/J.NEUROL.2019.11.011

Nasser, M., Javaheri, H., Fedorowicz, Z., & Noorani, Z. (2009). Carnitine supplementation for inborn errors of metabolism. Cochrane Database of Systematic Reviews, 15(2), CD006659. https://doi.org/10.1002/14651858.CD006659.pub2

Nasser, M., Javaheri, H., Fedorowicz, Z., & Noorani, Z. (2012). Carnitine supplementation for inborn errors of metabolism. The Cochrane Database of Systematic Reviews, 2012(2), CD006659. https://doi.org/10.1002/14651858.CD006659.pub3

Niezen-Koning, K. E., van Spronsen, F. J., Ijlst, L., Wanders, R. J. A., Brivet, M., Duran, M., Reijngoud, D. J., Heymans, H. S. A., & Smit, G. P. A. (1995). A patient with lethal cardiomyopathy and a carnitine - acylcarnitine translocase deficiency. Journal of Inherited Metabolic Disease, 18(2), 230–232. https://doi.org/10.1007/BF00711775

Norris, M. K., Scott, A. I., Sullivan, S., Chang, I. J., Lam, C., Sun, A., Hahn, S., Thies, J. M., Gunnarson, M., McKean, K. N., & Merritt, J. L. (2021). Tutorial: Triheptanoin and Nutrition Management for Treatment of Long-Chain Fatty Acid Oxidation Disorders. Journal of Parenteral and Enteral Nutrition, 45(2), 230–238. https://doi.org/10.1002/JPEN.2034

Nuoffer, J. M., De Lonlay, P., Saudubray, J. M., Costa, C., Brivet, M., Roe, C. R., & Chamoles, N. (2000). Familial neonatal SIDS revealing carnitine-acylcarnitine translocase deficiency. European Journal of Pediatrics, 159(1–2), 82–85. https://doi.org/10.1007/PL00013810

Obaid, A., Nashabat, M., Alfadhel, M., Alasmari, A., Al Mutairi, F., Alswaid, A., Faqeih, E., Mushiba, A., Albanyan, M., Alalwan, M., Marsden, D., & Eyaid, W. (2018). Clinical, biochemical, and molecular features in 37 saudi patients with very long chain acyl CoA dehydrogenase deficiency. JIMD Reports, 40, 47–53. https://doi.org/10.1007/8904_2017_58

Ojala, T., Nupponen, I., Saloranta, C., Sarkola, T., Sekar, P., Breilin, A., & Tyni, T. (2015). Fetal left ventricular noncompaction cardiomyopathy and fatal outcome due to complete deficiency of mitochondrial trifunctional protein. European Journal of Pediatrics, 174(12), 1689–1692. https://doi.org/10.1007/S00431-015-2574-9

Oliveira, B. M., Poswar, F. O., Sanseverino, M. T. V, Souza, C. F. M., & Schwartz, I. V. D. (2019). Fatty acid oxidation disorders: A multicentric case series study from Southern Brazil. Annual Symposium of the Society for the Study of Inborn Errors of Metabolism.

Oliveira, S. F., Pinho, L., Rocha, H., Nogueira, C., Vilarinho, L., Dinis, M. J., & Silva, C. (2013). Rhabdomyolysis as a Presenting Manifestation of Very Long-Chain Acyl-Coenzyme a Dehydrogenase Deficiency. Clinics and Practice, 3(2), e22. https://doi.org/10.4081/CP.2013.E22

Olpin, S. E. (2004). Implications of impaired ketogenesis in fatty acid oxidation disorders. Prostaglandins, Leukotrienes and Essential Fatty Acids, 70(3), 293–308. https://doi.org/10.1016/j.plefa.2003.06.003

Olpin, S. E. (2005). Fatty acid oxidation defects as a cause of neuromyopathic disease in infants and adults. Clinical Laboratory, 51(5–6), 289–306.

Olpin, S. E. (2013). Pathophysiology of fatty acid oxidation disorders and resultant phenotypic variability. Journal of Inherited Metabolic Disease, 36(4), 645–658. https://doi.org/10.1007/s10545-013-9611-5

Olpin, S. E., Clark, S., Andresen, B. S., Bischoff, C., Olsen, R. K. J., Gregersen, N., Chakrapani, A., Downing, M., Manning, N. J., Sharrard, M., Bonham, J. R., Muntoni, F., Turnbull, D. N., & Pourfarzam, M. (2005). Biochemical, clinical and molecular findings in LCHAD and general mitochondrial trifunctional protein deficiency. Journal of Inherited Metabolic Disease, 28(4), 533–544. https://doi.org/10.1007/S10545-005-0533-8

Ørngreen, M. C., & Vissing, J. (2017). Treatment Opportunities in Patients With Metabolic Myopathies. Current Treatment Options in Neurology, 19(11). https://doi.org/10.1007/S11940-017-0473-2

Ørstavik, K., Arntzen, K. A., Mathisen, P., Backe, P. H., Tangeraas, T., Rasmussen, M., Kristensen, E., Van Ghelue, M., Jonsrud, C., & Bliksrud, Y. T. (2022). Novel mutations in the HADHB gene causing a mild phenotype of mitochondrial trifunctional protein (MTP) deficiency. JIMD Reports, 63(3), 193–198. https://doi.org/10.1002/JMD2.12276

Osorio, A. N. N., Ortez, C. O., Gutierrez, A. G., Jimenez-Mallebrera, C. J. M., Villoria, J. G. V. G., Ribes, T. R., Vilaseca, M. V., & Colomer, J. C. (2010). Early and severe axonal motor neuropathy and cardiomyopathy in a patient with mitochondrial trifunctional protein deficiency. Neuromuscular Disorders, 20(9–10), 675. https://doi.org/10.1016/j.nmd.2010.07.252

Pande, S. V. (1999). Carnitine-Acylcarnitine Translocase Deficiency. The American Journal of the Medical Sciences, 318(1), 22. https://doi.org/10.1097/00000441-199907000-00004

Pannier, A. (2020). Long-Chain Fatty Acid Oxidation Disorders: Managed Care and Specialty Pharmacy Implications. American Journal of Managed Care, 26, S155–S161. https://doi.org/10.37765/AJMC.2020.88479

Parimbelli, M., Pezzotti, E., Negro, M., Calanni, L., Allemano, S., Bernardi, M., Berardinelli, A., & D’Antona, G. (2021). Nutrition and Exercise in a Case of Carnitine Palmitoyl-Transferase II Deficiency. Frontiers in Physiology, 12. https://doi.org/10.3389/FPHYS.2021.637406

Parini, R., Garavaglia, B., Saudubray, J. M., Bardelli, P., Melotti, D., Zecca, G., & Di Donato, S. (1991). Clinical diagnosis of long-chain acyl-coenzyme A-dehydrogenase deficiency: Use of stress and fat-loading tests. The Journal of Pediatrics, 119(1 PART 1), 77–80. https://doi.org/10.1016/S0022-3476(05)81044-3

Parini, R., Invernizzi, F., Menni, F., Garavaglia, B., Melotti, D., Rimoldi, M., Salera, S., Tosetto, C., & Taroni, F. (1999). Medium‐chain triglyceride loading test in carnitine–acylcarnitine translocase deficiency: Insights on treatment. Journal of Inherited Metabolic Disease, 22(6), 733–739. https://doi.org/10.1023/A:1005548201355

Parini, R., Menni, F., Garavaglia, B., Fesslova, V., Melotti, D., Massone, M. L., Lamantea, E., Rimoldi, M., Vizziello, P., & Gatti, R. (1998). Acute, severe cardiomyopathy as main symptom of late-onset very long- chain acyl-coenzyme A dehydrogenase deficiency. European Journal of Pediatrics, 157(12), 992–995. https://doi.org/10.1007/S004310050984

Park, H. D., Kim, S. R., Ki, C. S., Lee, S. Y., Chang, Y. S., Jin, D. K., & Park, W. S. (2009). Case report: Two novel HADHB gene mutations in a Korean patient with mitochondrial trifunctional protein deficiency. Annals of Clinical and Laboratory Science, 39(4), 399–404.

Pena, L. D. M., van Calcar, S. C., Hansen, J., Edick, M. J., Walsh Vockley, C., Leslie, N., Cameron, C., Mohsen, A. W., Berry, S. A., Arnold, G. L., & Vockley, J. (2016). Outcomes and genotype-phenotype correlations in 52 individuals with VLCAD deficiency diagnosed by NBS and enrolled in the IBEM-IS database. Molecular Genetics and Metabolism, 118(4), 272–281. https://doi.org/10.1016/J.YMGME.2016.05.007

Pervaiz, M. A., Kendal, F., Hegde, M., & Singh, R. H. (2011). MCT oil-based diet reverses hypertrophic cardiomyopathy in a patient with very long chain acyl-coA dehydrogenase deficiency. Indian Journal of Human Genetics, 17(1), 29–32. https://doi.org/10.4103/0971-6866.82190

Pichler, K., Michel, M., Zlamy, M., Scholl-Buergi, S., Ralser, E., Jörg-Streller, M., & Karall, D. (2017). Breast milk feeding in infants with inherited metabolic disorders other than phenylketonuria- A 10-year single-center experience. Journal of Perinatal Medicine, 45(3), 375–382. https://doi.org/10.1515/JPM-2016-0205

Pierre, G., Macdonald, A., Gray, G., Hendriksz, C., Preece, M. A., & Chakrapani, A. (2007). Prospective treatment in carnitine-acylcarnitine translocase deficiency. Journal of Inherited Metabolic Disease, 30(5), 815. https://doi.org/10.1007/S10545-007-0518-X

Pitcleathy, R., Sulaiman, R., Olpin, S., Hiwot, T., Cole, D., Rahman, S., Lachmann, R., & Murphy, E. (2011). Fatty acid oxidation disorders in adults: A multicentre UK review of 34 patients. Annual Symposium of the Society for the Study of Inborn Errors of Metabolism.

Pollitt, R. J. (1995). Disorders of mitochondrial long-chain fatty acid oxidation. Journal of Inherited Metabolic Disease, 18(4), 473–490. https://doi.org/10.1007/BF00710058

Pons, R., Cavadini, P., Baratta, S., Invernizzi, F., Lamantea, E., Garavaglia, B., & Taroni, F. (2000). Clinical and molecular heterogeneity in very-long-chain acyl-coenzyme A dehydrogenase deficiency. Pediatric Neurology, 22(2), 98–105. https://doi.org/10.1016/S0887-8994(99)00132-0

Pons, R., Roig, M., Riudor, E., Ribes, A., Briones, P., Ortigosa, L., Baldellou, A., Gil-Gibernau, J., Olesti, M., Navarro, C., & Wanders, R. J. A. (1996). The clinical spectrum of long-chain 3-hydroxyacyl-CoA dehydrogenase deficiency. Pediatric Neurology, 14(3), 236–243. https://doi.org/10.1016/0887-8994(96)00021-5

Potter, B. K., Little, J., Chakraborty, P., Kronick, J. B., Evans, J., Frei, J., Sutherland, S. C., Wilson, K., & Wilson, B. J. (2012). Variability in the clinical management of fatty acid oxidation disorders: Results of a survey of Canadian metabolic physicians. Journal of Inherited Metabolic Disease, 35(1), 115–123. https://doi.org/10.1007/S10545-011-9352-2

Prasad, C., Johnson, J. P., Bonnefont, J. P., Dilling, L. A., Innes, A. M., Haworth, J. C., Beischel, L., Thuillier, L., Prip-Buus, C., Singal, R., Thompson, J. R. G., Prasad, A. N., Buist, N., & Greenberg, C. R. (2001). Hepatic carnitine palmitoyl transferase 1 (CPT1 A) deficiency in North American Hutterites (Canadian and American): Evidence for a founder effect and results of a pilot study on a DNA-based newborn screening program. Molecular Genetics and Metabolism, 73(1), 55–63. https://doi.org/10.1006/MGME.2001.3149

Prasun, P., LoPiccolo, M. K., & Ginevic, I. (1993). Long-Chain Hydroxyacyl-CoA Dehydrogenase Deficiency / Trifunctional Protein Deficiency. In GeneReviews®.

Preisler, N. (2014). Contribution of exercise tests to study treatment and the phenotypes of metabolic myopathies. 13th International Congress on Neuromuscular Diseases.

Prip-Buus, C., Thuillier, L., Abadi, N., Prasad, C., Dilling, L., Klasing, J., Demaugre, F., Greenberg, C. R., Haworth, J. C., Droin, V., Kadhom, N., Gobin, S., Kamoun, P., Girard, J., & Bonnefont, J. P. (2001). Molecular and enzymatic characterization of a unique carnitine palmitoyltransferase 1A mutation in the Hutterite community. Molecular Genetics and Metabolism, 73(1), 46–54. https://doi.org/10.1006/MGME.2001.3176

Przyrembel, H., Jakobs, C., Ijlst, L., de Klerk, J. B. C., & Wanders, R. J. A. (1991). Long‐chain 3‐hydroxyacyl‐CoA dehydrogenase deficiency. Journal of Inherited Metabolic Disease, 14(5), 674–680. https://doi.org/10.1007/BF01799932

Purevsuren, J., Fukao, T., Hasegawa, Y., Kobayashi, H., Li, H., Mushimoto, Y., Fukuda, S., & Yamaguchi, S. (2009). Clinical and molecular aspects of Japanese patients with mitochondrial trifunctional protein deficiency. Molecular Genetics and Metabolism, 98(4), 372–377. https://doi.org/10.1016/j.ymgme.2009.07.011

Qatomah, A., Bukhari, M., Cupler, E., Alardati, H., & Mawardi, M. (2021). Acute reversible rhabdomyolysis during direct-acting antiviral hepatitis C virus treatment: a case report. Journal of Medical Case Reports, 15(1). https://doi.org/10.1186/S13256-021-03138-0

Quinn, J., Georgiadis, A., Lewis, H. B., & Jurecki, E. (2022). Measuring Burden of Illness in Phenylketonuria (PKU): Development of the PKU Symptom Severity and Impacts Scale as a Robust Patient-Reported Outcome. Advances in Therapy, 39(2), 971–991. https://doi.org/10.1007/s12325-021-01986-2

Ramanathan, R., & Ibdah, J. A. (2022). Mitochondrial Dysfunction and Acute Fatty Liver of Pregnancy. International Journal of Molecular Sciences, 23(7). https://doi.org/10.3390/IJMS23073595

Ramsey, P. S., & Biggio, J. R. (2005). Carnitine palmitoyltransferase deficiency in pregnancy. The Journal of Maternal-Fetal & Neonatal Medicine, 18(5), 357–359. https://doi.org/10.1080/14767050500275861

Rector, R. S., & Ibdah, J. A. (2010). Fatty acid oxidation disorders: maternal health and neonatal outcomes. Seminars in Fetal and Neonatal Medicine, 15(3), 122–128. https://doi.org/10.1016/J.SINY.2009.10.006

Rector, R. S., Payne, R. M., & Ibdah, J. A. (2008). Mitochondrial trifunctional protein defects: Clinical implications and therapeutic approaches. Advanced Drug Delivery Reviews, 60(13–14), 1488–1496. https://doi.org/10.1016/J.ADDR.2008.04.014

Remec, Z. I., Groselj, U., Drole Torkar, A., Zerjav Tansek, M., Cuk, V., Perko, D., Ulaga, B., Lipovec, N., Debeljak, M., Kovac, J., Battelino, T., & Repic Lampret, B. (2021). Very Long-Chain Acyl-CoA Dehydrogenase Deficiency: High Incidence of Detected Patients With Expanded Newborn Screening Program. Frontiers in Genetics, 12. https://doi.org/10.3389/FGENE.2021.648493

Rinaldo, P., Stanley, C. A., Hsu, B. Y. L., Sanchez, L. A., & Stern, H. J. (1997). Sudden neonatal death in carnitine transporter deficiency. Journal of Pediatrics, 131(2), 304–305. https://doi.org/10.1016/S0022-3476(97)70171-9

Rocchiccioli, F., Wanders, R. J. A., Aubourg, P., Vianey-Liaud, C., Ijlst, L., Fabre, M., Cartier, N., & Bougneres, P. F. (1990). Deficiency of long-chain 3-hydroxyacyl-CoA dehydrogenase: A cause of lethal myopathy and cardiomyopathy in early childhood. Pediatric Research, 28(6), 657–662. https://doi.org/10.1203/00006450-199012000-00023

Roe, C. R., & Brunengraber, H. (2015). Anaplerotic treatment of long-chain fat oxidation disorders with triheptanoin: Review of 15years Experience. Molecular Genetics and Metabolism, 116(4), 260–268. https://doi.org/10.1016/j.ymgme.2015.10.005

Roe, C. R., & Mochel, F. (2006). Anaplerotic diet therapy in inherited metabolic disease: therapeutic potential. Journal of Inherited Metabolic Disease, 29(2–3), 332–340. https://doi.org/10.1007/s10545-006-0290-3

Roe, C. R., Sweetman, L., Roe, D. S., David, F., & Brunengraber, H. (2002). Treatment of cardiomyopathy and rhabdomyolysis in long-chain fat oxidation disorders using an anaplerotic odd-chain triglyceride. Journal of Clinical Investigation, 110(2), 259–269. https://doi.org/10.1172/JCI15311

Roe, C. R., Wiltse, H. E., Sweetman, L., & Alvarado, L. L. (2000). Death caused by perioperative fasting and sedation in a child with unrecognized very long chain acyl-coenzyme A dehydrogenase deficiency. Journal of Pediatrics, 136(3), 397–399. https://doi.org/10.1067/MPD.2000.103853

Roe, C. R., Yang, B. Z., Brunengraber, H., Roe, D. S., Wallace, M., & Garritson, B. K. (2008). Carnitine palmitoyltransferase II deficiency: Successful anaplerotic diet therapy. Neurology, 71(4), 260–264. https://doi.org/10.1212/01.WNL.0000318283.42961.E9

Roe, D. S., Vianey-Saban, C., Sharma, S., Zabot, M. T., & Roe, C. R. (2001). Oxidation of unsaturated fatty acids by human fibroblasts with very-long-chain acyl-CoA dehydrogenase deficiency: Aspects of substrate specificity and correlation with clinical phenotype. Clinica Chimica Acta, 312(1–2), 55–67. https://doi.org/10.1016/S0009-8981(01)00594-0

Rohr, F. (2015). Nutrition management of fatty acid oxidation disorders, . In L. E. Bernstein, F. Rhor, & J. R. Helm (Eds.), Nutrition Management of Inherited Metabolic Diseases: Lessons from Metabolic University (pp. 271–282). Springer International Publishing.

Röschinger, W., Muntau, A. C., Duran, M., Dorland, L., IJlst, L., Wanders, R. J. A., & Roscher, A. A. (2000). Carnitine-acylcarnitine translocase deficiency: Metabolic consequences of an impaired mitochondrial carnitine cycle. Clinica Chimica Acta, 298(1–2), 55–68. https://doi.org/10.1016/S0009-8981(00)00268-0

Rossi, A., Hoogeveen, I. J., Lubout, C. M. A., de Boer, F., Fokkert-Wilts, M. J., Rodenburg, I. L., van Dam, E., Grünert, S. C., Martinelli, D., Scarpa, M., Dekker, H., te Boekhorst, S. T., van Spronsen, F. J., Derks, T. G. J., de Baere, L., Bellettato, C., Bosch, A. M., Sallago, J. B., Botto, L. D., … Ziagaki, A. (2021). A generic emergency protocol for patients with inborn errors of metabolism causing fasting intolerance: A retrospective, single-center study and the generation of www.emergencyprotocol.net. Journal of Inherited Metabolic Disease, 44(5), 1124–1135. https://doi.org/10.1002/JIMD.12386

Rovelli, V., Manzoni, F., Viau, K., Pasquali, M., & Longo, N. (2019). Clinical and biochemical outcome of patients with very long-chain acyl-CoA dehydrogenase deficiency. Molecular Genetics and Metabolism, 127(1), 64–73. https://doi.org/10.1016/J.YMGME.2019.04.001

Rubio-Gozalbo, M. E., Bakker, J. A., Waterham, H. R., & Wanders, R. J. A. (2004). Carnitine-acylcarnitine translocase deficiency, clinical, biochemical and genetic aspects. Molecular Aspects of Medicine, 25(5–6), 521–532. https://doi.org/10.1016/J.MAM.2004.06.007

Rücklová, K., Hrubá, E., Pavlíková, M., Hanák, P., Farolfi, M., Chrastina, P., Vlášková, H., Kousal, B., Smolka, V., Foltenová, H., Adam, T., Friedecký, D., Ješina, P., Zeman, J., Kožich, V., & Honzík, T. (2021). Impact of newborn screening and early dietary management on clinical outcome of patients with long chain 3-hydroxyacyl-coa dehydrogenase deficiency and medium chain acyl-coa dehydrogenase deficiency—a retrospective nationwide study. Nutrients, 13(9). https://doi.org/10.3390/nu13092925

Ruiz-Sanz, J. I., Aldamiz-Echevarria, L., Arrizabalaga, J., Aquino, L., Jimeno, P., Pérez-Nanclares, G., & Sanjurjo, P. (2001). Polyunsaturated fatty acid deficiency during dietary treatment of very long-chain acyl-CoA dehydrogenase deficiency. Rescue with soybean oil. Journal of Inherited Metabolic Disease, 24(4), 493–503. https://doi.org/10.1023/A:1010533730659

Ryder, B., Inbar-Feigenberg, M., Glamuzina, E., Halligan, R., Vara, R., Elliot, A., Coman, D., Minto, T., Lewis, K., Schiff, M., Vijay, S., Akroyd, R., Thompson, S., MacDonald, A., Woodward, A. J. M., Gribben, J. E. L., Grunewald, S., Belaramani, K., Hall, M., … Bhattacharya, K. (2021). New insights into carnitine-acylcarnitine translocase deficiency from 23 cases: Management challenges and potential therapeutic approaches. Journal of Inherited Metabolic Disease, 44(4), 903–915. https://doi.org/10.1002/JIMD.12371

Ryder, B., Knoll, D., Love, D. R., Shepherd, P., Love, J. M., Reed, P. W., de Hora, M., Webster, D., Glamuzina, E., & Wilson, C. (2016). The natural history of elevated tetradecenoyl-L-carnitine detected by newborn screening in New Zealand: implications for very long chain acyl-CoA dehydrogenase deficiency screening and treatment. Journal of Inherited Metabolic Disease, 39(3), 409–414. https://doi.org/10.1007/S10545-015-9911-Z

Ryder, B., Lewis, K., Akroyd, R., Thompson, S., Coman, D., Glamuzina, E., Inbar-Feigenberg, M., Schiff, M., Wilson, C., & Bhattacharya, K. (2018). Neonatal carnitine acylcarnitine translocase deficiency: 8 new cases with survival in 6. Annual Symposium of the Society for the Study of Inborn Errors of Metabolism.

Sahai, I., Bailey, J. C., Eaton, R. B., Zytkovicz, T., & Harris, D. J. (2011). A near-miss: Very long chain acyl-CoA dehydrogenase deficiency with normal primary markers in the initial well-timed newborn screening specimen. Journal of Pediatrics, 158(1), 172. https://doi.org/10.1016/J.JPEDS.2010.09.026

Saudubray, J. M., Martin, D., De Lonlay, P., Touati, G., Poggi‐Travert, F., Bonnet, D., Jouvet, P., Boutron, M., Slama, A., Vianey‐Saban, C., Bonnefont, J. P., Rabier, D., Kamoun, P., & Brivet, M. (1999). Recognition and management of fatty acid oxidation defects: A series of 107 patients. Journal of Inherited Metabolic Disease, 22(4), 487–502. https://doi.org/10.1023/A:1005556207210

Saudubray, J. M., Sedel, F., & Walter, J. H. (2006). Clinical approach to treatable inborn metabolic diseases: An introduction. Journal of Inherited Metabolic Disease, 29(2–3), 261–274. https://doi.org/10.1007/S10545-006-0358-0

Scalais, E., Bottu, J., Wanders, R. J. A., Ferdinandusse, S., Waterham, H. R., & De Meirleir, L. (2015). Familial very long chain acyl-CoA dehydrogenase deficiency as a cause of neonatal sudden infant death: Improved survival by prompt diagnosis. American Journal of Medical Genetics, Part A, 167(1), 211–214. https://doi.org/10.1002/AJMG.A.36803

Schiff, M., Mohsen, A. W., Karunanidhi, A., McCracken, E., Yeasted, R., & Vockley, J. (2013). Molecular and cellular pathology of very-long-chain acyl-CoA dehydrogenase deficiency. Molecular Genetics and Metabolism, 109(1), 21–27. https://doi.org/10.1016/j.ymgme.2013.02.002

Scholte, H. R., Van Coster, R. N. A., De Jonge, P. C., Poorthuis, B. J. H. M., Jeneson, J. A. L., Andresen, B. S., Gregersen, N., De Klerk, J. B. C., & Busch, H. F. M. (1999). Myopathy in very-long-chain acyl-CoA dehydrogenase deficiency: Clinical and biochemical differences with the fatal cardiac phenotype. Neuromuscular Disorders, 9(5), 313–319. https://doi.org/10.1016/S0960-8966(99)00032-2

Schrader, K. A., Sinclair, G. B., McLeod, K., & Sirrs, S. (2011). Optimization of pre-exercise medium-chain triglycerides supplementation by real-time monitoring in patients with LCHAD. Journal of Inherited Metabolic Disease, 34, S262. https://doi.org/10.1007/s10545-011-9371-z

Schrijver-Wieling, I., Van Rens, G. H. M. B., Wittebol-Post, D., Smeitink, J. A. M., De Jager, J. P., De Klerk, H. B. C., & Van Lith, G. H. M. (1997). Retinal dystrophy in long chain 3-hydroxy-acyl-coA dehydrogenase deficiency. British Journal of Ophthalmology, 81(4), 291–294. https://doi.org/10.1136/BJO.81.4.291

Schwab, K. O., Ensenauer, R., Matern, D., Uyanik, G., Schnieders, B., Wanders, R. J. A., & Lehnert, W. (2003). Complete deficiency of mitochondrial trifunctional protein due to a novel mutation within the β-subunit of the mitochondrial trifunctional protein gene leads to failure of long-chain fatty acid β-oxidation with fatal outcome. European Journal of Pediatrics, 162(2), 90–95. https://doi.org/10.1007/s00431-002-1035-4

Schwantje, M., Ebberink, M. S., Doolaard, M., Ruiter, J. P. N., Fuchs, S. A., Darin, N., Hedberg-Oldfors, C., Régal, L., Donker Kaat, L., Huidekoper, H. H., Olpin, S., Cole, D., Moat, S. J., Visser, G., & Ferdinandusse, S. (2022). Thermo-sensitive mitochondrial trifunctional protein deficiency presenting with episodic myopathy. Journal of Inherited Metabolic Disease, 45(4), 819–831. https://doi.org/10.1002/jimd.12503

Schwantje, M., Fuchs, S. A., de Boer, L., Bosch, A. M., Cuppen, I., Dekkers, E., Derks, T. G. J., Ferdinandusse, S., Ijlst, L., Houtkooper, R. H., Maase, R., van der Pol, W. L., de Vries, M. C., Verschoof-Puite, R. K., Wanders, R. J. A., Williams, M., Wijburg, F., & Visser, G. (2022). Genetic, biochemical, and clinical spectrum of patients with mitochondrial trifunctional protein deficiency identified after the introduction of newborn screening in the Netherlands. Journal of Inherited Metabolic Disease, 45(4), 804–818. https://doi.org/10.1002/JIMD.12502

Scott Schwoerer, J., Cooper, G., & Van Calcar, S. (2015). Rhabdomyolysis in a neonate due to very long chain acyl CoA dehydrogenase deficiency. Molecular Genetics and Metabolism Reports, 3, 39–41. https://doi.org/10.1016/J.YMGMR.2015.03.003

Sedgwick, E. R. (2013). Early Presentation of Very Long Chain Acyl-CoA Dehydrogenase Deficiency: Nursing Action Resulting in a Positive Outcome. Journal of Pediatric Nursing, 28(4), 379–382. https://doi.org/10.1016/j.pedn.2012.11.002

Shahrokhi, M., Shafiei, M., Galehdari, H., & Shariati, G. (2017). Identification of a novel HADHB gene mutation in an Iranian patient with mitochondrial trifunctional protein deficiency. Archives of Iranian Medicine, 20(1), 22–27.

Shaji Thomas, D., K Y, D., & Arulappan, J. (2021). Health Related Quality of Life of Caregivers of Children and Adolescents With Phenylketonuria: A Systematic Review. Global Pediatric Health, 8, 2333794X211065333. https://doi.org/10.1177/2333794X211065333

Sharef, S. W., Al-Senaidi, K., & Joshi, S. N. (2013). Successful treatment of cardiomyopathy due to very long-chain Acyl-CoA dehydrogenase deficiency: First case report from Oman with literature review. Oman Medical Journal, 28(5), 354–356. https://doi.org/10.5001/OMJ.2013.101

Shchelochkov, O., Wong, L. J., Shaibani, A., & Shinawi, M. (2009). Atypical presentation of VLCAD deficiency associated with a novel ACADVL splicing mutation. Muscle and Nerve, 39(3), 374–382. https://doi.org/10.1002/MUS.21157

Shelihan, I., Rossignol, E., Décarie, J. C., Bonnefont, J. P., Brivet, M., Brunel-Guitton, C., & Mitchell, G. A. (2022). Infantile onset carnitine palmitoyltransferase 2 deficiency: Cortical polymicrogyria, schizencephaly, and gray matter heterotopias in an adolescent with normal development. JIMD Reports, 63(1), 3–10. https://doi.org/10.1002/JMD2.12243

Shiraishi, H., Yamada, K., Oki, E., Ishige, M., Fukao, T., Hamada, Y., Sakai, N., Ochi, F., Watanabe, A., Kawakami, S., Kuzume, K., Watanabe, K., Sameshima, K., Nakamagoe, K., Tamaoka, A., Asahina, N., Yokoshiki, S., Miyakoshi, T., Oba, K., … Sato, N. (2019). Open-label clinical trial of bezafibrate treatment in patients with fatty acid oxidation disorders in Japan; 2nd report QOL survey. Molecular Genetics and Metabolism Reports, 20. https://doi.org/10.1016/J.YMGMR.2019.100496

Sim, K. G., Hammond, J., & Wilcken, B. (2002). Strategies for the diagnosis of mitochondrial fatty acid β-oxidation disorders. Clinica Chimica Acta, 323(1–2), 37–58. https://doi.org/10.1016/S0009-8981(02)00182-1

Sim, K. G., Wiley, V., Carpenter, K., & Wilcken, B. (2001). Carnitine palmitoyltransferase I deficiency in neonate identified by dried blood spot free carnitine and acylcarnitine profile. Journal of Inherited Metabolic Disease, 24(1), 51–59. https://doi.org/10.1023/A:1005606805951

Sims, H. F., Brackett, J. C., Powell, C. K., Treem, W. R., Hale, D. E., Bennett, M. J., Gibson, B., Shapiro, S., & Strauss, A. W. (1995). The molecular basis of pediatric long chain 3-hydroxyacyl-CoA dehydrogenase deficiency associated with maternal acute fatty liver of pregnancy. Proceedings of the National Academy of Sciences of the United States of America, 92(3), 841–845. https://doi.org/10.1073/PNAS.92.3.841

Sinclair, G. B., Collins, S., Popescu, O., McFadden Md, D., Arbour, L., & Vallance, H. D. (2012). Carnitine palmitoyltransferase I and sudden unexpected infant death in British Columbia First Nations. Pediatrics, 130(5). https://doi.org/10.1542/PEDS.2011-2924

Siu, W. K., Mak, C. M., Siu, S. L. Y., Siu, T. S., Pang, C. Y., Lam, C. W., Kwong, N. S., & Chan, A. Y. W. (2012). Molecular diagnosis for a fatal case of very long-chain acyl-CoA dehydrogenase deficiency in Hong Kong Chinese with a novel mutation: A preventable death by newborn screening. Diagnostic Molecular Pathology, 21(3), 184–187. https://doi.org/10.1097/PDM.0B013E31825554D0

Skladal, D., Sass, J. O., Geiger, H., Geiger, R., Mann, C., Vreken, P., Wanders, R. J. A., & Trawöger, R. (2000). Complications in Early Diagnosis and Treatment of Two Infants With Long-Chain Fatty Acid β-Oxidation Defects. Journal of Pediatric Gastroenterology and Nutrition, 31(4), 448–452. https://doi.org/10.1097/00005176-200010000-00023

Sklirou, E., Alodaib, A. N., Dobrowolski, S. F., Mohsen, A. W. A., & Vockley, J. (2021). Physiological Perspectives on the Use of Triheptanoin as Anaplerotic Therapy for Long Chain Fatty Acid Oxidation Disorders. Frontiers in Genetics, 11. https://doi.org/10.3389/FGENE.2020.598760

Sluysmans, T., Tuerlinckx, D., Hubinont, C., Verellen-Dumoulin, C., Brivet, M., & Vianey-Saban, C. (1997). Very long chain acyl-coenzyme A dehydrogenase deficiency in two siblings: Evolution after prenatal diagnosis and prompt management. Journal of Pediatrics, 131(3), 444–446. https://doi.org/10.1016/S0022-3476(97)80073-X

Smeets, R. J. P., Smeitink, J. A. M., Semmekrot, B. A., Scholte, H. R., Wanders, R. J. A., & Van den Heuvel, L. P. W. J. (2003). A novel splice site mutation in neonatal carnitine palmitoyl transferase II deficiency. Journal of Human Genetics, 48(1), 8–13. https://doi.org/10.1007/s100380300001

Smon, A., Repic Lampret, B., Groselj, U., Zerjav Tansek, M., Kovac, J., Perko, D., Bertok, S., Battelino, T., & Trebusak Podkrajsek, K. (2018). Next generation sequencing as a follow-up test in an expanded newborn screening programme. Clinical Biochemistry, 52, 48–55. https://doi.org/10.1016/J.CLINBIOCHEM.2017.10.016

Sobhy, G. A., El-Shabrawi, M., & Safar, H. (2022). A New Perspective on the Quality of Life of Children with Glycogen Storage Diseases. Pediatric Gastroenterology, Hepatology & Nutrition, 25(4), 321–331. https://doi.org/10.5223/pghn.2022.25.4.321

Sperk, A., Mueller, M., & Spiekerkoetter, U. (2010). Outcome in six patients with mitochondrial trifunctional protein disorders identified by newborn screening. Molecular Genetics and Metabolism, 101(2–3), 205–207. https://doi.org/10.1016/J.YMGME.2010.07.003

Spiekerkoetter, U. (2007). Effects of a fat load and exercise on asymptomatic VLCAD deficiency. Journal of Inherited Metabolic Disease, 30(3), 405. https://doi.org/10.1007/S10545-007-0548-4

Spiekerkoetter, U. (2010). Mitochondrial fatty acid oxidation disorders: Clinical presentation of long-chain fatty acid oxidation defects before and after newborn screening. Journal of Inherited Metabolic Disease, 33(5), 527–532. https://doi.org/10.1007/S10545-010-9090-X

Spiekerkoetter, U., Bastin, J., Gillingham, M., Morris, A., Wijburg, F., & Wilcken, B. (2010). Current issues regarding treatment of mitochondrial fatty acid oxidation disorders. Journal of Inherited Metabolic Disease, 33(5), 555–561. https://doi.org/10.1007/S10545-010-9188-1

Spiekerkoetter, U., Bennett, M. J., Ben-Zeev, B., Strauss, A. W., & Tein, I. (2004). Peripheral neuropathy, episodic myoglobinuria, and respiratory failure in deficiency of the mitochondrial trifunctional protein. Muscle and Nerve, 29(1), 66–72. https://doi.org/10.1002/MUS.10500

Spiekerkoetter, U., Haussmann, U., Mueller, M., Ter Veld, F., Stehn, M., Santer, R., & Lukacs, Z. (2010). Tandem mass spectrometry screening for very long-chain acyl-CoA dehydrogenase deficiency: The value of second-tier enzyme testing. Journal of Pediatrics, 157(4), 668–673. https://doi.org/10.1016/J.JPEDS.2010.04.063

Spiekerkoetter, U., Khuchua, Z., Yue, Z., Bennett, M. J., & Strauss, A. W. (2004). General Mitochondrial Trifunctional Protein (TFP) Deficiency As A Result of Either α- or β-Subunit Mutations Exhibits Similar Phenotypes because Mutations in Either Subunit Alter TFP Complex Expression and Subunit Turnover. Pediatric Research, 55(2), 190–196. https://doi.org/10.1203/01.PDR.0000103931.80055.06

Spiekerkoetter, U., Khuchua, Z., Yue, Z., & Strauss, A. W. (2004). The early-onset phenotype of mitochondrial trifunctional protein deficiency: A lethal disorder with multiple tissue involvement. Journal of Inherited Metabolic Disease, 27(2), 294–296. https://doi.org/10.1023/B:BOLI.0000028839.57386.88

Spiekerkoetter, U., Lindner, M., Santer, R., Grotzke, M., Baumgartner, M. R., Boehles, H., Das, A., Haase, C., Hennermann, J. B., Karall, D., de Klerk, H., Knerr, I., Koch, H. G., Plecko, B., Röschinger, W., Schwab, K. O., Scheible, D., Wijburg, F. A., Zschocke, J., … Wendel, U. (2009a). Management and outcome in 75 individuals with long-chain fatty acid oxidation defects: Results from a workshop. Journal of Inherited Metabolic Disease, 32(4), 488–497. https://doi.org/10.1007/S10545-009-1125-9

Spiekerkoetter, U., Lindner, M., Santer, R., Grotzke, M., Baumgartner, M. R., Boehles, H., Das, A., Haase, C., Hennermann, J. B., Karall, D., de Klerk, H., Knerr, I., Koch, H. G., Plecko, B., Röschinger, W., Schwab, K. O., Scheible, D., Wijburg, F. A., Zschocke, J., … Wendel, U. (2009b). Treatment recommendations in long-chain fatty acid oxidation defects: Consensus from a workshop. Journal of Inherited Metabolic Disease, 32(4), 498–505. https://doi.org/10.1007/S10545-009-1126-8

Spiekerkoetter, U., Mueller, M., Cloppenburg, E., Motz, R., Mayatepek, E., Bueltmann, B., & Korenke, C. (2008). Intrauterine cardiomyopathy and cardiac mitochondrial proliferation in mitochondrial trifunctional protein (TFP) deficiency. Molecular Genetics and Metabolism, 94(4), 428–430. https://doi.org/10.1016/J.YMGME.2008.04.002

Spiekerkoetter, U., Mueller, M., Sturm, M., Hofmann, M., & Schneider, D. T. (2012). Lethal undiagnosed very long-chain Acyl-CoA dehydrogenase deficiency with mild C14-acylcarnitine abnormalities on newborn screening. JIMD Reports, 6, 113–115. https://doi.org/10.1007/8904_2012_129

Spiekerkoetter, U., Sun, B., Khuchua, Z., Bennett, M. J., & Strauss, A. W. (2003). Molecular and phenotypic heterogeneity in mitochondrial trifunctional protein deficiency due to β-subunit mutations. Human Mutation, 21(6), 598–607. https://doi.org/10.1002/humu.10211

Spiekerkoetter U, Sun B, Wanders R, Strauss A, & Wendel U. (2003). Asymptomatic very long-chain acyl-CoA dehydrogenase (VLCAD) deficiency detected by MS/MS-based newborn screening: A time bomb? Pediatric Research, 53(4), 256A.

Spiekerkoetter, U., Sun, B., Zytkovicz, T., Wanders, R., Strauss, A. W., & Wendel, U. (2003). MS/MS-based newborn and family screening detects asymptomatic patients with very-long-chain acyl-CoA dehydrogenase deficiency. Journal of Pediatrics, 143(3), 335–342. https://doi.org/10.1067/S0022-3476(03)00292-0

Spiekerkoetter, U., Tenenbaum, T., Heusch, A., & Wendel, U. (2003). Cardiomyopathy and pericardial effusion in infancy point to a fatty acid β-oxidation defect after exclusion of an underlying infection. Pediatric Cardiology, 24(3), 295–297. https://doi.org/10.1007/s00246-002-0277-2

Stinton, C., Fraser, H., Geppert, J., Johnson, R., Connock, M., Johnson, S., Clarke, A., & Taylor-Phillips, S. (2021). Newborn Screening for Long-Chain 3-Hydroxyacyl-CoA Dehydrogenase and Mitochondrial Trifunctional Protein Deficiencies Using Acylcarnitines Measurement in Dried Blood Spots—A Systematic Review of Test Accuracy. Frontiers in Pediatrics, 9. https://doi.org/10.3389/FPED.2021.606194

Strandqvist, A., Haglind, C. B., Zetterström, R. H., Nemeth, A., von Döbeln, U., Stenlid, M. H., & Nordenström, A. (2016). Neuropsychological development in patients with long-chain 3-hydroxyacyl-CoA dehydrogenase (LCHAD) deficiency. JIMD Reports, 28, 75–84. https://doi.org/10.1007/8904_2015_505

Strauss, A. W., Powell, C. K., Hale, D. E., Anderson, M. M., Ahuja, A., Brackett, J. C., & Sims, H. F. (1995). Molecular basis of human mitochondrial very-long-chain acyl-CoA dehydrogenase deficiency causing cardiomyopathy and sudden death in childhood. Proceedings of the National Academy of Sciences of the United States of America, 92(23), 10496–10500. https://doi.org/10.1073/PNAS.92.23.10496

Sturm, V. (2008). Ophthalmologic abnormalities in long-chain 3-hydroxyacyl-CoA dehydrogenase deficiency: Presentation of a long-term survivor. European Journal of Ophthalmology, 18(3), 476–478. https://doi.org/10.1177/112067210801800330

Sugihara, K., Yuasa, M., Isozaki, Y., Hata, I., Ohshima, Y., Hamazaki, T., Kakiuchi, T., Arao, M., Igarashi, N., Kotani, Y., Fukuda, T., Kagawa, R., Tajima, G., & Shigematsu, Y. (2022). Severity estimation of very-long-chain acyl-CoA dehydrogenase deficiency via 13C-fatty acid loading test. Pediatric Research, 92(5), 1391–1399. https://doi.org/10.1038/S41390-022-01979-Z

Suyama, T., Shimura, M., Fushimi, T., Kuranobu, N., Ichimoto, K., Matsunaga, A., Takayanagi, M., & Murayama, K. (2020). Efficacy of bezafibrate in two patients with mitochondrial trifunctional protein deficiency. Molecular Genetics and Metabolism Reports, 24. https://doi.org/10.1016/J.YMGMR.2020.100610

Sykut-Cegielska, J., Gradowska, W., Piekutowska-Abramczuk, D., Andresen, B. S., Olsen, R. K. J., Ołtarzewski, M., Pronicki, M., Pajdowska, M., Bogdańska, A., Jabłońska, E., Radomyska, B., Kuśmierska, K., Krajewska-Walasek, M., Gregersen, N., & Pronicka, E. (2011). Urgent metabolic service improves survival in long-chain 3-hydroxyacyl-CoA dehydrogenase (LCHAD) deficiency detected by symptomatic identification and pilot newborn screening. Journal of Inherited Metabolic Disease, 34(1), 185–195. https://doi.org/10.1007/S10545-010-9244-X

Tajima, G., Hara, K., Tsumura, M., Kagawa, R., Okada, S., Sakura, N., Maruyama, S., Noguchi, A., Awaya, T., Ishige, M., Ishige, N., Musha, I., Ajihara, S., Ohtake, A., Naito, E., Hamada, Y., Kono, T., Asada, T., Sasai, H., … Kobayashi, M. (2017). Newborn screening for carnitine palmitoyltransferase II deficiency using (C16 + C18:1)/C2: Evaluation of additional indices for adequate sensitivity and lower false-positivity. Molecular Genetics and Metabolism, 122(3), 67–75. https://doi.org/10.1016/J.YMGME.2017.07.011

Tajima, G., Hara, K., & Yuasa, M. (2019). Carnitine palmitoyltransferase II deficiency with a focus on newborn screening. Journal of Human Genetics, 64(2), 87–98. https://doi.org/10.1038/S10038-018-0530-Z

Tajima, G., Sakura, N., Shirao, K., Okada, S., Tsumura, M., Nishimura, Y., Ono, H., Hasegawa, Y., Hata, I., Naito, E., Yamaguchi, S., Shigematsu, Y., & Kobayashi, M. (2008). Development of a new enzymatic diagnosis method for very-long-chain acyl-CoA dehydrogenase deficiency by detecting 2-hexadecenoyl-CoA production and its application in tandem mass spectrometry-based selective screening and newborn screening in Japan. Pediatric Research, 64(6), 667–672. https://doi.org/10.1203/PDR.0B013E318187CC44

Tan, J., Chen, D., Chang, R., Pan, L., Yang, J., Yuan, D., Huang, L., Yan, T., Ning, H., Wei, J., & Cai, R. (2021). Tandem Mass Spectrometry Screening for Inborn Errors of Metabolism in Newborns and High-Risk Infants in Southern China: Disease Spectrum and Genetic Characteristics in a Chinese Population. Frontiers in Genetics, 12. https://doi.org/10.3389/FGENE.2021.631688

Tang, C., Liu, S., Wu, M., Lin, S., Lin, Y., Su, L., Zhang, J., Feng, Y., & Huang, Y. (2019). Clinical and molecular characteristics of carnitine-acylcarnitine translocase deficiency: Experience with six patients in Guangdong China. Clinica Chimica Acta, 495, 476–480. https://doi.org/10.1016/J.CCA.2019.05.018

Tangeraas, T., Sæves, I., Klingenberg, C., Jørgensen, J., Kristensen, E., Gunnarsdottir, G., Hansen, E. V., Strand, J., Lundman, E., Ferdinandusse, S., Salvador, C. L., Woldseth, B., Bliksrud, Y. T., Sagredo, C., Olsen, Ø. E., Berge, M. C., Trømborg, A. K., Ziegler, A., Zhang, J. H., … Pettersen, R. D. (2020). Performance of expanded newborn screening in norway supported by post-analytical bioinformatics tools and rapid second-tier DNA analyses. International Journal of Neonatal Screening, 6(3). https://doi.org/10.3390/IJNS6030051

Tein, I., Donner, E. J., Hale, D. E., & Gordon Murphy, E. (1995). Clinical and neurophysiologic response of myopathy and neuropathy in long-chain l-3-hydroxyacyl-CoA dehydrogenase deficiency to oral prednisone. Pediatric Neurology, 12(1), 68–76. https://doi.org/10.1016/0887-8994(94)00109-F

Tenopoulou, M., Chen, J., Bastin, J., Bennett, M. J., Ischiropoulos, H., & Doulias, P.-T. (2015). Strategies for Correcting Very Long Chain Acyl-CoA Dehydrogenase Deficiency. Journal of Biological Chemistry, 290(16), 10486–10494. https://doi.org/10.1074/jbc.M114.635102

ter Veld, F., Mueller, M., Kramer, S., Haussmann, U., Herebian, D., Mayatepek, E., Laryea, M. D., Primassin, S., & Spiekerkoetter, U. (2009). A novel tandem mass spectrometry method for rapid confirmation of medium- and very long-chain acyl-CoA dehydrogenase deficiency in newborns. PLoS ONE, 4(7). https://doi.org/10.1371/JOURNAL.PONE.0006449

Tercanli, S., Uyanik, G., Hösli, I., Cagdas, A., & Holzgreve, W. (2000). Increased nuchal translucency in a case of long-chain 3-hydroxyacyl-coenzyme A dehydrogenase deficiency. Fetal Diagnosis and Therapy, 15(6), 322–325. https://doi.org/10.1159/000021029

Terrone, G., Ruoppolo, M., Brunetti-Pierri, N., Cozzolino, C., Scolamiero, E., Parenti, G., Romano, A., Andria, G., Salvatore, F., & Frisso, G. (2014). Child neurology: Recurrent rhabdomyolysis due to a fatty acid oxidation disorder. Neurology, 82(1). https://doi.org/10.1212/01.WNL.0000438217.31437.72

Thangavelu, S. (2010). Fatty Acid Oxidation disorders. Indian Journal of Practical Pediatrics, 12(2), 181–183. https://doi.org/10.21037/atm.2018.10.57

Thuillier, L., Belbachir, H., Royer-Legrain, G., Attie-Bitach, T., Driben, A., Abadi, N., Kamoun, P., Saudubray, J. M., Munnich, A., & Bonnefont, J. P. (1999). Molecular prenatal diagnosis of carnitine palmitoyltransferase 2 deficiency. American Society of Human Genetics 49th Annual Meeting.

Thuillier, L., Rostane, H., Droin, V., Demaugre, F., Brivet, M., Kadhom, N., Prip-Buus, C., Gobin, S., Saudubray, J. M., & Bonnefont, J. P. (2003). Correlation between genotype, metabolic data, and clinical presentation in carnitine palmitoyltransferase 2 (CPT2) deficiency. Human Mutation, 21(5), 493–501. https://doi.org/10.1002/humu.10201

Tolun, A., Devanapalli, B., Ip, W. C., Hertzog, A., Wiley, V., Bennetts, B., Lichkus, K., Grech, A., Paramore, C., & Bhattacharya, K. (2019). Carnitine palmitoyltransferase deficiency - A difficult disorder for newborn screening. 43rd Human Genetics Society of Australasia Annual Scientific Meeting.

Touma, E. H., Rashed, M. S., Vianey-Saban, C., Sakr, A., Divry, P., Gregersen, N., & Andresen, B. S. (2001). A severe genotype with favourable outcome in very long chain acyl-CoA dehydrogenase deficiency. Archives of Disease in Childhood, 84(1), 58–60. https://doi.org/10.1136/ADC.84.1.58

Treem, W. R., Stanley, C. A., Hale, D. E., Leopold, H. B., & Hyams, J. S. (1991). Hypoglycemia, hypotonia, and cardiomyopathy: The evolving clinical picture of long-chain acyl-CoA dehydrogenase deficiency. Pediatrics, 87(3), 328–333. https://doi.org/10.1542/peds.87.3.328

Tripp, M. E., & Shug, A. L. (1984). Plasma carnitine concentrations in cardiomyopathy patients. Biochemical Medicine, 32(2), 199–206. https://doi.org/10.1016/0006-2944(84)90073-5

Turaka, K., Bryan, J. S., Gordon, A. J., Kwong, H. M., Reddy, R., Tsipursky, M., & Sell, C. H. (2012). Clinical and image-guided chorioretinal findings in long-chain 3-hydroxyacyl-coenzyme A dehydrogenase deficiency. Journal of Pediatric Endocrinology and Metabolism, 25(5–6), 565–567. https://doi.org/10.1515/JPEM-2012-0019

Tuuli, I., Emilia, A., Jussi, T., Risto, L., Tiina, T., & Leena, L. (2016a). Peripheral neuropathy in patients with long-chain 3-hydroxyacyl-CoA dehydrogenase deficiency - A follow-up EMG study of 12 patients. European Journal of Paediatric Neurology, 20(1), 38–44. https://doi.org/10.1016/J.EJPN.2015.10.009

Tuuli, I., Emilia, A., Jussi, T., Risto, L., Tiina, T., & Leena, L. (2016b). Peripheral neuropathy in patients with long-chain 3-hydroxyacyl-CoA dehydrogenase deficiency - A follow-up EMG study of 12 patients. European Journal of Paediatric Neurology, 20(1), 38–44. https://doi.org/10.1016/j.ejpn.2015.10.009

Tyni, T., Immonen, T., Lindahl, P., Majander, A., & Kivel, T. (2012). Refined staging for chorioretinopathy in long-chain 3-hydroxyacyl coenzyme a dehydrogenase deficiency. Ophthalmic Research, 48(2), 75–81. https://doi.org/10.1159/000334874

Tyni, T., Kivelä, T., Lappi, M., Summanen, P., Nikoskelainen, E., & Pihko, H. (1998). Ophthalmologic findings in long-chain 3-hydroxyacyl-CoA dehydrogenase deficiency caused by the G1528C mutation: A new type of hereditary metabolic chorioretinopathy. Ophthalmology, 105(5), 810–824. https://doi.org/10.1016/S0161-6420(98)95019-9

Tyni, T., Palotie, A., Viinikka, L., Valanne, L., Salo, M. K., Von Dobeln, U., Jackson, S., Wanders, R., Venizelos, N., & Pihko, H. (1997). Long-chain 3-hydroxyacyl-coenzyme A dehydrogenase deficiency with the G1528C mutation: Clinical presentation of thirteen patients. Journal of Pediatrics, 130(1), 67–76. https://doi.org/10.1016/S0022-3476(97)70312-3

Tyni, T., & Pihko, H. (1999). Long-chain 3-hydroxyacyl-CoA dehydrogenase deficiency. Acta Paediatrica, International Journal of Paediatrics, 88(3), 237–245. https://doi.org/10.1080/08035259950169954

Vallejo, A. N., Mroczkowski, H. J., Michel, J. J., Woolford, M., Blair, H. C., Griffin, P., McCracken, E., Mihalik, S. J., Reyes-Mugica, M., & Vockley, J. (2021). Pervasive inflammatory activation in patients with deficiency in very-long-chain acyl-coA dehydrogenase (VLCADD). Clinical and Translational Immunology, 10(6). https://doi.org/10.1002/CTI2.1304

Van Calcar, S. C., Sowa, M., Rohr, F., Beazer, J., Setlock, T., Weihe, T. U., Pendyal, S., Wallace, L. S., Hansen, J. G., Stembridge, A., Splett, P., & Singh, R. H. (2020). Nutrition management guideline for very-long chain acyl-CoA dehydrogenase deficiency (VLCAD): An evidence- and consensus-based approach. Molecular Genetics and Metabolism, 131(1–2), 23–37. https://doi.org/10.1016/J.YMGME.2020.10.001

van Vliet, P., Berden, A. E., van Schie, M. K. M., Bakker, J. A., Heringhaus, C., de Coo, I. F. M., Langeveld, M., Schroijen, M. A., & Arbous, M. S. (2018). Peripheral neuropathy, episodic rhabdomyolysis, and hypoparathyroidism in a patient with mitochondrial trifunctional protein deficiency. JIMD Reports, 38, 101–105. https://doi.org/10.1007/8904_2017_37

Veenvliet, A. R. J., Garrelfs, M. R., Udink ten Cate, F. E. A., Ferdinandusse, S., Denis, S., Fuchs, S. A., Schwantje, M., Geurtzen, R., van Wegberg, A. M. J., Huigen, M. C. D. G., Kluijtmans, L. A. J., Wanders, R. J. A., Derks, T. G. J., de Boer, L., Houtkooper, R. H., de Vries, M. C., & van Karnebeek, C. D. M. (2022). Neonatal Long-Chain 3-Ketoacyl-CoA Thiolase deficiency: Clinical-biochemical phenotype, sodium-D,L-3-hydroxybutyrate treatment experience and cardiac evaluation using speckle echocardiography. Molecular Genetics and Metabolism Reports, 31. https://doi.org/10.1016/J.YMGMR.2022.100873

Vekemans, B. C., Bonnefont, J. P., Aupetit, J., Royer, G., Droin, V., Attié-Bitach, T., Saudubray, J. M., & Thuillier, L. (2003). Prenatal diagnosis of carnitine palmitoyltransferase 2 deficiency in chorionic villi: A novel approach. Prenatal Diagnosis, 23(11), 884–887. https://doi.org/10.1002/PD.713

Venema, A., Peeks, F., de Bruijn-van der Veen, M., de Boer, F., Fokkert-Wilts, M. J., Lubout, C. M. A., Huskens, B., Dumont, E., Mulkens, S., & Derks, T. G. J. (2022). A retrospective study of eating and psychosocial problems in patients with hepatic glycogen storage diseases and idiopathic ketotic hypoglycemia: Towards a standard set of patient-reported outcome measures. JIMD Reports, 63(1), 29–40. https://doi.org/10.1002/jmd2.12253

Vianey-Saban, C., Divry, P., Brivet, M., Nada, M., Zabot, M. T., Mathieu, M., & Roe, C. (1998). Mitochondrial very-long-chain acyl-coenzyme A dehydrogenase deficiency: Clinical characteristics and diagnostic considerations in 30 patients. Clinica Chimica Acta, 269(1), 43–62. https://doi.org/10.1016/S0009-8981(97)00185-X

Videen, J. S., Haseler, L. J., Karpinski, N. C., & Terkeltaub, R. A. (1999). Noninvasive evaluation of adult onset myopathy from carnitine palmitoyl transferase II deficiency using proton magnetic resonance spectroscopy. Journal of Rheumatology, 26(8), 1757–1763.

Vilarinho, L., Rocha, H., Sousa, C., Marcão, A., Fonseca, H., Bogas, M., & Osório, R. V. (2010). Four years of expanded newborn screening in Portugal with tandem mass spectrometry. Journal of Inherited Metabolic Disease, 33(SUPPL. 3). https://doi.org/10.1007/S10545-010-9048-Z

Vissing, J. (2016a). Dietary and other therapies in muscle glycogenoses and disorders of muscle lipid oxidation. 14th International Congress on Neuromuscular Diseases.

Vissing, J. (2016b). Exercise training in metabolic myopathies. Revue Neurologique, 172(10), 559–565. https://doi.org/10.1016/J.NEUROL.2016.08.005

Vitoria, I., Martín-Hernández, E., Peña-Quintana, L., Bueno, M., Quijada-Fraile, P., Dalmau, J., Molina-Marrero, S., Pérez, B., & Merinero, B. (2015). Carnitine-acylcarnitine translocase deficiency: Experience with four cases in spain and review of the literature. JIMD Reports, 20, 11–20. https://doi.org/10.1007/8904_2014_382

Vivante, A., Ityel, H., Pode-Shakked, B., Chen, J., Shril, S., van der Ven, A. T., Mann, N., Schmidt, J. M., Segel, R., Aran, A., Zeharia, A., Staretz-Chacham, O., Bar-Yosef, O., Raas-Rothschild, A., Landau, Y. E., Lifton, R. P., Anikster, Y., & Hildebrandt, F. (2017). Exome sequencing in Jewish and Arab patients with rhabdomyolysis reveals single-gene etiology in 43% of cases. Pediatric Nephrology, 32(12), 2273–2282. https://doi.org/10.1007/S00467-017-3755-8

Vladutiu, G. D., Bennett, M. J., Fisher, N. M., Smail, D., Boriack, R., Leddy, J., & Pendergast, D. R. (2002). Phenotypic variability among first-degree relatives with carnitine palmitoyltransferase II deficiency. Muscle and Nerve, 26(4), 492–498. https://doi.org/10.1002/MUS.10217

Vockley, J. (2020). Long-Chain Fatty Acid Oxidation Disorders and Current Management Strategies. American Journal of Managed Care, 26, S147–S154. https://doi.org/10.37765/AJMC.2020.88480

Vockley, J., Burton, B., Berry, G., Longo, N., Phillips, J., Sanchez-Valle, A., Chapman, K., Tanpaiboon, P., Grunewald, S., Murphy, E., Lu, X., & Cataldo, J. (2021). Effects of triheptanoin (UX007) in patients with long-chain fatty acid oxidation disorders: Results from an open-label, long-term extension study. Journal of Inherited Metabolic Disease, 44(1), 253–263. https://doi.org/10.1002/JIMD.12313

Vockley, J., Burton, B., Berry, G. T., Longo, N., Phillips, J., Sanchez‐Valle, A., Tanpaiboon, P., Grunewald, S., Murphy, E., Bowden, A., Chen, W., Chen, C., Cataldo, J., Marsden, D., & Kakkis, E. (2019). Results from a 78‐week, single‐arm, open‐label phase 2 study to evaluate UX007 in pediatric and adult patients with severe long‐chain fatty acid oxidation disorders (LC‐FAOD). Journal of Inherited Metabolic Disease, 42(1), 169–177. https://doi.org/10.1002/jimd.12038

Vockley, J., Burton, B., Berry, G. T., Longo, N., Phillips, J., Sanchez-Valle, A., Tanpaiboon, P., Grunewald, S., Murphy, E., Bowden, A., Chen, W., Chen, C. Y., Cataldo, J., Marsden, D., & Kakkis, E. (2019). Results from a 78-week, single-arm, open-label phase 2 study to evaluate UX007 in pediatric and adult patients with severe long-chain fatty acid oxidation disorders (LC-FAOD). Journal of Inherited Metabolic Disease, 42(1), 169–177. https://doi.org/10.1002/jimd.12038

Vockley, J., Burton, B., Berry, G. T., Longo, N., Phillips, J., Sanchez-Valle, A., Tanpaiboon, P., Grunewald, S., Murphy, E., Humphrey, R., Mayhew, J., Bowden, A., Zhang, L., Cataldo, J., Marsden, D. L., & Kakkis, E. (2017). UX007 for the treatment of long chain-fatty acid oxidation disorders: Safety and efficacy in children and adults following 24 weeks of treatment. Molecular Genetics and Metabolism, 120(4), 370–377. https://doi.org/10.1016/J.YMGME.2017.02.005

Vockley, J., Charrow, J., Ganesh, J., Eswara, M., Diaz, G. A., McCracken, E., Conway, R., Enns, G. M., Starr, J., Wang, R., Abdenur, J. E., Sanchez-de-Toledo, J., & Marsden, D. L. (2016). Triheptanoin treatment in patients with pediatric cardiomyopathy associated with long chain-fatty acid oxidation disorders. Molecular Genetics and Metabolism, 119(3), 223–231. https://doi.org/10.1016/J.YMGME.2016.08.008

Vockley, J., Enns, G. M., Ramirez, A. N., Bedrosian, C. L., Reineking, B., Lu, X., Ray, K., Rahman, S., & Marsden, D. (2022). Response to triheptanoin therapy in critically ill patients with LC-FAOD: Report of patients treated through an expanded access program. Molecular Genetics and Metabolism, 136(2), 152–162. https://doi.org/10.1016/J.YMGME.2022.04.001

Vockley, J., Longo, N., Madden, M., Dwyer, L., Mu, Y., Chen, C. Y., & Cataldo, J. (2021). Dietary management and major clinical events in patients with long-chain fatty acid oxidation disorders enrolled in a phase 2 triheptanoin study. Clinical Nutrition ESPEN, 41, 293–298. https://doi.org/10.1016/J.CLNESP.2020.11.018

Vockley, J., Marsden, D., McCracken, E., DeWard, S., Barone, A., Hsu, K., & Kakkis, E. (2015). Long-term major clinical outcomes in patients with long chain fatty acid oxidation disorders before and after transition to triheptanoin treatment-A retrospective chart review. Molecular Genetics and Metabolism, 116(1–2), 53–60. https://doi.org/10.1016/J.YMGME.2015.06.006

Vockley, J., Singh, R. H., & Whiteman, D. A. H. (2002). Diagnosis and management of defects of mitochondrial β-oxidation. Current Opinion in Clinical Nutrition and Metabolic Care, 5(6), 601–609. https://doi.org/10.1097/00075197-200211000-00002

Vorgerd, M. (2008). Therapeutic Options in Other Metabolic Myopathies. Neurotherapeutics, 5(4), 579–582. https://doi.org/10.1016/J.NURT.2008.08.006

Waisbren, S. E., Landau, Y., Wilson, J., & Vockley, J. (2013). Neuropsychological outcomes in fatty acid oxidation disorders: 85 cases detected by newborn screening. Developmental Disabilities Research Reviews, 17(3), 260–268. https://doi.org/10.1002/ddrr.1119

Walter, J. H. (2000). Inborn errors of metabolism and pregnancy. Journal of Inherited Metabolic Disease, 23(3), 229–236. https://doi.org/10.1023/A:1005679928521

Walter, J. H. (2009). Tolerance to fast: rational and practical evaluation in children with hypoketonaemia. Journal of Inherited Metabolic Disease, 32(2), 214–217. https://doi.org/10.1007/s10545-009-1087-y

Wanders, R. J. A. (2015). Prenatal Diagnosis of the Peroxisomal and Mitochondrial Fatty Acid Oxidation Deficiencies. In Genetic Disorders and the Fetus (pp. 838–856). <https://doi.org/https://doi.org/10.1002/9781118981559.ch21>

Wanders. R. J. A., Vaz, F. M., Ferdinandusse, S., van Kuilenburg, A. B. P., Kemp, S., van Karnebeek, C. D., Waterham, H. R., & Houtkooper. R. H. (2019) Translational Metabolism: A multidisciplinary approach towards precision diagnosis of inborn errors of metabolism in the omics era. *J Inherit Metab Dis,* 42, 197-208. doi: 10.1002/jimd.12008.

Wang, S., Wang, S., Leng, J., Diao, C., Wang, Y., Wang, Y., & Zheng, R. (2020). Genetic characteristics and follow-up of patients with fatty acid β-oxidation disorders through expanded newborn screening in a Northern Chinese population. Journal of Pediatric Endocrinology and Metabolism, 33(6), 683–690. https://doi.org/10.1515/JPEM-2019-0551

Wang, Y., Taroni, F., Garavaglia, B., & Longo, N. (2000). Functional analysis of mutations in the OCTN2 transporter causing primary carnitine deficiency: Lack of genotype-phenotype correlation. Human Mutation, 16(5), 401–407. https://doi.org/10.1002/1098-1004(200011)16:5<401::AID-HUMU4>3.0.CO;2-J

Watanabe, K., Yamada, K., Sameshima, K., & Yamaguchi, S. (2018). Two siblings with very long-chain acyl-CoA dehydrogenase (VLCAD) deficiency suffered from rhabdomyolysis after l -carnitine supplementation. Molecular Genetics and Metabolism Reports, 15, 121–123. https://doi.org/10.1016/j.ymgmr.2018.03.007

Welsink-Karssies, M. M., Polderman, J. A. W., Nieveen van Dijkum, E. J., Preckel, B., Schlack, W. S., Visser, G., Hollak, C. E., & Hermanides, J. (2017). Very Long-Chain Acyl-Coenzyme A Dehydrogenase Deficiency and Perioperative Management in Adult Patients. JIMD Reports, 34, 49–54. https://doi.org/10.1007/8904_2016_6

Wilcken, B. (2010). Fatty acid oxidation disorders: Outcome and long-term prognosis. Journal of Inherited Metabolic Disease, 33(5), 501–506. https://doi.org/10.1007/S10545-009-9001-1

Williams-Hall, R., Tinsley, K., Kruger, E., Johnson, C., Bowden, A., Cimms, T., & Gater, A. (2022). Qualitative evaluation of the symptoms and quality of life impacts of long-chain fatty acid oxidation disorders. Therapeutic Advances in Endocrinology and Metabolism, 13. https://doi.org/10.1177/20420188211065655

Winter, S. C. (2003). Treatment of carnitine deficiency. Journal of Inherited Metabolic Disease, 26(2–3), 171–180. https://doi.org/10.1023/a:1024433100257

Woerner, A. C., Gallagher, R. C., Vockley, J., & Adhikari, A. N. (2021). The Use of Whole Genome and Exome Sequencing for Newborn Screening: Challenges and Opportunities for Population Health. Frontiers in Pediatrics, 9, 663752. https://doi.org/10.3389/fped.2021.663752

Wongkittichote, P., Watson, J. R., Leonard, J. M., Toolan, E. R., Dickson, P. I., & Grange, D. K. (2020). Fatal COVID-19 infection in a patient with long-chain 3-hydroxyacyl-CoA dehydrogenase deficiency: A case report. JIMD Reports, 56(1), 40–45. https://doi.org/10.1002/JMD2.12165

Wood, J. C., Magera, M. J., Rinaldo, P., Seashore, M. R., Strauss, A. W., & Friedman, A. (2001). Diagnosis of very long chain acyl-dehydrogenase deficiency from an infant’s newborn screening card. Pediatrics, 108(1). https://doi.org/10.1542/PEDS.108.1.E19

Yagi, M., Lee, T., Awano, H., Tsuji, M., Tajima, G., Kobayashi, H., Hasegawa, Y., Yamaguchi, S., Takeshima, Y., & Matsuo, M. (2011). A patient with mitochondrial trifunctional protein deficiency due to the mutations in the HADHB gene showed recurrent myalgia since early childhood and was diagnosed in adolescence. Molecular Genetics and Metabolism, 104(4), 556–559. https://doi.org/10.1016/J.YMGME.2011.09.025

Yahyaoui, R., Espinosa, M. G., Gómez, C., Dayaldasani, A., Rueda, I., Roldán, A., Ugarte, M., Lastra, G., & Pérez, V. (2011). Neonatal carnitine palmitoyltransferase II deficiency associated with Dandy-Walker syndrome and sudden death. Molecular Genetics and Metabolism, 104(3), 414–416. https://doi.org/10.1016/J.YMGME.2011.05.003

Yamada, K. A., McHowat, J., Yan, G. X., Donahue, K., Peirick, J., Kléber, A. G., & Corr, P. B. (1994). Cellular uncoupling induced by accumulation of long-chain acylcarnitine during ischemia. Circulation Research, 74(1), 83–95. https://doi.org/10.1161/01.res.74.1.83

Yamada, K., Bo, R., Kobayashi, H., Hasegawa, Y., Ago, M., Fukuda, S., Yamaguchi, S., & Taketani, T. (2017). A newborn case with carnitine palmitoyltransferase II deficiency initially judged as unaffected by acylcarnitine analysis soon after birth. Molecular Genetics and Metabolism Reports, 11, 59–61. https://doi.org/10.1016/J.YMGMR.2017.04.008

Yamada, K., Shiraishi, H., Oki, E., Ishige, M., Fukao, T., Hamada, Y., Sakai, N., Ochi, F., Watanabe, A., Kawakami, S., Kuzume, K., Watanabe, K., Sameshima, K., Nakamagoe, K., Tamaoka, A., Asahina, N., Yokoshiki, S., Miyakoshi, T., Ono, K., … Sato, N. (2018). Open-label clinical trial of bezafibrate treatment in patients with fatty acid oxidation disorders in Japan. Molecular Genetics and Metabolism Reports, 15, 55–63. https://doi.org/10.1016/J.YMGMR.2018.02.003

Yamada, K., & Taketani, T. (2019). Management and diagnosis of mitochondrial fatty acid oxidation disorders: focus on very-long-chain acyl-CoA dehydrogenase deficiency. Journal of Human Genetics, 64(2), 73–85. https://doi.org/10.1038/S10038-018-0527-7

Yamaguchi, S., Hasegawa, Y., Bo, R., Yamada, K., Kobayashi, H., Taketani, T., & Fukuda, S. (2015). Prenatal diagnosis on 143 cases with organic and fatty acid diseases by biochemical and gene analyses with amniocentesis. Society for the Study of Inborn Errors of Metabolism Annual Symposium.

Yamaguchi, S., Hasegawa, Y., Yamada, K., Bo, R., Kobayashi, H., & Taketani, T. (2017). Prenatal diagnosis for severe cases of targets in expanded newborn screening. The 12th Asia-Pacific Conference on Human Genetics.

Yamak, A. A., Bitar, F., Karam, P., & Nemer, G. (2007). Exclusive cardiac dysfunction in familial primary carnitine deficiency cases: A genotype-phenotype correlation [1]. Clinical Genetics, 72(1), 59–62. https://doi.org/10.1111/J.1399-0004.2007.00814.X

Yamamoto, A., Nakamura, K., Matsumoto, S., Iwai, M., Shigematsu, Y., Tajima, G., Tsumura, M., Okada, S., Mitsubuchi, H., & Endo, F. (2013). VLCAD deficiency in a patient who recovered from ventricular fibrillation, but died suddenly of a respiratory syncytial virus infection. Pediatrics International, 55(6), 775–778. https://doi.org/10.1111/PED.12111

Yamamoto, H., Tachibana, D., Tajima, G., Shigematsu, Y., Hamasaki, T., Tanaka, A., & Koyama, M. (2015). Successful management of pregnancy with very-long-chain acyl-coenzyme A dehydrogenase deficiency. The Journal of Obstetrics and Gynaecology Research, 41(7), 1126–1128. https://doi.org/10.1111/jog.12672

Yamamoto, T., Tanaka, H., Kobayashi, H., Okamura, K., Tanaka, T., Emoto, Y., Sugimoto, K., Nakatome, M., Sakai, N., Kuroki, H., Yamaguchi, S., & Matoba, R. (2011). Retrospective review of Japanese sudden unexpected death in infancy: The importance of metabolic autopsy and expanded newborn screening. Molecular Genetics and Metabolism, 102(4), 399–406. https://doi.org/10.1016/J.YMGME.2010.12.004

Yan, H. M., Hu, H., Ahmed, A., Feng, B. B., Liu, J., Jia, Z. J., & Wang, H. (2017). Carnitine-acylcarnitine translocase deficiency with c.199-10 T>G and novel c.1A>G mutation. Medicine (United States), 96(45). https://doi.org/10.1097/MD.0000000000008549

Yang, B. Z., Mallory, J. M., Roe, D. S., Brivet, M., Strobel, G. D., Jones, K. M., Ding, J. H., & Roe, C. R. (2001). Carnitine/acylcarnitine translocase deficiency (neonatal phenotype): Successful prenatal and postmortem diagnosis associated with a novel mutation in a single family. Molecular Genetics and Metabolism, 73(1), 64–70. https://doi.org/10.1006/MGME.2001.3162

Yang, X., Li, Q., Wang, F., Yan, L., Zhuang, D., Qiu, H., Li, H., & Chen, L. (2021). Newborn Screening and Genetic Analysis Identify Six Novel Genetic Variants for Primary Carnitine Deficiency in Ningbo Area, China. Frontiers in Genetics, 12. https://doi.org/10.3389/FGENE.2021.686137

Yang, Z., Yamada, J., Zhao, Y., Strauss, A. W., & Ibdah, J. A. (2002). Prospective screening for pediatric mitochondrial trifunctional protein defects in pregnancies complicated by liver disease. JAMA, 288(17), 2163–2166. https://doi.org/10.1001/jama.288.17.2163

Yang, Z., Zhao, Y., Bennett, M. J., Strauss, A. W., & Ibdah, J. A. (2002). Fetal genotypes and pregnancy outcomes in 35 families with mitochondrial trifunctional protein mutations. American Journal of Obstetrics and Gynecology, 187(3), 715–720. https://doi.org/10.1067/MOB.2002.125893

Yanus, G. A., Akhapkina, T. A., Whitehead, A. J., Bizin, I. V., Iyevleva, A. G., Kuligina, E. S., Aleksakhina, S. N., Anisimova, M. O., Holmatov, M. M., Romanko, A. A., Zaitseva, O. A., Yatsuk, O. S., Zagorodnev, K. A., Matsneva, M. A., Koloskov, A. V., Togo, A. V., Suspitsin, E. N., & Imyanitov, E. N. (2019). Exome-based search for recurrent disease-causing alleles in Russian population. European Journal of Medical Genetics, 62(7). https://doi.org/10.1016/J.EJMG.2019.04.013

Yasuno, T., Kaneoka, H., Tokuyasu, T., Aoki, J., Yoshida, S., Takayanagi, M., Ohtake, E., Kanazawa, M., Ogawa, A., Tojo, K., & Saito, T. (2008). Mutations of carnitine palmitoyltransferase II (CPT II) in Japanese patients with CPT II deficiency. Clinical Genetics, 73(5), 496–501. https://doi.org/10.1111/J.1399-0004.2008.00986.X

Yoon, H. R., Strauss, A. W., & Yoo, H. W. (2001). Sudden death in a Korean infant with very long-chain acyl-CoA dehydrogenase deficiency. Journal of Inherited Metabolic Disease, 24(3), 407–408. https://doi.org/10.1023/A:1010521105909

Yuasa, M., Hata, I., Sugihara, K., Isozaki, Y., Ohshima, Y., Hara, K., Tajima, G., & Shigematsu, Y. (2019). Evaluation of metabolic defects in fatty acid oxidation using peripheral blood mononuclear cells loaded with deuterium-labeled fatty acids. Disease Markers, 2019. https://doi.org/10.1155/2019/2984747

Zhang, R. N., Li, Y. F., Qiu, W. J., Ye, J., Han, L. S., Zhang, H. W., Lin, N., & Gu, X. F. (2014). Clinical features and mutations in seven Chinese patients with very long chain acyl-CoA dehydrogenase deficiency. World Journal of Pediatrics, 10(2), 119–125. https://doi.org/10.1007/S12519-014-0480-2

Zhou, W., Li, H., Huang, T., Zhang, Y., Wang, C., & Gu, M. (2019). Biochemical, molecular, and clinical characterization of patients with primary carnitine deficiency via large-scale newborn screening in Xuzhou area. Frontiers in Pediatrics, 7(FEB). https://doi.org/10.3389/FPED.2019.00050

Zöggeler, T., Stock, K., Jörg-Streller, M., Spenger, J., Konstantopoulou, V., Hufgard-Leitner, M., Scholl-Bürgi, S., & Karall, D. (2021). Long-term experience with triheptanoin in 12 Austrian patients with long-chain fatty acid oxidation disorders. Orphanet Journal of Rare Diseases, 16(1). https://doi.org/10.1186/S13023-020-01635-X

Zweers, H., Timmer, C., Rasmussen, E., den Heijer, M., & de Valk, H. (2012). Successful weight loss in two adult patients diagnosed with late-onset long-chain fatty acid oxidation defect. JIMD Reports, 6, 127–129. https://doi.org/10.1007/8904_2012_131
